# Effect of antihypertensive deprescribing on hospitalisation and mortality: long-term follow-up of the OPTiMISE randomised controlled trial

**DOI:** 10.1016/S2666-7568(24)00131-4

**Published:** 2024-08

**Authors:** James P Sheppard, Eleanor Temple, Ariel Wang, Anne Smith, Stephanie Pollock, Gary A Ford, F D Richard Hobbs, Nicola Kenealy, Paul Little, Mark Lown, Simon de Lusignan, Jonathan Mant, David McCartney, Rupert A Payne, Marney Williams, Ly-Mee Yu, Richard J McManus, OPTiMISE Investigators

**Affiliations:** a Nuffield Department of Primary Care Health Sciences, University of Oxford, Oxford, UK; b Radcliffe Department of Medicine, University of Oxford, UK and Oxford University Hospitals NHS Foundation Trust, Oxford, UK; c Primary Care Research Group, University of Southampton, Southampton, UK; d Primary Care Unit, Department of Public Health and Primary Care, University of Cambridge, Cambridge, UK; e Health and Community Sciences, University of Exeter, Exeter, UK; f Patient and public involvement representative, London, UK

## Abstract

**Background:**

Deprescribing of antihypertensive medications is recommended for some older patients with low blood pressure and frailty. The OPTiMISE trial showed that this deprescribing can be achieved with no differences in blood pressure control at 3 months compared with usual care. We aimed to examine effects of deprescribing on longer-term hospitalisation and mortality.

**Methods:**

This randomised controlled trial enrolled participants from 69 general practices across central and southern England. Participants aged 80 years or older, with systolic blood pressure less than 150 mm Hg and who were receiving two or more antihypertensive medications, were randomly assigned (1:1) to antihypertensive medication reduction (removal of one antihypertensive) or usual care. General practitioners and participants were aware of the treatment allocation following randomisation but individuals responsible for analysing the data were masked to the treatment allocation throughout the study. Participants were followed up via their primary and secondary care electronic health records at least 3 years after randomisation. The primary outcome was time to all-cause hospitalisation or mortality. Intention-to-treat analyses were done using Cox regression modelling. A per-protocol analysis of the primary outcome was also done, excluding participants from the intervention group who did not reduce treatment or who had medication reinstated during the initial trial 12-week follow-up period. This study is registered with the European Union Drug Regulating Authorities Clinical Trials Database (EudraCT2016-004236-38) and the ISRCTN Registry (ISRCTN97503221).

**Findings:**

Between March 20, 2017, and Sept 30, 2018, a total of 569 participants were randomly assigned. Of these, 564 (99%; intervention=280; control=284) were followed up for a median of 4·0 years (IQR 3·7–4·3). Participants had a mean age of 84·8 years (SD 3·4) at baseline and 273 (48%) were women. Medication reduction was sustained in 109 participants at follow-up (51% of the 213 participants alive in the intervention group). Participants in the intervention group had a larger reduction in antihypertensives than the control group (adjusted mean difference –0·35 drugs [95% CI –0·52 to –0·18]). Overall, 202 (72%) participants in the intervention group and 218 (77%) participants in the control group experienced hospitalisation or mortality during follow-up (adjusted hazard ratio [aHR] 0·93 [95% CI 0·76 to 1·12]). There was some evidence that the proportion of participants experiencing the primary outcome in the per-protocol population was lower in the intervention group (aHR 0·80 [0·64 to 1·00]).

**Interpretation:**

Half of participants sustained medication reduction with no evidence of an increase in all-cause hospitalisation or mortality. These findings suggest that an antihypertensive deprescribing intervention might be safe for people aged 80 years or older with controlled blood pressure taking two or more antihypertensives.

**Funding:**

British Heart Foundation and National Institute for Health and Care Research.

## Introduction

Hypertension can be effectively treated with antihypertensive medication, which has been proven to lower the risk of stroke and cardiovascular diseases across all age groups.^1^ Even in patients older than 80 years, trials have shown that antihypertensive treatment reduces the risk of cardiovascular disease and all-cause mortality.^2^^,^^3^ However, treatment is not without the possibility of harm. A recent meta-analysis of previous trials showed that antihypertensives are associated with an increased risk of hypotension, syncope, acute kidney injury, and hyperkalaemia.^4^ Such events are typically more common in older people, with observational evidence suggesting a substantially increased risk in patients older than 80 years and in those with moderate to severe frailty.^5^

**Research in context**

**Evidence before this study**

We searched PubMed from inception until March 15, 2024, for previous randomised controlled trials published in any language using the search terms “frail elderly” and “antihypertensive” and “medication reduction”. We identified a Cochrane systematic review of previous randomised controlled trials published in 2020, and updated again in 2023, examining the effect of antihypertensive withdrawal in older people (≥50 years). This review identified just six relevant trials, including 1073 participants, but with short follow-up periods and very low numbers of outcome events, precluding any assessment of the effect of antihypertensive withdrawal on clinical outcomes. More recently, the Optimising Treatment for Mild Systolic Hypertension in the Elderly (OPTiMISE) trial examined the effects of withdrawing one antihypertensive compared with usual care in older patients with controlled systolic blood pressure. The study found that medication withdrawal could be sustained over a 12-week follow-up in 66% of those in the medication reduction intervention group, with no difference between groups in the proportion of patients with controlled blood pressure. However, neither this trial nor the previous Cochrane review were powered to detect differences in clinical outcomes such as cardiovascular disease or mortality. Two observational studies were identified that examined the effects of antihypertensive deprescribing on these outcomes. Both suggested that deprescribing is associated with worse clinical outcomes, although there were some methodological limitations that could have biased these results.

**Added value of this study**

To the best of our knowledge, this is the largest study of antihypertensive deprescribing conducted to date, and the first to follow up participants for more than 4 years and to examine the effects on hospitalisation, mortality, and major cardiovascular disease events. The study found that antihypertensive deprescribing was achieved and sustained in over half of those participants in the medication reduction intervention group, with no evidence of harm. These findings suggest that deprescribing antihypertensive medication might be safe in older patients living in the community with controlled blood pressure who were prescribed two or more antihypertensives.

**Implications of all the available evidence**

Some clinical guidelines for the management of hypertension now recommend that withdrawal of antihypertensive therapy be considered in older patients with low systolic blood pressure and clinically significant frailty. Until now, this recommendation was not based on robust empirical evidence and has been contradicted by some previous observational studies. This study provides support for antihypertensive deprescribing, showing that it can be sustained for 4 years in over half of those attempting it, with no evidence of harm. These findings suggest that deprescribing antihypertensive medication might be safe and could be used to reduce polypharmacy in older patients living with complex multiple long-term conditions in the community.

Accordingly, guidelines for the management of hypertension now recommend using clinical judgement when prescribing in older people with frailty,^6^^,^^7^ with some recommending that reduction of therapy (known as deprescribing) be considered in older patients with low systolic blood pressure (<120 mm Hg) and clinically significant frailty.^8^ This is in contrast to clinical trial evidence showing potential benefit of even intensive blood pressure lowering, and observational studies suggesting that withdrawing antihypertensives is associated with worse clinical outcomes.^9^, ^10^, ^11^

There have been very few randomised controlled trials examining outcomes of deprescribing antihypertensives. A Cochrane systematic review of previous studies published in 2020, and updated in 2023, found just six trials including 1073 participants, but with short follow-up periods and very low numbers of outcome events.^12^ Although the Optimising Treatment for Mild Systolic Hypertension in the Elderly (OPTiMISE) trial^13^ was not included in this review, this trial examined the effects of withdrawing one antihypertensive in older patients, aged 80 years and older, with controlled systolic blood pressure (<150 mm Hg). In 569 participants, 66% of those assigned to the intervention group maintained medication reduction over the 12-week follow-up period. Although the study was not originally powered to evaluate clinical outcomes such as cardiovascular disease or mortality, it did demonstrate that deprescribing antihypertensives was non-inferior to usual care with regard to systolic blood pressure control (relative risk 0·98 [95% CI 0·92 to ∞]), albeit with an increase in mean systolic blood pressure of 3 mm Hg at 12 weeks. These data alone therefore cannot be used to determine the safety of antihypertensive deprescribing in older patients with frailty.

The present study aimed to examine the longer-term effects of deprescribing antihypertensives, using routine electronic health records to follow up participants in the OPTiMISE trial and to determine the effects of the original deprescribing intervention on hospitalisation, mortality, major cardiovascular events, medication prescription, and blood pressure at least 3 years after randomisation.

## Methods

### Study design and participants

The design and methods of the OPTiMISE trial have been published previously,^13^^,^^14^ and the protocol is provided in the appendix (pp 4–66). Briefly, the trial enrolled participants aged 80 years or older, with systolic blood pressure less than 150 mm Hg at baseline and who were prescribed two or more antihypertensive medications, from 69 general practices across central and southern England. General practitioners were asked to enrol patients who, in their opinion, might benefit from medication reduction due to existing polypharmacy, comorbidity, non-adherence, dislike of medicines, or frailty. Detailed inclusion and exclusion criteria are provided in the appendix (p 129).

The study was approved by a National Health Service (NHS) Research Ethics Committee (South Central—Oxford A; 16/SC/0628) and the Medicines and Healthcare products Regulatory Agency (MHRA; 21584/0371/001-0001). All participants gave written informed consent. This study is registered with the International Standard Randomised Controlled Trial Number registry (ISRCTN97503221) and the European Union Drug Regulating Authorities Clinical Trials Database (EudraCT2016-004236-38).

### Randomisation and masking

Participants were randomly assigned (1:1) to one of the two study groups using a non-deterministic minimisation algorithm, designed to balance on practice and baseline systolic blood pressure, via a validated, web-based, password-protected system (Sortition, Nuffield Department of Primary Care Health Sciences, Oxford, UK). Those assigned to the intervention group had one antihypertensive withdrawn, with advice given to the treating general practitioner on appropriate drug choice. Those allocated to the control group followed usual care, where they continued to take all antihypertensive medications as prescribed with no medication changes mandated. General practitioners and participants were unaware of treatment allocation prior to consent and baseline assessments, but were aware of the treatment allocation after randomisation. Individuals responsible for analysing the data were masked to the treatment allocation.

### Procedures

Participants were actively followed up for 12 weeks, and attended one or two follow-up visits—one at 4 weeks post-randomisation (intervention group only, with drug reinstatement if systolic blood pressure was ≥150 mm Hg) and the other at 12 weeks (both groups), where blood pressure was measured for assessment of the original trial primary outcome (ie, systolic blood pressure control lower than 150 mm Hg). Randomisation was undertaken between March 20, 2017, and Sept 30, 2018. Original trial follow-up was completed on Jan 9, 2019. The study then continued in long-term follow-up via routine electronic health records until April 6, 2023.

All data were collected by a research facilitator or nurse in clinics held at baseline. Data relating to participant characteristics, prescribed medication, and medical history were collected directly from participants and from their electronic health records. Blood pressure was measured using the clinically validated BpTRU blood pressure monitor (BpTRU Medical Devices, Coquitlam, BC, Canada).^15^ Readings were taken in the left arm, using an appropriately sized cuff, after participants had been seated for at least 5 min of rest. Systolic blood pressure was estimated from the mean of the second and third readings. Assessments of functional independence and cognitive function were undertaken at baseline using the Modified Rankin scale^16^ and Montreal Cognitive Assessment^17^ screening tool, respectively. Frailty was defined using the Electronic Frailty Index.^18^ Quality of life was measured using the EQ-5D-5L.^19^

Passive follow-up was undertaken in all participants who had not withdrawn from the trial, using three approaches. Firstly, all participating general practices were visited by the research team, who undertook a manual review of each participant’s electronic health record, extracting relevant data into a follow-up case report form. Secondly, approval was given to link all participants to data held by NHS England relating to hospital admissions and deaths during follow-up (data sharing agreement DARS-NIC-459340-M8R2R-v0.11). Linking of data was achieved using a participant’s NHS number that was collected at baseline, which was encrypted and sent via secure data transfer to NHS England, which then extracted the data fields requested from datasets containing information about Hospital Episode Statistics (the commissioning dataset that consistently captures hospital activity such as diagnoses, procedures, and therapies nationally) and Civil Registrations of Death, as provided by the Office for National Statistics. These data were sent back to the research team via the same secure data transfer pathway. Thirdly, primary care data were extracted from the electronic health records of participants registered at primary care sites contributing to the Oxford Clinical Informatics Digital Hub (ORCHID).^20^ This system allows near real-time data collection from electronic health records including all coded data, prescriptions, test results, and coded diagnoses.^21^ Once again, relevant participant records were identified using encrypted participant NHS numbers. Participants were deemed not assessable only if their records could not be located using NHS numbers or other identifiable information held by the research team.

### Outcomes

The primary outcome of this long-term follow-up study was time to all-cause hospitalisation or mortality. All-cause hospitalisations were determined from data provided by NHS England. Where these data were not available (because trial participants could not be linked via their NHS number to their electronic health records), hospitalisations captured via the manual notes review were included. Mortality was determined from data provided by NHS England or the manual notes review.

Secondary outcomes were time to all-cause hospitalisation, all-cause mortality, emergency hospitalisation, and hospitalisation or mortality due to major cardiovascular events, stroke, or myocardial infarction. These were defined using data from NHS England. Emergency hospitalisation was defined as an admission preceded by a visit to the emergency department within the previous 48 h. Major cardiovascular events were defined as admission to hospital with non-fatal stroke, myocardial infarction, heart failure, or cardiovascular mortality, using prespecified ICD-10 codes documented in the electronic health records (see statistical analysis plan, appendix p 122).

Further secondary outcomes included hospitalisation due to a fall, syncope, hypotension, fracture, electrolyte abnormalities, and acute kidney injury, defined using data from NHS England, according to prespecified ICD-10 codes (see statistical analysis plan, appendix p 122). Diagnosis of dementia was also included based on data from the manual notes review and NHS England (the latter based on prespecified ICD-10 codes).

Maintenance of the treatment strategy assigned at baseline (medication reduction or usual care) was assessed, along with the difference between groups in the change in the number of antihypertensive medications, at 3-year follow-up, using data from the manual notes review. In those with data available from ORCHID, changes in antihypertensive medication prescription over time were examined in all participants and in those who were still alive at the end of follow-up. The change in systolic and diastolic blood pressure from baseline, and the proportion of participants with controlled blood pressure (<150/90 mm Hg), were also examined at 3-year follow-up (using data from the manual notes review) and over time using a subset of data from ORCHID in all participants and in those who were still alive at the end of follow-up. Finally, the difference in the number of primary care consultations related to hypertension during follow-up was examined, including consultations overall and consultations with general practitioners, nurses, pharmacists, and other health-care professionals.

### Statistical analysis

The sample size was determined by those enrolled in the original trial (n=569), minus anyone who expressed a wish to withdraw consent for participation during follow-up. Assuming an event rate of 9·9% per year for at least 3 years (based on rates observed during the 3-month follow-up period of the original trial),^13^ this sample size was deemed sufficient to detect a 58% increase (hazard ratio 1·58) in the rate of all-cause hospitalisation or death as a result of medication reduction, with an alpha of 0·05 and 90% power.

The statistical analysis plan is provided in the appendix (pp 67–128). All analyses followed an intention-to-treat principle, unless stated otherwise. We used a Cox proportional hazards model for the analysis of the primary outcome, adjusting for baseline systolic blood pressure and intervention group as fixed effects. Practice was included as a random effect (where convergence was possible). Model assumptions were checked through inspection of Schoenfeld residuals and survival curves. There was no evidence of violation of the proportional hazards assumption for any outcome. Other time-to-event outcomes were analysed using similar methods. In these analyses, all follow-up data were included and participants were censored if they experienced the outcome of interest, died, or were lost to follow-up.

A per-protocol analysis of the primary outcome was performed that excluded participants from the intervention group who did not reduce treatment or who had medication reinstated during the initial trial 12-week follow-up period (although this latter action was part of the medication reduction protocol).

The proportion of participants who maintained the treatment strategy to which they were randomly assigned, and the proportion of participants from both groups who had medications increased, were summarised descriptively. Further analyses comparing the adjusted mean difference of change in blood pressure and number of antihypertensive medications at 3 years from randomisation were performed using linear mixed effects models adjusting for baseline systolic blood pressure, with practice fitted as a random effect. The number of participants who had a hypertension-related primary care consultation was compared between groups using a mixed effect logistic regression model, adjusting for baseline systolic blood pressure and including practice as a random effect. The mean difference in the number of hypertension-related primary care consultations was derived from a negative binomial regression model, adjusting for baseline systolic blood pressure as a fixed effect and including practice as a random effect.

Sensitivity analyses were undertaken analysing the primary and secondary outcomes as counts of events, using a generalised linear Poisson mixed effects model that adjusted for randomised group and baseline systolic blood pressure and included practice as a random effect. For all binary outcomes, the analysis was repeated with events included only if they occurred within 3 years of randomisation. Exploratory subgroup analyses of the primary outcome, systolic blood pressure control, and change in systolic blood pressure were conducted by different levels of baseline frailty, functional independence, cognitive function, number of antihypertensive medications prescribed at baseline, and number of comorbidities.

To assess the potential impact of the COVID-19 pandemic, post-hoc analyses were undertaken exploring the incidence of outcome events by calendar year, as well as analyses restricted to outcomes that occurred before the pandemic (ie, before March 23, 2020). To explore the impact of sex, we assessed the interaction between the effects of medication reduction on the primary outcome and participant sex.

The level of significance for all analyses was 5% using the two-sided test procedure. P values were adjusted for multiple comparisons in order to maintain an overall type I error rate of 5%. All data were analysed using Stata statistical software (Stata/SE and MP version 18.0).

### Role of the funding source

The funders of the study had no role in study design, data collection, data analysis, data interpretation, or writing of the report.

## Results

A total of 6194 patients were invited to participate in the original trial by post and 739 (12%) attended a screening appointment (appendix p 130). Of these, 569 participants (77%) provided informed consent and were enrolled in the trial and randomly assigned. Participants were representative of those invited to participate in the trial with regard to age, sex, systolic blood pressure, and frailty.^22^ A total of 282 (50%) participants were assigned to the medication reduction intervention and 287 (50%) to usual care (appendix p 130).

Long-term follow-up via manual review of the primary care electronic health records was conducted between Aug 30, 2022, and April 6, 2023, and completed for 556 (98%) participants (appendix p 130). Hospital episode data were provided up until March 30, 2022, and were available for 554 (97%) participants. Civil registration death data were provided up until Jan 30, 2023, and were available for 554 (97%) participants. Primary care data were extracted from ORCHID up until March 29, 2023, and were available from 48 practices for 369 (65%) participants. The study database was locked on Oct 4, 2023. Overall, five participants were lost to follow-up, and therefore data for the primary outcome were available in 564 (99%) participants—280 in the intervention group and 284 in the control group.

For those participants who provided follow-up data, treatment groups were well matched for all variables at baseline, with a mean age of 84·8 years (SD 3·4); 273 (48%) participants were women (table 1). The majority of participants had complex multiple long-term conditions, polypharmacy, mild cognitive impairment, and mild frailty (table 1). Mean blood pressure at baseline was 130·0/69·3 mm Hg (SD 12·7/8·8) and individuals were taking an average of 2·5 antihypertensive medications (SD 0·6; range 2–5).

**Table 1:** Baseline characteristics

	Medication reduction group (n=280)	Usual care group (n=284)
Age, years	84·7 (3·3)	85·0 (3·6)
Age >85 years	130 (46%)	143 (50%)
Sex		
Female	130 (46%)	143 (50%)
Male	150 (54%)	141 (50%)
BMI, kg/m^2^	27·2 (4·2) [n=278]	28·0 (4·3) [n=271]
Ethnicity∗		
White British	273 (98%)	270 (95%)
Other ethnicity	7 (3%)	14 (5%)
Undergraduate or postgraduate degree obtained	44 (16%)	39 (14%)
Current smoker	3 (1%)	5 (2%)
Alcohol consumption (report drinking alcohol weekly)	98 (35%)	108 (38%)
Total cholesterol†, mmol/L	4·6 (1·2) [n=250]	4·6 (1·2) [n=256]
Montreal Cognitive Assessment score‡	24·4 (3·6) [n=278]	24·0 (4·1) [n=279]
EQ-5D-5L score§	0·8 (0·2) [n=277]	0·8 (0·2) [n=281]
Modified Rankin Scale score >2 (dependent)¶	35/267 (13%)	41/273 (15%)
Electronic Frailty Index (eFI) score||	0·14 (0·07)	0·15 (0·07)
Fit (eFI 0 to 0·12)	121 (43%)	109 (38%)
Mild frailty (eFI >0·12 to 0·24)	130 (46%)	140 (49%)
Moderate frailty (eFI >0·24 to 0·36)	27 (10%)	32 (11%)
Severe frailty (eFI >0·36)	2 (<1%)	3 (1%)
Blood pressure		
Systolic blood pressure, mm Hg	129·4 (13·2)	130·5 (12·3)
Diastolic blood pressure, mm Hg	68·4 (9·1)	70·1 (8·4)
History of high blood pressure, years	16·8 (8·9) [n=267]	16·4 (9·0) [n=273]
Standing systolic blood pressure, mm Hg	128·7 (15·6) [n=262]	131·8 (16·2) [n=258]
Orthostatic hypotension∗∗	15/262 (5%)	10/258 (4%)
Medical history††		
Chronic kidney disease	83 (30%)	102 (36%)
Cancer	66 (24%)	68 (24%)
Cardiac disease‡‡	60 (21%)	60 (21%)
Diabetes	48 (17%)	53 (19%)
Atrial fibrillation	45 (16%)	45 (16%)
Transient ischaemic attack	27 (10%)	22 (8%)
Stroke	23 (8%)	22 (8%)
Peripheral vascular disease	6 (2%)	9 (3%)
Total number of morbidities	5·7 (2·7)	6·0 (3·0)
Two or more morbidities	276 (99%)	279 (98%)
Medication prescriptions		
Any antihypertensive	280 (100%)	284 (100%)
ACE inhibitor	141 (50%)	155 (55%)
Angiotensin II receptor blocker	139 (50%)	128 (45%)
Calcium channel blockers	199 (71%)	190 (67%)
β blockers	111 (40%)	113 (40%)
Thiazide and related diuretics	108 (39%)	110 (39%)
Statin	184 (66%)	193 (68%)
Antiplatelet	223 (80%)	232 (82%)
Total antihypertensives	2 (2–3)	2 (2–3)
Total non-cardiovascular medications	1 (1–2)	1 (1–2)
Total prescribed medications	4 (3–7)	4 (3–7)

Data are mean (SD), n (%), or median (IQR). Where not all patients had available data, data are shown as n/N (%) or mean (SD) [number of patients with available data]. ACE=angiotensin-converting enzyme.

∗ Ethnic group was defined according to participant’s self-reported ethnicity, using Office for National Statistics categories.

† Most recently recorded reading from electronic health records.

‡ Score ranges between 0 and 30 with lower scores representing greater impairment. A score of 26 and over is considered to be normal.

§ The EQ-5D-5L assesses five aspects of health: mobility, self-care, activities, discomfort, and anxiety or depression. EQ-5D-5L index scores were generated using a crosswalk approach which translates the scores for the five EQ-5D-5L items into a single index value. The index value ranges from –0·594 (worse than death) to 1 (full health).

¶ Modified Rankin scale ranges from 0 (no symptoms) to 5 (severe disability).

|| The Electronic Frailty Index has 36 items and is estimated from electronic health records. The index ranges from 0 (fit) to 1 (frail).

∗∗ Orthostatic hypotension was defined as a decrease in systolic blood pressure of ≥20 mm Hg within 3 min of standing.

†† Individual conditions listed represent the eight most common conditions that are thought to be associated with high blood pressure.

‡‡ Cardiac disease was defined as the presence of myocardial infarction, coronary heart disease, angina, or heart failure.

**Figure 1:**
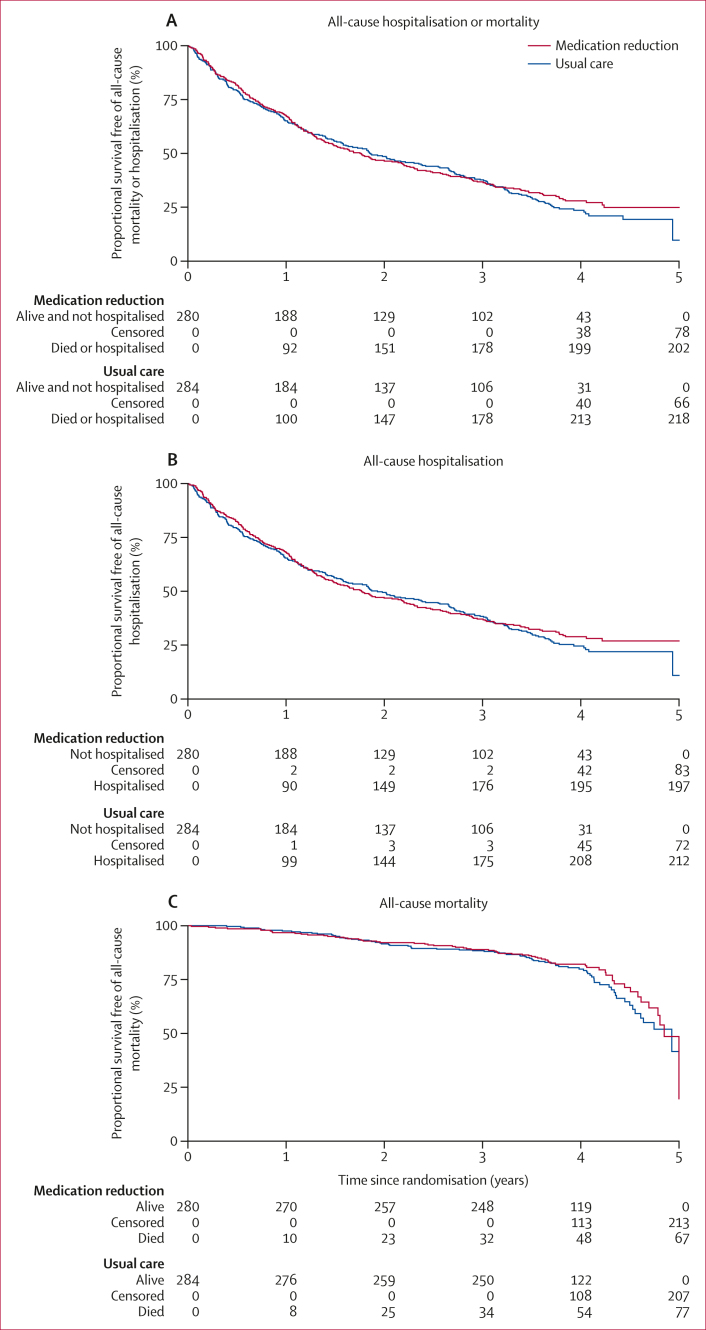
Survival curves showing the rates of all-cause hospitalisation or mortality (A), all-cause hospitalisation (B), and all-cause mortality (C)

The median period of follow-up for participants included in the primary analysis was 4·0 years (IQR 3·7–4·3; time at risk: 593 years [intervention] and 595 years [control]). Overall, 202 (72%) patients in the intervention group and 218 (77%) patients in the control group experienced all-cause hospitalisation or mortality during follow-up (adjusted hazard ratio [aHR] 0·93 [95% CI 0·76–1·12]; table 2, figure 1). There was some evidence that the proportion of participants experiencing the primary outcome in the per-protocol population was lower in the intervention group (aHR 0·80 [0·64–1·00]). Findings were similar when analysing the outcomes as binary count data (appendix p 131), and when limiting the analysis to those events that occurred before the COVID-19 pandemic (aHR for hospitalisation or mortality 1·02 [0·81–1·28]; appendix p 132). The incidence of events was similar across calendar years, despite the COVID-19 pandemic (appendix p 133). There was no evidence of an interaction between the effects of medication reduction on the primary outcome and participant sex (appendix p 132).

**Table 2:** Time-to-event analyses of clinical outcomes at follow-up

	Medication reduction group (n=280)	Usual care group (n=284)	Adjusted hazard ratio (95% CI)	p value
**Primary outcome (all-cause hospitalisation or mortality)**				
Intention-to-treat analysis	202 (72%) [593·0; 34·1]	218 (77%) [594·5; 36·7]	0·93 (0·76–1·12)∗	0·43
Per-protocol analysis†	126 (67%) [425·2; 29·6]	218 (77%) [594·5; 36·7]	0·80 (0·64–1·00)∗	0·053
**Secondary outcomes (intention-to-treat analysis)**				
All-cause hospitalisation	197 (70%) [593·0; 33·2]	212 (75%) [594·5; 35·7]	0·93 (0·76–1·13)∗	0·46
All-cause mortality	67 (24%) [1042·2; 6·43]	77 (27%) [1055·3; 7·30]	0·80 (0·57–1·12)∗	0·20
Emergency hospitalisation	123 (44%) [829·6; 14·83]	117 (41%) [869·6; 13·45]	1·09 (0·85–1·41)∗	0·50
Hospitalisation or death due to major cardiovascular events	52 (19%) [983·6; 5·29]	52 (18%) [1001·4; 5·19]	1·00 (0·68–1·46)‡	0·98
Hospitalisation or death due to myocardial infarction	14 (5%) [1021·8; 1·37]	16 (6%) [1038·1; 1·54]	0·86 (0·42–1·77)‡	0·69
Hospitalisation or death due to stroke	11 (4%) [1031·5; 1·07]	12 (4%) 1043·4; 1·15]	0·91 (0·40–2·06)‡	0·82
Diagnosis of dementia	14 (5%) [1012·1; 1·38]	12 (4%) [1030·3; 1·16]	1·16 (0·54–2·52)‡	0·71
Hospitalisation due to hypotension	21 (8%) [1017·7; 2·06]	15 (5%) [1040·0; 1·44]	1·37 (0·71–2·67)∗	0·35
Hospitalisation due to syncope	1 (<1%)	0 (0%)	··	··
Hospitalisation due to falls	0 (0%)	2 (<1%)	··	··
Hospitalisation due to fracture	2 (<1%) [1040·4; 0·19]	2 (<1%) [1053·2; 0·19]	1·15 (0·16–8·38)∗	0·89
Hospitalisation due to electrolyte abnormalities	31 (11%) [1009·6; 3·07]	30 (11%) [1023·8; 2·93]	1·02 (0·61–1·68)∗	0·95
Hospitalisation due to acute kidney injury	32 (11%) [1007·4; 3·18]	32 (11%) [1026·3; 3·12]	0·99 (0·60–1·62)∗	0·94

Data are n (%) [number of person-years at risk; incidence rate] unless otherwise stated.

∗ Cox proportional hazards model adjusting for baseline systolic blood pressure and intervention group as fixed effects, and including practice as a random effect. Hazard ratio <1 favours medication reduction group.

† 187 patients were included in the medication reduction group.

‡ Cox proportional hazards model adjusting for baseline systolic blood pressure and intervention group as fixed effects. Hazard ratio <1 favours medication reduction group.

There was no evidence of a difference between groups in any of the prespecified time-to-event secondary outcomes, including all-cause hospitalisation (figure 1); all-cause mortality (figure 1); emergency hospitalisation; and major cardiovascular events, stroke, myocardial infarction, or dementia (table 2). Few participants overall experienced hospitalisation with a fall or syncope event and there was no evidence of a difference between groups in the number of participants experiencing hospitalisation due to hypotension, fracture, acute kidney injury, or electrolyte abnormalities (table 2). Medication reduction was associated with a significant increase in primary care consultations related to hypertension in those participants who had a consultation (appendix p 134).

**Figure 2:**
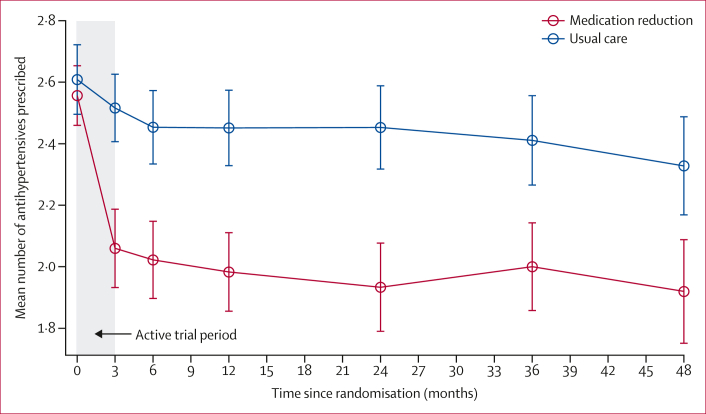
Antihypertensive medication prescription changes over time in participants registered to practices contributing to ORCHID (n=369) Error bars indicate 95% CIs.

Of the 282 participants assigned to medication reduction, 109 were taking fewer antihypertensives (51% of the 213 participants alive and not withdrawn) at follow-up compared with baseline (appendix p 135). This resulted in a larger reduction in overall antihypertensive prescription (compared with baseline) in the intervention group than the control group (table 3). Examining data from all participants in ORCHID for whom more granular data were available, this difference in antihypertensive treatment prescription was evident at 12-week follow-up and persisted throughout the period of long-term follow-up up to 4 years post-randomisation (figure 2). The results were similar when restricting the analysis to only those participants who were alive with at least 4 years of follow-up (appendix p 136).

**Table 3:** Medication prescriptions and blood pressure at 3-year follow-up

	Medication reduction group∗	Usual care group∗	Adjusted mean difference (95% CI)† or adjusted relative risk (95% CI)‡	p value
**Antihypertensive medications**				
Participants with available data	n=207	n=199	··	··
Number of antihypertensive medications at follow-up	1·9 (1·0)	2·3 (0·9)	··	··
Change from baseline	–0·6 (0·9)	–0·2 (0·8)	–0·35 (–0·52 to –0·18)†	0·0001
**All prescribed medications**				
Participants with available data	n=206	n=199	··	··
Number of all prescribed medications at follow-up	5·9 (2·9)	6·7 (3·3)	··	··
Change from baseline	0·9 (3·3)	2·0 (3·8)	–0·94 (–1·50 to –0·37)†	0·0011
**Systolic blood pressure**				
Participants with available data	n=188	n=191	··	··
Systolic blood pressure at follow-up, mm Hg	139·2 (16·8)	138·8 (18·0)	··	··
Change from baseline, mm Hg§	9·8 (19·6)	8·0 (20·6)	0·71 (–2·76 to 4·18)†	0·69
**Diastolic blood pressure**				
Participants with available data	n=188	n=191	··	··
Diastolic blood pressure at follow-up, mm Hg	72·2 (9·8)	73·9 (11·8)	··	··
Change from baseline, mm Hg§	3·9 (10·8)	4·0 (13·2)	–0·48 (–2·81 to 1·86)†	0·69
**Blood pressure control**				
Participants with available data	n=188	n=191	··	··
Systolic blood pressure control	145 (77%)	151 (79%)	0·97 (0·87 to 1·08)‡	0·57
Diastolic blood pressure control	178 (95%)	178 (93%)	1·02 (0·96 to 1·07)‡	0·56

Data are mean (SD) or n (%).

∗ Only participants with blood pressure readings between 2·5 years and 3·5 years of follow-up were included in the analysis; the numbers of participants with available data during this time are shown.

† Adjusted mean difference: calculated using a mixed effect model adjusted for baseline systolic blood pressure, including practice as a random effect. Adjusted mean difference <0 favours medication reduction group.

‡ Adjusted relative risk: calculated using a generalised linear mixed model with Poisson family and log link and robust variance estimates, adjusting for baseline systolic blood pressure, including practice as a random effect. Adjusted relative risk >1 favours medication reduction group.

§ Positive number indicates that blood pressure has increased from baseline.

There was no evidence of a difference in systolic or diastolic blood pressure at 3-year follow-up (table 3). Similarly, there was no evidence of a difference in mean recorded blood pressure at the 3-year follow-up (table 3), although it was higher than baseline in both randomised groups, perhaps reflecting the different methods of measurement (research standard at baseline, routine measurement at follow-up). Examining data from participants in ORCHID, the small but significant difference in systolic blood pressure observed during the original trial (adjusted mean difference 3·3 mm Hg [95% CI 0·3–6·4]) persisted for 6 months, before disappearing thereafter (appendix pp 137–138), and results were similar when restricting the analysis to only those participants who were alive with at least 4 years of follow-up (appendix p 139).

There was no evidence of any interaction effects between the randomised group and prespecified subgroups in rates of hospitalisation or death, systolic blood pressure control, or change in blood pressure by subgroups (appendix pp 140–142).

## Discussion

This study evaluated long-term follow-up data from a randomised controlled trial using routine electronic health records to examine the effects of antihypertensive deprescribing on hospitalisation and all-cause mortality in older patients with controlled systolic blood pressure. After 4 years of follow-up, antihypertensive deprescribing was sustained in over half of those attempting it in the intervention group, with no evidence of harm in terms of hospitalisation or mortality. Although systolic blood pressure was initially raised after medication reduction, this difference from usual care disappeared after 6 months and there was no evidence of a difference between groups at the end of follow-up. There was no evidence of benefit (or harm) from deprescribing antihypertensives for the secondary outcomes examined, but these findings do suggest that deprescribing an antihypertensive medication might be safe in older patients living in the community with controlled systolic blood pressure on two or more blood pressure-lowering medications.

To our knowledge, this is the largest trial of antihypertensive deprescribing conducted to date,^23^ with longer follow-up than any previous deprescribing intervention trial.^24^, ^25^, ^26^, ^27^ Using data from electronic health records, it was possible to follow up over 99% of randomly assigned participants, collecting detailed information from both primary and secondary care settings and from civil registration death records. Unlike other long-term trial follow-up studies,^28^^,^^29^ differences in treatment prescription between the intervention and control groups were sustained throughout follow-up. Moreover, to our knowledge, this is the first study to examine the effect of antihypertensive deprescribing on hospitalisation and mortality.^12^ To date, very few studies examining the effects of antihypertensive deprescribing in older people have been undertaken. The recent ATEMPT trial^30^ aimed to compare a strategy of antihypertensive treatment intensification with a strategy of antihypertensive deprescribing, using a decentralised model of intervention delivery. This smaller trial with 13 months of follow-up did not achieve any medication reduction in the deprescribing group (participants were taking on average 0·4 more antihypertensives at follow-up); this was attributed to a lack of participant willingness to reduce medications and concerns about negative consequences expressed by participants assigned to the deprescribing group. In the present trial, which enrolled participants who were older and more frail than those enrolled in ATEMPT, we did not encounter such issues, with deprescribing achieved in 100% of participants assigned to the intervention, which was maintained for 4 years in over half of these participants. The success of the OPTiMISE intervention might be in part attributed to the fact that it was delivered by primary care physicians, who are known and trusted by their patients and are therefore better placed to deliver such an intervention.

The aforementioned Cochrane review, which found just six trials of antihypertensive deprescribing in patients aged 50 years or older (including 1073 participants) with a maximum follow-up of 56 weeks, found no evidence of an effect of antihypertensive deprescribing on clinical outcomes.^12^ However, very few of the trials included in this review reported outcome events of interest, and therefore it was underpowered to show any associations with hospitalisation (19 events), mortality (18 events), or major cardiovascular events (three events). In contrast, the present study enrolled 569 participants aged 80 years or older and followed them up for 4 years (including 1188 participant-years of follow-up). As a result, substantially more outcome events were captured, including 409 hospitalisations, 144 deaths, and 104 major cardiovascular events, enabling the effects of deprescribing antihypertensive treatment on clinical outcomes to be estimated with much greater precision than previously possible.

Although this is the first antihypertensive deprescribing trial conducted in older adults to examine major clinical endpoints, there are previous trials that have explored the effect of antihypertensive prescribing on similar outcomes. Most notably, the Hypertension in the Very Elderly Trial (HYVET)^3^ and Systolic Blood Pressure Intervention Trial (SPRINT)^2^ both examined the effects of blood pressure lowering in older adults and showed significant reductions in major cardiovascular events and all-cause mortality. These findings appear to be in contrast with the present study, in which more than half of participants in the intervention group were taking fewer antihypertensives at follow-up compared with baseline but there was no evidence of a difference in blood pressure, mortality, or major cardiovascular events. This might be partly explained by different inclusion criteria used for each of these studies, which resulted in different sample populations.^22^ Participants in HYVET and SPRINT typically had uncontrolled blood pressure at baseline, with a mean systolic blood pressure of 173 mm Hg and 142 mm Hg, respectively.^2^^,^^3^ By contrast, those in OPTiMISE had well controlled blood pressure (mean blood pressure at baseline was 130 mm Hg) and, on average, had been diagnosed with hypertension for more than 15 years. These differences are reflected in the higher rates of serious adverse events in the OPTiMISE study.^2^^,^^3^ Participants in OPTiMISE were more likely to die (687 [OPTiMISE] *vs* 535 [HYVET] *vs* 220 [SPRINT] deaths per 10 000 participant-years of follow-up)^2^^,^^3^ and experience major cardiovascular events during follow-up (524 [OPTiMISE] *vs* 425 [HYVET] *vs* 325 [SPRINT] events per 10 000 participant-years of follow-up).^2^^,^^3^ This suggests that participants in OPTiMISE were less healthy and had less to gain from continued therapy, due to the risk of competing (ie, non-cardiovascular) events.

There are no previous trials examining the effect of antihypertensive treatment over many years, and so how the body adapts to long periods of sustained blood pressure lowering remains unknown. One possible explanation for the observed findings in this study could be that long-term effective antihypertensive treatment reverses the structural changes of hypertension and results in prolonged blood pressure normality, even after medication reduction.^31^^,^^32^

This study suggests that deprescribing antihypertensives in patients with well controlled blood pressure who have been taking treatment for many years might be safe, and therefore supports new clinical guidelines^8^ recommending that reduction of therapy be considered in older patients with low systolic blood pressure (<120 mm Hg) and high frailty and might even support a relaxation of that threshold.

This trial found that medication reduction was associated with a significant increase in primary care consultations, probably due to the additional safety visit at 4 weeks in the intervention group during which blood pressure was checked. This increased workload, albeit small, warrants consideration about whether this intervention should be adopted in routine clinical practice. The trial also found no evidence of a benefit from antihypertensive deprescribing in terms of reduction in serious adverse events associated with antihypertensive treatment prescription, although these events were typically rare. This was also the case for outcomes such as incident dementia, which is of particular concern in this population, with some evidence now suggesting that continued lowering of blood pressure can lower the risk of cognitive decline in older adults.^33^ As a result, without larger trials or meta-analyses of multiple deprescribing trials, it might be difficult to detect any potential benefits or harms from this type of intervention. Because this study used routinely collected data for follow-up, it was not possible to examine other potential effects of deprescribing, captured through participant reporting, such as a reduction in side-effects (eg, ankle swelling, cough, or fatigue) or overall medication burden, and these outcomes should be explored in future studies. In this population, living well and dying well are important^34^ and therefore should be considered as outcomes in future studies.^35^

The study also has some limitations. Firstly, cause-specific outcomes were based on codes from electronic health record data (and therefore the judgement of the coding clinician), and no independent adjudication committee was set up to review these outcome data. It is therefore possible that some events could have been misclassified, which could have affected the overall number of cause-specific events captured in this study, although any such misclassification would be expected in both treatment groups so would be unlikely to have had an impact on deprescribing effect estimates.^36^

Secondly, blood pressure measurements captured at follow-up were based on measurements taken as part of routine clinical practice. These are typically higher than those taken using an automated office blood pressure monitor as part of a research study.^37^ This was evident in the present study, where trial baseline systolic blood pressure measurements taken by a research nurse were 5 mm Hg lower than pre-baseline readings taken from the routine electronic health record, and 9 mm Hg lower than subsequent follow-up readings 12–48 months after randomisation, although the latter might also include an element of regression to the mean.

Thirdly, although included participants were older adults, with complex multiple long-term conditions and moderate frailty, participants who lacked capacity to give informed consent (including those with dementia) were excluded from the original trial at the request of the regulatory body approving this research (the MHRA) due to the absence of previous data on the safety of deprescribing antihypertensives in this population. This exclusion could affect the generalisability of the study findings and therefore caution should be exercised when applying these results in practice to individuals with substantial cognitive impairment.

Finally, although the majority of participants experienced events during follow-up, the trial was not designed to detect differences between groups in any clinical outcome, nor was it powered to determine non-inferiority. For rarer outcomes, such as stroke and serious falls, larger studies are required to determine the effects of antihypertensive deprescribing.

In summary, this long-term follow-up of the OPTiMISE trial shows that medication reduction can be sustained in over half of those attempting it for approximately 4 years, with no evidence of harm in terms of hospitalisation or all-cause mortality. These findings suggest that deprescribing an antihypertensive medication might be safe, and could be attempted to reduce polypharmacy in older patients living with complex multiple long-term conditions in the community with controlled blood pressure, prescribed two or more antihypertensive medications.

## Data sharing

Requests for sharing of de-identified individual participant data and a data dictionary defining each field in the set will be considered by the corresponding author. The study protocol is available in the supplementary appendix and on our trial website (https://www.phctrials.ox.ac.uk/studies/optimise).

## Declaration of interests

JPS reports funding from the Wellcome Trust/Royal Society, National Institute for Health and Care Research (NIHR), British Heart Foundation, and Stroke Association; payment for consultancy from DoctorLink; is a Data Safety Monitoring Board member for the Hypertension Treatment in Nigeria Study sponsored by Northwestern University; and is Secretary and trustee for the British and Irish Hypertension Society. GAF is Board non-executive Director for the National Institute for Health and Care Excellence. FDRH reports honoraria for lectures from Pfizer/BMS, Bayer, Boehringer Ingelheim, and AstraZeneca; and is chair of the European Primary Care Cardiovascular Society and International Primary Care Cardiovascular Society. SdL has received research grants to his university for investigator-led cardiometabolic research from Bristol Myers Squibb, Eli Lilly, GSK, MSD, Novo Nordisk, Pfizer, Sanofi, and Seqirus; has been a member of advisory boards for AstraZeneca, GSK, Sanofi, and Seqirus; and has received medical writing support provided by AstraZeneca, GSK, and Pfizer. JM reports funding from an NIHR Senior Investigator Award and honoraria for lectures from Bristol Myers Squibb and Pfizer. RAP reports NIHR funding to his institution for other research related to medicines use and Medical Research Council funding to his institution unrelated to this project. L-MY reports NIHR funding to her institution for other research. RJM reports funding from the NIHR, British Heart Foundation, and Stroke Association; has received royalties, consulting fees (paid to his institution), and research equipment from Omron; has received royalties from Sensyne; and has received honoraria (paid to his institution) for lectures from the Finnish Hypertension Society and Canadian Hypertension Society.
