# Supplementary material for: Effect of antihypertensive deprescribing on hospitalisation and mortality: long-term follow-up of the OPTiMISE randomised controlled trial
Source: Lancet Healthy Longev. 2024 Aug;5(8):e563–73. doi: 10.1016/S2666-7568(24)00131-4 (PMC11327766; doi:10.1016/S2666-7568(24)00131-4)
Supplement: Supplementary appendix [file mmc1.pdf]

THE LANCET
Healthy Longevity

Supplementary appendix

This appendix formed part of the original submission and has been peer reviewed.
We post it as supplied by the authors.

Supplement to: Sheppard JP, Temple E, Wang A, et al. Effect of antihypertensive deprescribing on hospitalisation and mortality: long-term follow-up of the OPTiMISE randomised controlled trial. *Lancet Healthy Longev* 2024. https://doi.org/10.1016/S2666-7568(24)00131-4

Supplementary appendix

Contents

**Appendix 1.** OPTiMISE Investigators	2
**Appendix 2.** OPTiMISE Trial Protocol V6.0 17.04.2023	4
**Appendix 3.** OPTiMISE Trial Extension Statistical Analysis Plan V1.0 27.09.2023	67
**Table S1.** Inclusion and exclusion criteria for the trial	129
**Figure S1.** Flow of participants through the study	130
**Table S2.** Binary analyses of clinical outcomes at follow-up (intention to treat analyses)	131
**Table S3.** Post-hoc sensitivity analyses of outcomes occurring prior to the first UK lockdown for the Coronavirus Pandemic on 23rd March 2020 and the primary outcome according to participant sex	132
**Figure S2.** Rates of all-cause hospitalisation or death by calendar time	133
**Table S4.** Primary care consultations related to hypertension during follow-up	134
**Table S5.** Maintenance of medication prescription strategies during follow-up	135
**Figure S3.** Antihypertensive medication prescription changes over time, in participants registered to practices contributing to the ORCHID who were alive at least 4 years of follow-up (n=278).	136
**Table S6.** Blood pressure prior to baseline, at baseline and during follow-up, in participants registered to practices contributing to the ORCHID (n=369).	137
**Figure S4.** Blood pressure changes over time, in participants registered to practices contributing to the ORCHID (n=369). Error bars indicate 95% confidence intervals.	138
**Figure S5.** Blood pressure changes over time, in participants registered to practices contributing to the ORCHID who were alive for at least 4 years of follow-up (n=278).	139
**Figure S6.** Hazard ratio of time to all-cause hospitalisation or death by prespecified subgroups	140
**Figure S7.** Relative risk of systolic blood pressure control at 3-year follow-up by prespecified subgroups	141
**Figure S8.** Mean difference in change in systolic blood pressure (mm Hg) at 3-year follow-up by prespecified subgroups	142

Appendix 1. OPTIMISE Investigators

Coordinating centre (Oxford Primary Care Clinical Trials Unit)
Alecia Nickless, Christopher Lovekin, David Judge, David Watt, Hannah Ashby, Hannah Swayze, Lazarina Engonidou, Sadie Kelly, Bethany Dimmet (Cambridge), Sarah Oliver (Southampton).

Participating National Institute for Health Research Clinical Research Networks and staff
Thames Valley and South Midlands
Debbie Kelly, Lydia Owen, Diane Lonsdale, Claire Winch, Sarah Wytrykowski, Katherine Priddis
Wessex
Kerry Gunner, Clare Grocutt, Julie Kennedy, Vivian Sparshott
Eastern
Clare Fletcher, Jenny Johnson, Kirsti Withington, Marie Corcoran
West Midlands
Claire Brown, Elaine Butcher, Eleanor Hoverd, Pauline Darbyshire, Sarah Joshi, Susan Zhao, Tracey Davenport, Andrea Isaew, Julie Timmins, Azaria Ballintine, Somi Spannuth
West of England
Amy Herbert, Bethan Rees, Rwth Leach, Rose Hawkins, Emma Jennings, Lara Peniket, Hanorah Saliba

Participating practices and general practitioners
Ascot Medical Centre (Dr Edward Williams), Berinsfield Health Centre (Dr Jonathan Crawshaw), Bicester Health Centre (Dr Robin Fox), The Boathouse Surgery (Dr Thomas Morgan), Bampton Medical Practice (Dr Peter Grimwade), 27 Beaumont Street Medical Practice (Prof Richard McManus), Botley Medical Centre (Dr Mary Akinola), Broadshires Health Centre (Dr Christine A'Court), The Cedars Surgery (Dr Clare Nieland), Cookham Medical Centre (Dr Kenney Tsoi), Church Street Practice (Dr Matthew Gaw), Eynsham Medical Group (Dr Ian Binnian), Hughenden Valley Surgery (Dr Lynette Hykin), Hollow Way Medical Practice (Dr Debbie Goldman), Jericho Health Centre (Dr Mark O'Shea), King Edward Street Medical Practice (Dr Brian Nicholson), Magnolia House Surgery (Dr Kavil Patel), Medicas Health Limited (Dr Ruth Mason), Milman Road Surgery (Dr Aneeka Bajwa), St Clements Surgery (Dr Hernandez), South Oxford Health Centre (Dr Nick Wooding), Summertown Health Centre (Dr Kyle Knox), Temple Cowley Medical Group (Dr Elisabetta Angeleri-Rand), Chalgrove and Watlington Surgeries (Dr Grace Ding), The Downland Practice (Dr Chloe Evans), The Ivers Practice (Dr Neetul Shah), Thatcham Medical Practice (Dr Sarah Wadsworth), Woodlands Medical Centre (Dr Adam Jones), Wokingham Medical Centre (Dr Zishan Ali), Whaddon Medical Centre (Dr Asim Malik), White Horse Medical Practice (Dr Simon Cartwright), Homewell and Curlew Practice (Dr Helen Whiting), Liphook and Liss Surgery (Dr Anna Lalonde), Vine Medical Group (Dr Olivia Boocock), Wareham Surgery (Dr James Bennett), Badgerswood and Forest Surgery (Dr Helen Sherrell), Lordshill Health Centre (Dr Faycal Elhani), Highcliffe Medical Centre (Dr Zelda Cheng), Cowplain Family Practice (Dr Nicola Millen), Blackthorn Health Centre (Dr Ali Shahsavanpour), Feltwell Surgery (Dr Michael Pullen), Bridge Street Surgery (Dr Clare Hambling), Little St John Street (Dr Gary Taylor), Hoveton and Wroxham Medical Practice (Dr Carsten Dernedde), Wansford and Kings Cliffe Practice (Dr Amrit Takhar), Woolpit Health Centre (Dr Richard West), Shelford Medical Practice (Dr Chris Schramm), St Mary's Surgery (Dr Katrina Young), Bennfield Surgery (Dr Nick Doherty and Dr Suparna Behura), West Heath Primary Care Centre (Dr Gulshan Arora), Westside Medical Centre (Dr Mark Lindsey), Eve Hill Medical Practice (Dr David Shukla), River Brook Medical Centre, (Dr Naresh Chauhan),

Park Leys Medical Practice (Dr Rachel Spencer), Manor Court Surgery (Dr Farhana Lockhat), The Marches Surgery (Dr Crispin Fisher), Winyates Health Centre (Dr Vattakkatt Premchand), Avonside Health Centre (Dr Sarah Colliver), Hastings House Surgery (Dr Paul de Cates), Maypole Health Centre (Dr Soong Loy Yap), Eden Court Medical Practice (Dr Naresh Aggarwal), Old Priory Surgery (Dr Mark Grocutt), Courtside Surgery (Dr Vicky Smith), Trowbridge Health Centre (Dr Toby Cookson), Phoenix Surgery (Dr Naomi Vernon), West Walk Surgery (Dr Sam Davies), Winchcombe Medical Centre (Dr Richard Tribley), Mann Cottage Surgery (Dr Cathy Bobrow), Chipping Campden Surgery (Dr Rebecca Zamir)

Trial Steering Committee

Prof Tom Robinson (Chair, University of Leicester), Prof Rod Taylor (University of Exeter), Prof Richard Lindley (University of Sydney), Prof Peter Bower (University of Manchester), Ms Margaret Ogden (Patient and public involvement representative), Ms Anita Higham (Patient and public involvement representative)

Data Monitoring Committee

Prof John Gladman (Chair, University of Nottingham), Prof Una Martin (University of Birmingham), Dr Martyn Lewis (Keele University)

Appendix 2. OPTiMISE Trial Protocol V6.0 17.04.2023

OPTiMISE

Optimising Treatment for Mild Systolic Hypertension in the Elderly

STUDY PROTOCOL

V6.0_17Apr2023

UNIVERSITY OF OXFORD
NUFFIELD DEPARTMENT OF PRIMARY CARE HEALTH SCIENCES

UNIVERSITY OF CAMBRIDGE
Public Health and Primary Care
The Primary Care Unit

UNIVERSITY OF Southampton

NHS
National Institute for Health Research

Trial Title: OPtimising Treatment for MIld Systolic hypertension in the Elderly: a randomised controlled trial

Internal Reference Number / Short title: OPTiMISE

Ethics Ref: 16/SC/0628

EudraCT Number: 2016-004236-38

Date and Version No: V6.0_17Apr2023

**Chief Investigator:**	**Professor Richard McManus**<br>Nuffield Department of Primary Care Health Sciences<br>University of Oxford<br>Radcliffe Primary Care<br>Radcliffe Observatory Quarter<br>Woodstock Road<br>Oxford OX2 6GG<br>Tel: +44 (0)1865 617834<br>Fax: +44 (0)1865 289287<br>Email: richard.mcmanus@phc.ox.ac.uk
**Lead Scientific Investigator:**	**Dr James Sheppard** (University of Oxford)
**Co-Investigators:**	Prof Richard Hobbs, Prof Carl Heneghan, Dr Emma Ogburn, Dr Ly-Mee Yu, Prof Gary Ford (University of Oxford)<br>Dr Jenni Burt, Prof Jonathan Mant, Dr John Benson **(**University of Cambridge)<br>Dr Mark Lown, Prof Paul Little (University of Southampton)<br>Dr Rupert Payne (University of Bristol, Bristol)<br>Marney Williams, Lay patient advisor.
**Sponsor:**	Research Governance, Ethics & Assurance Team (RGEA),<br>University of Oxford,<br>Boundary Brook House,<br>Churchill Drive,<br>Headington,<br>Oxford,<br>OX3 7GB

**Funder:**	National Institute for Health Research (NIHR), Oxford Collaborations for Leadership in Applied Health Research and Care (CLARHC) and School for Primary Care Research (SPCR)

The British Heart Foundation

Chief Investigator Statement: I have read this protocol and agree to abide by all provisions set forth therein. I agree to comply with the International Conference on Harmonisation Tripartite Guideline on Good Clinical Practice.

Chief Investigator Signature:

Statistician Signature:

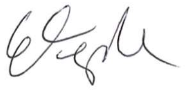

No potential conflict of interest

Confidentiality Statement

This document contains confidential information that must not be disclosed to anyone other than the Sponsor, the Investigator Team, host organisation, and members of the Research Ethics Committee, unless authorised to do so.

TABLE OF CONTENTS

1. KEY TRIAL CONTACTS 10
2. SYNOPSIS 11
3. ABBREVIATIONS 14
4. BACKGROUND AND RATIONALE 16
5. OBJECTIVES AND OUTCOME MEASURES 19
6. TRIAL DESIGN 22
7. PARTICIPANT IDENTIFICATION 22
7.1. Trial Participants 22
7.2. Inclusion Criteria 23
7.3. Exclusion Criteria 23
8. TRIAL PROCEDURES 24
8.1. Recruitment 24
8.1.1. Practice and GP Recruitment 24
8.1.2. Practice database searches 24
8.1.3. Patient Recruitment 24
8.2. Informed Consent 25
8.3. Screening and Eligibility Assessment 26
8.4. Baseline Assessments 26
8.5. Randomisation, blinding and code-breaking 27
8.6. Subsequent visits 28
8.7. Internal feasibility study 28
8.7.1. Feasibility phase 1 28
8.7.2. Feasibility phase 2 29
8.8. Discontinuation/Withdrawal of Participants from Trial Treatment 29
8.9. Definition of End of Trial 29
9. INVESTIGATIONAL MEDICINAL PRODUCT (IMP) 29
9.1. Intervention group (IMP Description) 29
9.2. Control group 31
9.3. Compliance with Trial Treatment 31
9.4. Concomitant Medication 31

9.5. Post-trial Treatment 31
10. QUALITATIVE SUB STUDIES 32
10.1. Qualitative study 1: interviews with doctors and patient 32
10.1.1. Participant identification and recruitment 32
10.1.2. Informed consent 32
10.1.3. Interview approach 33
10.1.4. Data analysis 33
10.2. Qualitative study 2: assessment of trial recruitment and data collection procedures 33
10.3. Integration of qualitative sub-studies with trial procedures 34
11. ECONOMIC SUB STUDY 34
12. SAFETY REPORTING 34
12.1. Definitions 34
12.2. Causality 36
12.3. Trial specific issues around patient safety 36
12.3.1. Risks of treatment/medication reduction 36
12.3.2. Trial follow-up 36
12.3.3. Measures to minimise the risks associated with medication reduction 36
12.4. Recording Procedures for Adverse Events 37
12.5. Reporting Procedures for Serious Adverse Events 37
12.6. Reporting Procedures for SUSAR 38
12.7. Data Monitoring and Ethics Committee 39
12.8. Development Safety Update Reports 39
13. STATISTICS 39
13.1. Description of Statistical Methods 39
13.1.1. Long term follow-up 40
13.2. The Number of Participants 41
13.3. The Level of Statistical Significance 42
13.4. Criteria for the Termination of the Trial 42
13.5. Procedure for Accounting for Missing, Unused, and Spurious Data. 42
13.6. Inclusion in Analysis 42
13.7. Procedures for Reporting any Deviation(s) from the Original Statistical Plan 42
14. DATA MANAGEMENT 42

14.1. Source Data 42
14.2. Access to Data 43
14.3. Data Handling and Record Keeping 43
15. QUALITY ASSURANCE PROCEDURES 43
16. SERIOUS BREACHES 44
17. ETHICAL AND REGULATORY CONSIDERATIONS 44
17.1. Declaration of Helsinki 44
17.2. Guidelines for Good Clinical Practice 44
17.3. Approvals 44
17.4. Reporting 45
17.5. Participant Confidentiality 45
17.6. Expenses and Benefits 45
17.7. Other Ethical Considerations 45
18. FINANCE AND INSURANCE 45
18.1. Funding 46
18.2. Insurance 46
19. PUBLICATION POLICY 46
20. REFERENCES 47
21. APPENDIX A: TRIAL FLOW CHART 52
22. APPENDIX B: DATA COLLECTION SOURCES AND SCHEDULE 53
23. APPENDIX C: ITEMS INCLUDED IN FRAILTY INDEX ASSESSMENT 54
24. APPENDIX D: MEDICATION REDUCTION ALGORITHM 57
25. APPENDIX E: POST MEDICATION REDUCTION MONITORING FLOW CHART 58
26. APPENDIX F: SELF-MONITORING PROTOCOL (TRAFFIC LIGHT SYSTEM) 59
27. APPENDIX G: AMENDMENT HISTORY 60
28. APPENDIX H: LONG TERM FOLLOW-UP 62

1. KEY TRIAL CONTACTS

**Chief Investigator**	Professor Richard McManus<br>**Address:** Nuffield Department of Primary Care Health Sciences, University of Oxford, Radcliffe Primary Care, Radcliffe Observatory Quarter, Woodstock Road, Oxford, OX2 6GG<br>**Email:** richard.mcmanus@phc.ox.ac.uk<br>**Telephone:** +44 (0)1865 617834<br>**Fax:** +44 (0)1865 289287
**Sponsor**	Ms Heather House<br>**Address:** Research Governance, Ethics & Assurance Team (RGEA), University of Oxford, Boundary Brook House, Churchill Drive, Headington, Oxford, OX3 7GB<br>**Email:** RGEA.Sponsor@admin.ox.ac.uk<br>**Telephone:** n/a<br>**Fax:** n/a
**Clinical Trials Unit/ Statistician**	Dr Ly-Mee Yu<br>**Address:** Primary Care Clinical Trials Unit, Nuffield Department of Primary Care Health Sciences, University of Oxford, Radcliffe Primary Care, Radcliffe Observatory Quarter, Woodstock Road, Oxford, OX2 6GG<br>**Email:** ly-mee.yu@phc.ox.ac.uk<br>**Telephone:** +44 (0)1865 617199<br>**Fax:** +44 (0)1865 289287
**Statistician**	Eleanor Temple<br>**Address:** Primary Care Clinical Trials Unit, Nuffield Department of Primary Care Health Sciences, University of Oxford, Radcliffe Primary Care, Radcliffe Observatory Quarter, Woodstock Road, Oxford, OX2 6GG<br>**Email:** eleanor.temple@phc.ox.ac.uk<br>**Telephone:** n/a<br>**Fax:** n/a

2. SYNOPSIS

**Trial Title**	OPtimising Treatment for MIld Systolic hypertension in the Elderly: a randomised controlled trial	
**Internal ref. no. (or short title)**	OPTiMISE	
**Clinical Phase**	Phase IV trial	
**Trial Design**	Primary Care based, open label, randomised controlled trial with embedded qualitative components	
**Trial Participants**	Patients aged ≥80 years, with controlled blood pressure (systolic blood pressure <150mmHg) receiving ≥2 antihypertensive medications, with no compelling indication for medication continuation and whom the GP considers could benefit from medication reduction due to existing polypharmacy, co-morbidity and/or frailty.	
**Planned Sample Size**	540 (plus any patients who are booked in for a consent visit once 540 participants have been randomised)	
**Qualitative sub-studies: participants**	Interviews: 15 GPs and 15 patients potentially eligible for the trial<br>Recording of recruitment appointments: 75 patients potentially eligible for the trial	
**Treatment duration**	12 weeks	
**Follow up duration**	12 weeks – see Appendix H for long-term outcomes	
**Planned Trial Period**	01/01/2017 - 31/12/2024	
	**Objectives**	**Outcome Measures**
**Primary**	To determine if a reduction in medication can achieve a proportion of patients with clinically safe levels (defined as a systolic blood pressure <150mmHg) which is non-inferior (within 10%) to that achieved by the usual care group.	The proportion of patients with controlled systolic blood pressure levels (systolic blood pressure <150mmHg) at 12 week follow-up.
**Secondary**	Determine the proportion of patients in intervention arm who maintain medication reduction through to follow-up (*i.e.* are *not* restarted on therapy)	Proportion of patients randomized to the intervention arm who maintain medication reduction throughout 12 week follow-up.
	Determine the difference in quality of life (according to EQ-5D-5L) between groups at 12 week follow-up.	EQ-5D-5L score at 12 week follow-up.
	Determine the difference in frailty (according to the FRAIL scale/frailty index) between the two groups at 12 week follow-up.	FRAIL scale score/frailty index at 12 week follow-up.
	Determine the difference in the change in mean clinic systolic blood pressure (from baseline) between the two groups at 12 week follow-up.	Change in mean clinic systolic blood pressure (from baseline) at 12 week follow-up.

	Determine the difference in the change in mean clinic diastolic blood pressure (from baseline) between the two groups at 12 week follow-up.	Change in mean clinic diastolic blood pressure (from baseline) at 12 week follow-up.
	Determine the difference in reported potential side effects to medication between the two groups at 12 week follow-up (e.g. coughs, dizziness, syncope, ankle swelling, etc.).	The proportion of patients reporting potential side effects to medication (e.g. coughs, dizziness, syncope, ankle swelling, etc.).
	Determine the difference in routinely reported serious adverse events between the two groups at 12 week follow-up (hospitalisation due to falls, myocardial infarction, stroke or all-cause mortality).	The proportion of patients reporting adverse events (hospitalisation due to serious falls, myocardial infarction, stroke or all-cause mortality).
	Determine the characteristics (e.g. age, gender, ethnicity, medical history) of the baseline screening and sample population and examine how these relate to individuals eligible/not eligible for recent blood pressure lowering trials conducted in the elderly.[1-3]	• Descriptive statistics of the screening and baseline population<br>• Comparison of these characteristics with those eligible/not eligible for recent blood pressure lowering trials conducted in the elderly
**Exploratory analyses**	Subgroup analyses of blood pressure control, change in blood pressure and maintenance of medication reduction, by different levels of baseline frailty	The following outcomes, stratified by baseline frailty (frailty index score):<br>• The proportion of patients with controlled systolic blood pressure levels (systolic blood pressure <150mmHg) at 12 week follow-up<br>• Change in mean clinic systolic blood pressure (from baseline) at 12 week follow-up<br>• Proportion of patients who maintain medication reduction throughout follow-up
	Subgroup analyses of blood pressure control, change in blood pressure and maintenance of medication reduction, by different levels of baseline functional independence	The following outcomes, stratified by baseline functional independence (modified Rankin Scale):<br>• The proportion of patients with controlled systolic blood pressure levels (systolic blood pressure <150mmHg) at 12 week follow-up

		• Change in mean clinic systolic blood pressure (from baseline) at 12 week follow-up<br>• Proportion of patients who maintain medication reduction throughout follow-up
	Subgroup analyses of blood pressure control, change in blood pressure and maintenance of medication reduction, by different levels of baseline cognitive function	The following outcomes, stratified by baseline cognitive function (MOCA score):<br>• The proportion of patients with controlled systolic blood pressure levels (systolic blood pressure <150mmHg) at 12 week follow-up<br>• Change in mean clinic systolic blood pressure (from baseline) at 12 week follow-up<br>• Proportion of patients who maintain medication reduction throughout follow-up
	Subgroup analyses of blood pressure control, change in blood pressure and maintenance of medication reduction, by number of antihypertensive medications prescribed at baseline	The following outcomes, stratified by number of antihypertensive medications prescribed at baseline:<br>• The proportion of patients with controlled systolic blood pressure levels (systolic blood pressure <150mmHg) at 12 week follow-up<br>• Change in mean clinic systolic blood pressure (from baseline) at 12 week follow-up<br>• Proportion of patients who maintain medication reduction throughout follow-up
	Subgroup analyses of blood pressure control, change in blood pressure and maintenance of medication reduction, by number of co-morbidities at baseline	The following outcomes, stratified by number of co-morbidities at baseline:<br>• The proportion of patients with controlled systolic blood pressure levels (systolic blood pressure <150mmHg) at 12 week follow-up<br>• Change in mean clinic systolic blood pressure (from baseline) at 12 week follow-up<br>• Proportion of patients who maintain medication reduction throughout follow-up

Qualitative sub study 1: primary outcome	Determine the barriers and facilitators for patients and GPs to reducing antihypertensive medication to inform both the ongoing trial and potential future implementation.	• Thematic analysis of chart-stimulated interviews with GPs<br>• Thematic analysis of 'Brown bag' medication review interviews with patients
Qualitative sub-study 2: primary outcome	Determine how trial recruitment is discussed and understood by recruiters and patients.	• Thematic analysis of audio-recorded recruitment appointments
Economic sub study primary outcome	Determine the cost-effectiveness of the intervention in terms of cardiovascular, quality of life and cost outcomes.	Cardiovascular disease risk, costs and quality-adjusted-life years.
**Investigational Medicinal Product(s)**	Medication reduction - one antihypertensive medication stopped in line with GP and patient preference and existing guidelines, where appropriate (See medication reduction algorithm in Appendix C).	
**Formulation, Dose, Route of Administration**	At the discretion of the consulting GP, based on indications, co-morbidities, blood pressure and guidance from the study team.	

3. ABBREVIATIONS

AE	Adverse event
AR	Adverse reaction
BP	Blood pressure
CLAHRC	Collaborations for Leadership in Applied Health Research and Care
CRN	Clinical Research Network
CTA	Clinical Trials Authorisation
CVD	Cardiovascular Disease
DMEC	Data Monitoring and Ethics Committee
eCRFs	Electronic Case Report Form
eFI	Electronic frailty index
EudraCT	European Clinical Trials Register
GCP	Good Clinical Practice
GP	General Practitioner
HYVET	HYpertension in the Very Elderly Trial
ICH	International Conference on Harmonisation
IMP	Investigational Medicinal Product
ISRCTN	International Standard Randomised Controlled Trial Number

ITT	Intention-to-treat analysis
MHRA	Medicines and Healthcare products Regulatory Agency
MI	Myocardial Infarction
NICE	National Institute for Health and Care Excellence
NIHR	National Institute for Health Research
NHS	National Health Service
OPTiMISE	OPtimising Treatment for MIld Systolic hypertension in the Elderly: a randomised controlled trial
PCCTU	The Oxford Primary Care and Vaccines Collaborative Clinical Trials Unit
PI	Principal Investigator
PP	Per-protocol analysis
PPI	Patient and Public Involvement
QOF	Quality and Outcomes Framework
QALY	Quality Adjusted Life Year
QoL	Quality of Life
R&D	NHS Trust R&D Department
REC	Research Ethics Committee
RGEA	Research Governance, Ethics & Assurance Team
SAE	Serious Adverse Event
SAR	Serious Adverse Reaction
SmPC	Summary of medicinal Product Characteristics
SOP	Standard Operating Procedure
SPCR	School for Primary Care Research
SPRINT	Systolic blood PRessure InterventioN Trial
SUSAR	Suspected Unexpected Serious Adverse Reactions
TMF	Trial Master File
TMG	Trial Management Group
TSC	Trial Steering Committee

4. BACKGROUND AND RATIONALE

The population is ageing [4] and, consequently, the number of people living with age-related chronic conditions is increasing.[5] Polypharmacy is common in older persons, with up to 20% of those aged >80 years prescribed ten or more medications.[6] Polypharmacy is associated with increased risk of adverse drug reactions and frequent inappropriate prescribing.[7,8] Indeed, as many as 29% of elderly people are thought to receive potentially inappropriate prescriptions in Primary Care.[9]

Hypertension is the number one co-morbid condition in older people with multiple chronic conditions [10] and 52% of those aged >80 years are prescribed two or more antihypertensive medications (equivalent to approximately 1.25 million people in the UK).[11] Blood pressure lowering has been shown to be effective at preventing stroke and cardiovascular disease in healthy individuals aged >80 years with stage 2 hypertension (systolic blood pressure of >160mmHg).[2] However, more recent evidence suggests that larger blood pressure reductions and multiple antihypertensive prescriptions may be harmful in older people.[12,13] A meta-analysis by Bejan-Angoulvant *et al.,* found that large reductions in systolic blood pressure and higher intensity treatment may be associated with increased risk of all-cause mortality.[12] Evidence from observational studies also suggests that higher intensity blood pressure treatment is associated with increased risk of falls in older people,[14] although this is also disputed.[2]

Some patients consider the increased risk of falls and other adverse events to be as important as the risk of MI or stroke, particularly those taking medications for primary prevention of cardiovascular disease.[15] Thus, decisions over blood pressure lowering in the elderly, particularly the frail elderly, require the weighing of harms and quality of life. Studies of patients' attitudes towards hypertension treatment suggest there is widespread dislike of treatment and its side effects, fear of the long-term impact of taking medication, and consequent intentional non-adherence to treatment.[16] However, clinicians can often struggle to stop prescribing medication due to a perceived lack of evidence, fear of the reaction of other prescribers, and concern that patients will feel their care is being cut.[17,18]

Proposed trial in the context of previous research

The recent SPRINT trial[1] showed that treatment to lower blood pressure targets (120mmHg systolic) is associated with reductions in cardiovascular morbidity and mortality. Observed reductions in total mortality were also greater in patients aged >75 years than in younger individuals. However, these reductions were accompanied by an increased risk of adverse events, including syncope and emergency department admission with injurious falls, although the overall rates were low. Patients enrolled in the SPRINT trial[1] were considered to be comparable to those enrolled into the HYVET study,[2,19] and therefore less frail than general populations from Europe and North America.[20,21] SPRINT excluded patients with diabetes, stroke, dementia and those residing in a nursing home, and thus, represent a subgroup of older individuals. Indeed, applying the SPRINT inclusion/exclusion criteria to a general population of individuals aged >80 years registered at general practices in the UK, reveals that one third would not have been eligible for the trial, and these individuals would have been prescribed significantly higher numbers of cardiovascular medications (increased polypharmacy) and have approximately twice the cardiovascular co-morbidity than eligible patients (table 1). The ACCORD[3] trial demonstrates that intensive blood pressure lowering may not be effective in patients with co-morbid diabetes and is associated with significant increases in adverse events in this population. Thus, the OPTiMISE trial will specifically target those individuals with greater polypharmacy and co-morbidity.

Table 1. Characteristics of the general population aged >80 years who would have been eligible/not eligible for the SPRINT trial,[1] registered at 19 general practices in the West Midlands[11]

Characteristics	Not eligible for SPRINT[1] (SD or %)	Eligible for SPRINT[1] (SD or %)	Comparison of groups†	Higher in the eligible or non-eligible group?
Total population	1,350	2,291		
Demographics/risk factors				
Age (years)	85.1±4.3	85.0±4.3	0.749	Same
Sex (% female)	853 (63%)	1,497 (65%)	0.174	Same
Smoking status (% current)	84 (6%)	139 (6%)	0.851	Same
Systolic blood pressure (mmHg)*	135.5±24.1	144.5±10.3	<0.001	Eligible
Diastolic blood pressure (mmHg)*	72.5±11.2	76±9.1	<0.001	Eligible
Total cholesterol (mmol/l)*	4.4±1.1	5.0±1.1	<0.001	Eligible
HDL cholesterol (mmol/l)*	1.5±0.3	1.6±0.4	<0.001	Eligible
Prescribed treatment				
Prescribed at least 1 statin	649 (48%)	531 (23%)	<0.001	Not eligible
Prescribed at least 1 antiplatelet	676 (50%)	720 (31%)	<0.001	Not eligible
Prescribed at least 1 antihypertensive	1,061 (79%)	1,397 (61%)	<0.001	Not eligible
Prescribed at least 2 antihypertensives	766 (57%)	838 (37%)	<0.001	Not eligible
Prescribed 3 or more antihypertensives	383 (28%)	299 (13%)	<0.001	Not eligible
Co-morbidities				
Diabetes	477 (35%)	0 (0%)	<0.001	Not eligible
Chronic kidney disease	544 (40%)	576 (25%)	<0.001	Not eligible
Myocardial Infarction	149 (11%)	145 (6%)	<0.001	Not eligible
Coronary heart disease	383 (28%)	358 (16%)	<0.001	Not eligible
Stroke	210 (16%)	0 (0%)	<0.001	Not eligible
Transient ischemic attack	108 (8%)	123 (5%)	0.002	Not eligible
Heart Failure	172 (13%)	128 (6%)	<0.001	Not eligible
Peripheral vascular disease	130 (10%)	140 (6%)	<0.001	Not eligible
Total cardiovascular disease	701 (52%)	595 (26%)	<0.001	Not eligible

**Most recently recorded †Comparisons of continuous variables with independent samples t-test, comparisons of binary variables using Pearson's chi squared test; SD=standard deviation; HDL=high-density lipoprotein; Cardiovascular disease defined as myocardial infarction, coronary heart disease, stroke, transient ischemic attack, heart failure or peripheral vascular disease.*

Whilst reducing the number of antihypertensive drugs prescribed to certain older patients may be beneficial, the lack of evidence to support such an approach limits the practice in routine clinical care. We have found limited evidence from randomised trials examining the safety of antihypertensive medication reduction or withdrawal. A systematic review of medication withdrawal studies was identified which included four small trials (with between 63 and 202 participants) examining diuretic withdrawal; this demonstrated withdrawal was maintained at follow-up in 51-81% of participants.[22] The recent DANTE study[23] examined the effect of complete antihypertensive medication discontinuation in 385 patients over the age of 75 years and with mild cognitive deficits. After 16 weeks of follow-up, they observed a 7/3mmHg increase in blood pressure but no difference in overall cognition compound score between groups (0.02 [−0.19 to 0.23]; P = 0.84) or quality of life (−0.09 [−0.34 to 0.16; P = 0.46]).

We identified one observational study,[24] which suggested that discontinuation of antihypertensive therapy may increase the risk of cardiovascular mortality in older people (>60 years), although this risk decreased overtime. The HYVET trial[2] did enrol some patients on antihypertensive treatment who were then randomised to placebo (effectively complete medication withdrawal), but there are no specific trials comparing a specified strategy of antihypertensive medication reduction with usual care in terms of effects on blood pressure control and quality of life. In addition, we have identified no previous economic modelling of a strategy of medication reduction in the elderly.

Importance of this research

The aim of this work will be to examine whether antihypertensive medication reduction in patients with controlled systolic hypertension (≤150mmHg) who are being prescribed two or more antihypertensives is possible without significant changes in blood pressure control at follow-up. This trial is needed because it is not clear what effect an intervention of medication reduction will have on blood pressure level at follow-up. Medication reduction might cause blood pressure to increase (removal of a treatment that is having a beneficial effect), which the SPRINT trial suggests may lead to adverse outcomes. In this instance, medication reduction would be deemed unsafe and treatment would be re-instated. However, the present trial will be recruiting patients who may have been taking medications for many years, potentially much longer than those enrolled into the SPRINT trial. Indeed, blood pressure may not increase with medication reduction, it might actually go down, since prescription of fewer antihypertensive therapies is associated with better adherence to medication[25] which could result in reduced blood pressure in the context of medication reduction. Alternatively, blood pressure level might not change at all, since patients may be non or partially adherent to prescribed therapy, and therefore removal of one medication may have little effect on overall blood pressure level. Indeed, just under half of individuals' prescribed antihypertensive therapy are thought to be non-adherent 12 months after the initial prescription.[26] It is these unknowns which require further investigation and provide the rationale for conducting this trial.

Older people are frequently excluded from trials [27] and our patient and public involvement suggests that some older individuals may be reluctant to participate in a clinical trial involving randomisation to new management strategies. However, previous Primary Care based studies suggest it is possible to recruit older participants to studies of cardiovascular disease prevention [2,28] and a recent survey suggested that older individuals are willing to participate in trials for reasons of curiosity, self-interest and altruism.[29] A recent review,[30] outlined how qualitative methods may assist in ensuring robust trial procedures and interventions, including overcoming barriers to effective recruitment. The OPTiMISE trial has several potential areas of sensitivity for both patients and professionals around de-prescribing medication, and

little research to date has explicitly focused on attitudes to reducing treatment in older people. Because of these areas of uncertainty, the study will have a staggered start, with two feasibility phases and concurrent qualitative work. These stages will allow aspects of trial feasibility such as recruitment to be assessed in a small sample, before recruitment to the main trial begins. Understanding the concerns of both patients and practitioners on these issues will be crucial to the development of the study approach and materials, and to high recruitment rates.

5. OBJECTIVES AND OUTCOME MEASURES

Objectives	Outcome Measures	Timepoint(s) of evaluation of this outcome measure (if applicable)
**Primary objective**<br>To determine if a reduction in medication can achieve a proportion of patients with clinically safe levels (defined as the proportion of patients with SBP <150mmHg) which is non-inferior (within 10%) to that achieved by the usual care group.	The proportion of patients with controlled blood pressure levels at 12 week follow-up.	Baseline and 12 week follow-up.
**Secondary objectives**<br>Determine the proportion of patients in intervention arm who maintain medication reduction through to follow-up (*i.e.* are *not* restarted on therapy due to unsafe increases in blood pressure)	Proportion of patients randomized to the intervention arm who maintain medication reduction throughout follow-up.	12 week follow-up.
Determine the difference in quality of life (according to EQ-5D-5L) between groups at follow-up.	EQ-5D-5L score at 12 week follow-up.	Baseline and 12 week follow-up.
Determine the difference in frailty (according to the FRAIL scale/frailty index) between the two groups at 12 week follow-up.	FRAIL scale score/frailty index at 12 week follow-up.	Baseline and 12 week follow-up.
Determine the mean difference in the change in mean clinic systolic blood pressure (from baseline) between the two groups at 12 week follow-up.	Change in mean clinic systolic blood pressure from baseline at 12 week follow-up.	Baseline and 12 week follow-up.

Determine the mean difference in the change in mean clinic diastolic blood pressure (from baseline) between the two groups at 12 week follow-up.	Change in mean clinic diastolic blood pressure from baseline at 12 week follow-up.	Baseline and 12 week follow-up.
Determine the difference in reported potential side effects to medication between the two groups at 12 week follow-up (e.g. coughs, dizziness, syncope, ankle swelling, etc.).	The proportion of patients reporting possible side effects to medication (e.g. coughs, dizziness, syncope, ankle swelling, etc.).	The number of possible side effects experienced by patients in each arm of the trial at 12 week follow-up.
Determine the difference in routinely reported adverse events between the two groups at 12 week follow-up (hospitalisation due to serious falls, myocardial infarction, stroke or all-cause mortality).	The proportion of patients reporting serious adverse events (hospitalisation due to serious falls, myocardial infarction, stroke or all-cause mortality).	The number of adverse events experienced by patients in each arm of the trial at 12 week follow-up.
Establish the characteristics of the baseline screening population, sample population and how these relate to individuals eligible/not eligible for the recent SPRINT trial.[1]	• Descriptive statistics of the screening and baseline population.<br>• Comparison of these characteristics with those eligible/not eligible for the SPRINT trial.	Baseline only.
**Exploratory analyses**<br>Subgroup analyses of blood pressure control, change in blood pressure and maintenance of medication reduction, by different levels of baseline frailty	The following outcomes, stratified by baseline frailty (frailty index score):<br>• The proportion of patients with controlled systolic blood pressure levels (systolic blood pressure <150mmHg) at 12 week follow-up.<br>• Change in mean clinic systolic blood pressure (from baseline) at 12 week follow-up.<br>• Proportion of patients who maintain medication reduction throughout follow-up.	Baseline and 12 week follow-up.
Subgroup analyses of blood pressure control, change in blood pressure and maintenance of medication reduction, by different levels of baseline functional independence	The following outcomes, stratified by baseline functional independence (modified Rankin Scale):<br>• The proportion of patients with controlled systolic blood pressure levels (systolic blood pressure <150mmHg) at 12 week follow-up.	Baseline and 12 week follow-up.

	• Change in mean clinic systolic blood pressure (from baseline) at 12 week follow-up.<br>• Proportion of patients who maintain medication reduction throughout follow-up.	
Subgroup analyses of blood pressure control, change in blood pressure and maintenance of medication reduction, by different levels of baseline cognitive function	The following outcomes, stratified by baseline cognitive function (MOCA score):<br>• The proportion of patients with controlled systolic blood pressure levels (systolic blood pressure <150mmHg) at 12 week follow-up.<br>• Change in mean clinic systolic blood pressure (from baseline) at 12 week follow-up.<br>• Proportion of patients who maintain medication reduction throughout follow-up.	Baseline and 12 week follow-up.
Subgroup analyses of blood pressure control, change in blood pressure and maintenance of medication reduction, by number of antihypertensive medications prescribed at baseline	The following outcomes, stratified by number of antihypertensive medications prescribed at baseline:<br>• The proportion of patients with controlled systolic blood pressure levels (systolic blood pressure <150mmHg) at 12 week follow-up.<br>• Change in mean clinic systolic blood pressure (from baseline) at 12 week follow-up.<br>• Proportion of patients who maintain medication reduction throughout follow-up.	Baseline and 12 week follow-up.
Subgroup analyses of blood pressure control, change in blood pressure and maintenance of medication reduction, by number of co-morbidities at baseline	The following outcomes, stratified by number of co-morbidities at baseline:<br>• The proportion of patients with controlled systolic blood pressure levels (systolic blood pressure <150mmHg) at 12 week follow-up.<br>• Change in mean clinic systolic blood pressure (from baseline) at 12 week follow-up.<br>• Proportion of patients who maintain medication reduction throughout follow-up.	Baseline and 12 week follow-up.

**Qualitative sub study 1 objective**<br>Determine the barriers and facilitators for patients and GPs to reducing antihypertensive medication to inform both the ongoing trial and potential future implementation.	• Thematic analysis of chart-stimulated interviews with GPs.<br>• Thematic analysis of 'Brown bag' medication review interviews with patients.	Interviews to be carried out throughout the trial.
**Qualitative sub-study 2 objective**<br>Determine how trial recruitment is discussed and understood by recruiters and patients.	Thematic analysis of audio-recorded recruitment appointments.	Interviews to be carried out throughout the trial.
**Economic sub study objective**<br>Determine the cost-effectiveness of the intervention in terms of cardiovascular, quality of life and cost outcomes	Cardiovascular disease risk, costs and quality-adjusted-life years.	Cost-effectiveness modelling carried after final follow-up in the analysis phase of the trial.

6. TRIAL DESIGN

This trial will use a Primary Care based, open label, randomised controlled trial design. Potential participants will be invited to attend a screening visit at their GP practice and those fulfilling the eligibility criteria and giving informed consent will undergo baseline measurements for the study. Extracted data will be entered directly into the study database using eCRFs. Following baseline measurements, individuals will be randomised to a strategy of medication reduction (intervention) or usual care (control) (see Appendix A for study flow diagram). Those in the intervention arm will be invited to self-monitor their blood pressure, reporting any consistently high readings to their GP/other appropriate, delegated healthcare professional (see specific self-monitoring guidance below). All individuals in the intervention arm of the trial will be asked to attend a routine safety follow-up visit with their GP/other appropriate, delegated healthcare professional, four weeks (±2 weeks) after randomisation. All patients will attend a 12 week (±2 weeks) follow-up with the trial facilitator, either at their GP practice or at their home; the trial facilitator will repeat all measurements taken at baseline. After 12 week follow-up there will be no further face-to-face visits, but passive long-term follow-up of mortality, hospital admissions and primary care data (see Appendix H) will be undertaken via NHS Digital's patient tracking service and medical notes review.

7. PARTICIPANT IDENTIFICATION

7.1. Trial Participants

Patients eligible for the trial will be aged ≥80 years, with controlled blood pressure (systolic blood pressure <150mmHg) receiving ≥2 antihypertensive medications with no compelling indication for medication continuation and whom the GP considers may benefit from medication reduction due to existing polypharmacy, co-morbidity and frailty. A broad inclusion criteria has been chosen to make the results of this study as generalisable as possible, an important priority for all Primary Care based trials. This includes enrolling patients on long term medication for secondary prevention of cardiovascular disease who, whilst at risk of further cardiovascular events, may also be more frail and at greater risk of falls and other adverse events, and thus benefit from medication reduction. Potentially eligible patients will be identified from electronic health records using a pre-defined search strategy which can be emailed to participating practices.

7.2. Inclusion Criteria

- Participant is willing and able to give informed consent for participation in the trial.
- Male or Female, aged 80 years or above.
- Clinic systolic blood pressure less than 150 mmHg (according to screening measurement at baseline – clinic blood pressure defined as the mean of the 2nd and 3rd readings taken at 1 minute intervals).
- Prescribed two or more antihypertensive medications to lower blood pressure for at least 12 months prior to trial entry. Antihypertensive medications defined as any ACE inhibitor, angiotensin II receptor blocker, calcium channel blocker, thiazide and thiazide-like diuretic, potassium-sparing diuretic, alpha-blocker,beta-blocker, vasodilator antihypertensives, centrally acting antihypertensives, direct renin inhibitors, adrenergic neurone blocking drugs or loop diuretics.
- Stable dose of antihypertensive medications for at least four weeks prior to trial entry.
- In the Investigator's opinion, could potentially benefit from medication reduction due to existing polypharmacy, co-morbidity, non-adherence or dislike of medicines and/or frailty (i.e. is different from those to which the results of the SPRINT trial are likely to apply)*
- In the Investigator's opinion, is able and willing to comply with all trial requirements.

*GPs will be given training from the research team during the site initiation visit on the findings of the SPRINT trial and other relevant trials and how these apply to patients in their practice.

7.3. Exclusion Criteria

The participant may not enter the trial if ANY of the following apply:

- A participant has heart failure due to left ventricular systolic dysfunction (LVSD) and is on only ACE inhibitors/ARBs and/or beta-blockers and/or spironolactone (removing any of which would be contraindicated).
- A participant has heart failure but has not had an echocardiogram since its onset (might have undiagnosed LVSD and a compelling need for ACEI/ARB and Betablockers).
- Investigator deems that there is a compelling indication for medication continuation.
- Suffered a myocardial infarction or stroke within the past 12 months.
- Blood pressure being managed outside of primary care.
- A participant with secondary hypertension.

- A participant with previous accelerated or malignant hypertension.
- Unable to provide consent due to incapacity.
- Any other significant disease or disorder which, in the opinion of the Investigator, may either put the participants at risk because of participation in the trial, or may influence the result of the trial, or the participant's ability to participate in the trial (e.g. terminal illness, house bound and unable to attend baseline and follow up clinics).
- Participants who have participated in another research trial involving antihypertensive medication in the past 4 weeks.

Please note, full details of inclusion and exclusion criteria for participants enrolled into the qualitative sub-studies are given in Section 10.

8. TRIAL PROCEDURES

A schedule of procedures can be found in Appendix A and B.

8.1. Recruitment

8.1.1. Practice and GP Recruitment

All practices within the study regions (defined according to proximity with research centres) will be approached by the study team and the NIHR Clinical Research Network (CRN) with a 1-2 page Research Information Sheet for Practices (RISP) detailing the study and the GP involvement required. Our PPI engagement suggests that older patients are much more open to the idea of medication reduction if it is suggested by their own trusted GP and so full engagement from GPs will be critical to ensuring the trials' success. GPs are busy and often have little time to read through extensive study literature when considering participation in a new trial. A two minute video infographic (explaining the study rationale, which patients will be eligible and what it will involve) will also be emailed to all GPs.

8.1.2. Practice database searches

Prior to patient invitation, data will be extracted from all participating practice computer systems related to the demographics of the practice population, cardiovascular disease history, the presence of other co-morbidities, medication prescribed and overall frailty examined using the electronic frailty index (eFI).[31] Searches will be designed and conducted using the MIQUEST query tool for use in Vision practices and adapted for other practice database systems (e.g. EMISWeb) where appropriate. These data will be used to describe the general practice population, and identify who is eligible for invitation to the trial. GPs will also use these data to assess the patient's suitability to participate, including whether the patient's level of polypharmacy, co-morbidity and/or frailty means that they could potentially benefit from medication reduction. GPs will be given training by the research team at the site initiation visit regarding how to distinguish these patients from those in which recently published trials (i.e. SPRINT)[1] suggest may benefit more from medication continuation. These data will also enable the research team to examine the proportion and characteristics of individuals who would have been eligible for previous blood pressure lowering trials conducted in the elderly[1-3] and compare these to the population invited and recruited to OPTiMISE.

8.1.3. Patient Recruitment

Participants will be selected from practices across the UK. Potentially eligible patients will be identified by trained practice staff searching practice-based registers for people on two or more antihypertensive medications whose last systolic blood pressure was recorded to be <150 mmHg. Those deemed eligible will be sent letters of invitation from their GP. Patients interested in participating will be asked to return an expression of interest slip by post, email or call the study team directly using the study telephone number. Patients contacting the study team at a trial recruiting centre will be invited to attend an initial screening, recruitment and baseline clinic at their general practice (see flow chart in Appendix A). They will also be asked if they would like to receive the study video infographic via email (all potential participants will view the video infographic at the consent visit so access to email will not affect access to information about the study). Patients not responding to the first invitation will receive one reminder letter (up to four weeks after the first letter) or if possible, a direct telephone call inviting them to participate. All follow-up telephone calls will be made by practice staff and potential participants will not be contacted directly by research staff until they have expressed an interest in participating in the study.

Potentially eligible patients may also be approached opportunistically by a member of the clinical care team at a routine clinical follow-up appointment, or during a [nursing] home visit. Those who do not wish to take part may be asked to fill in a short questionnaire detailing their reasons.

Given the age and potential lack of independence of the study population, simple, clear provision of information is likely to be important, as is engagement of carers. Indeed, evidence suggests that most patients base their informed decision on whether or not to participate in a research study on limited information.[32] Therefore, in addition to the usual patient information sheet (PIS), a simplified 2-page patient information summary sheet will be prepared summarising what will be required from participants enrolled into the study. This cover sheet will link to each section of the PIS which will provide more detail for each area. A separate, simplified information sheet for carers will also be prepared detailing the support that will be required from carers for patients choosing to participate in the study. All individuals attending a screening visit will be sent a copy of the study patient information sheet (PIS), the cover sheet, the carers information sheet and consent form so that they have chance to look at it prior to attending the clinic.

Full details of practice, GP and participant recruitment for the qualitative sub-studies are given in Section 10.

8.2. Informed Consent

Informed consent will be taken by the GP, after which the participant will move to another room for baseline screening measurements and data collection. In the invitation letter, patients will be asked if they are happy for initial study visits to be audio-recorded for qualitative analysis of recruitment appointments and data collection procedures (see section 10.2 for details). Potential participants who are happy for audio-recording of appointments will be asked to hand a signed response slip (included in the invitation letter) to the practice receptionist upon arrival for their first study visit. Consent to audio recordings will not have a bearing on an individual's care or eligibility for the main trial.

Prior the patient's appointment, participating GPs will review the patient's current antihypertensive medication regime and decide which medication should be removed if the participant is randomised to the intervention arm of the trial (see details of the intervention below). The choice of medication to be reduced, and reasons why, will be documented and pass on to the trial facilitator. The patient will not be informed of the choice of medication. During the patient appointment, the GP will show the study video

infographic and go through the full PIS explaining the exact nature of the trial; what it will involve for the participant; the implications and constraints of the protocol and any risks involved in taking part.

Having discussed the study with the GP, and having had a chance to ask questions, those individuals willing to participate will be asked by the GP to give informed consent adhering to the relevant PC CTU Standard Operating Procedure (SOP). The patient will have read the PIS which details the study, what is required of patients, discusses potential risks and benefits and provides contact details of the research team. It will be clearly stated that the participant is free to withdraw from the trial at any time for any reason without prejudice to future care, without affecting their legal rights and with no obligation to give the reason for withdrawal.

Given the older age of the population being studied, GPs will be allocated up to 20 minutes to explain the trial to potential participants (standard trials would usually allocate 10 minutes), plus an additional 10 mins prior to meeting with the patient, to assess suitability and decide on the appropriate medication for withdrawal (30 mins per patient in total). The participant will be allowed as much time as wished to consider the information, and the opportunity to question the Investigator, their GP or other independent parties to decide whether they will participate in the trial. Due to the CTIMP status of this trial, individuals lacking capacity to give informed consent will excluded. The number of patients excluded for this will be monitored during the feasibility study and if it is deemed prohibitive to recruitment rates, alternative strategies will be explored with the relevant approvals for these sought via submission of a protocol amendment.

Written Informed Consent will be obtained by means of participant dated signature and dated signature of the person who presented and obtained the Informed Consent. The GP who obtained the consent must be suitably qualified (*i.e.* have received training in GCP) and experienced, and have been authorised to do so by the Principal Investigator. The participant or legally authorised representative must personally sign and date the latest approved version of the Informed Consent form before any trial specific procedures are performed. A copy of the signed Informed Consent will be given to the participant. The original signed form will be sent to the PC CTU, one copy retained at site and one with the participant.

8.3. Screening and Eligibility Assessment

Those giving informed consent will then move to another room in the practice where a trained member of the research team (PCRN/research/practice staff) will complete the screening procedures which include confirmation of the patient's age, past medical history (e.g. history of stroke or heart attack in the past 12 months), current cardiovascular medication, and measurement of blood pressure.

8.4. Baseline Assessments

Remaining baseline data will be collected following confirmation of eligibility via patient questionnaires and a detailed notes review conducted by the research assistants. Variables to be collected are listed below in Appendix B. Blood pressure will be measured using the clinically validated[33] BpTRU blood pressure monitor which automatically records six blood pressure measurements at one minute intervals. Readings will be taken after participants have been seated for five minutes of rest and the mean of the 2nd and 3rd readings will be used the define the primary outcome. To test for orthostatic hypotension, two further readings will be taken in the standing position after one and three minutes.[34] Orthostatic hypotension will be defined as a $\geq$20mmHg drop in systolic blood pressure within three minutes of standing.

Patient characteristics and information about their medical history will be extracted from the practice records by the research assistant and entered directly into the study database. Patients will be asked to complete the following quality of life and frailty questionnaires[35-37] during their baseline and/or follow-up clinics:

- the EQ-5D 5L (Quality of life)[35]
- the self-report modified Rankin Scale (functional independence)[37]
- the FRAIL Scale[36]
- Self-report domains of the Frailty index[31,38,39] (see below)
- the Montreal Cognitive Assessment [MoCA])[40]
- the Medication Adherence Rating Scale (MARS) Questionnaire[41]

The frailty index is considered the most comprehensive frailty assessment[42] and can be estimated in part from a participant's medical records (in the present study it will be integrated into the electronic CRF so that certain items are not collected twice).[31] It should contain between 30-40 items of frailty (to which the answer is yes or no), but the specific number and type to include is flexible and can be adapted to a specific population or study type provided each item satisfies five simple criteria.[38,43] The index is derived by dividing the number of frailty criteria present by the number of items assessed. The Frailty index to be used in the present study is given in Appendix C.

The 5-item FRAIL scale can be completed by the patient themselves and covers components of fatigue, resistance, ambulation, illness and weight loss. A score of 1 is attributed to each component and patients with a total score of 3-5 are classed as frail. Those with a score of 0 are considered healthy.

All questionnaire data, where possible, will be collected on a tablet computer linked to the study database. Participants will be given the option to enter responses themselves or with assistance from the research assistant. Where questionnaires are not validated for use on a tablet computer,[35] or where individuals are not comfortable using one, paper copies will be made available for completion.

8.5. Randomisation, blinding and code-breaking

Consenting patients who have completed baseline assessment will be individually randomised to one of two study arms using a web based system (Sortition®) with manual Primary Care Clinical Trials Unit (PC-CTU) back up. Participants will not be randomised until after consent has been taken and baseline assessments have been completed. Randomisation will use minimisation on practice and baseline systolic blood pressure to ensure each arm is balanced and 1:1 allocation is achieved once all participants have been recruited. The CTU programmer will test and validate the minimisation schedule to ensure the process is reproducible.

Patients randomised to the intervention will be invited to self-monitor (or have a carer monitor) their blood pressure every day for the last week of every month during the follow-up period (weeks 4, 8 and 12). Those willing to do so, will be loaned a validated blood pressure monitor for the duration of the study. We have experience of getting patients to self-monitor their blood pressure from the TASMINH-SR trial[44] and will provide the same 'traffic light system' used in that trial to identify consistently high readings requiring action by the patient (Appendix F). This action will be to schedule an appointment with their GP/other appropriate, delegated healthcare professional for further assessment of blood pressure and potential re-introduction of therapy.

The study will use an open label design, so patients and practitioners will not be blinded to the intervention or study endpoints but assessment of outcomes will be blinded to the intervention allocation. Thus, codebreaking will not be necessary.

8.6. Subsequent visits

Participants will attend one research follow-up clinic 12 weeks (±2 weeks) after baseline and those in the intervention will attend one additional safety visit at four weeks (±2 weeks). This period is expected to be sufficiently long enough to assess the impact of antihypertensive medication reduction, since these drugs usually take approximately four weeks to 'wash out' of a patient's system. Earlier safety visits are not recommended since they could provide false reassurance that blood pressure is within safe limits if the withdrawn drug has not washed out of the participant's system.

Follow-up assessments to be conducted at each clinic are detailed in Appendix B and will include standardised blood pressure measurement (for assessment of the primary outcome), patient lifestyle characteristics, and prescribed medication. All patients attending follow-up will be asked to repeat the questionnaire assessments conducted at baseline. They will also be expected to report on their adherence to the trial medication regime and any side effect and adverse events suffered (not already documented). Follow-up appointments may be recorded (with patient consent) to permit qualitative assessment of patient experiences during the trial.

Regardless of whether individuals in the intervention arm agree to self-monitor, all those undergoing medication reduction will be asked to return to their GP/other appropriate, delegated healthcare professional for a routine safety follow-up visit approximately four weeks after randomisation. During this safety follow-up, the GP/nurse/other appropriate, delegated healthcare professional/healthcare assistant will examine the patient's blood pressure and GP/other appropriate, delegated healthcare professional may invite the patient for a further follow-up visit to recheck and adjust medication (dose or type) if adverse events occur or if blood pressure is sustained above 150 mmHg (Appendix E provides flowchart that GPs/other appropriate, delegated healthcare professionals are asked to follow).

All patients will be flagged for mortality, hospital admissions and primary care data using NHS Digital's patient tracking service, and via medical notes review, permitting long term follow-up for up to 5 years. Participants will be provided with detailed information on the enhanced long term follow-up and reminded of the option to opt out.

8.7. Internal feasibility study

A trial of this type presents a number of challenges, particularly related to the recruitment of older individuals and the sensitive nature of the intervention under examination. A two stage internal feasibility study will be conducted to examine methods of patient invitation and rates of recruitment carefully, before proceeding with the main trial.

8.7.1. Feasibility phase 1

The first feasibility phase will last for a minimum of 3 months and aim to recruit approximately 25 patients from a minimum of 3-5 practices to establish whether or not anyone will be willing to participate in the study. Practices and patients will be approached for potential participation as outlined above.

8.7.2. Feasibility phase 2

The second feasibility phase of the trial will focus on recruitment rates for the main trial and whether the intended sample size is likely to be met during the recruitment period. A recruitment rate of approximately 15% of those invited is anticipated. The recruitment rate will be estimated from the those enrolled during the first feasibility phase and a further 75 patients from approximately ten practices recruited during a second phase of at least 6 months, giving an anticipated sample of 100 participants. The following actions will be considered to address varying rates of recruitment in both feasibility phases:

- If ≥100 patients are recruited – trial will proceed as planned
- If 75-99 patients are recruited – recruitment materials/method will be re-examined with discussions with stakeholders and patient and public involvement representatives.
- If 50-74 patients are recruited – the allocation of resources and recruitment criteria will be re-examined using information gathered from concurrent qualitative work.
- If <50 patients are recruited – the Trial Steering Committee (TSC) will decide, in discussion with the Data Monitoring and Ethics Committee (DMEC) and the funders, whether the trial should be stopped due to futility.

8.8. Discontinuation/Withdrawal of Participants from Trial Treatment

Each participant has the right to withdraw from the trial at any time. In addition, the Investigator may discontinue a participant from the trial at any time if they consider it necessary for any reason including:

- Ineligibility (either arising during the trial or retrospectively having been overlooked at screening)
- Significant protocol deviation
- An adverse event which results in inability to continue to comply with trial procedures
- Withdrawal of Consent
- Loss to follow up

An intention-to-treat (ITT) approach will be taken so that even if medication is re-introduced to patients in the intervention group, or a patient in the control group has medication withdrawn, we will ask all participants to attend all follow-up visits as far as is practicable. The proportion of patients who successfully maintain medication reduction is a secondary outcome of this trial and thus capturing this accurately at follow-up is important. Unless a participant withdraws consent, vital status will be assessed even where an individual has been lost to follow-up (for instance moved away).

The reason for withdrawal will be recorded in the CRF. If the participant is withdrawn due to an adverse event, the Investigator will arrange for follow-up visits or telephone calls until the adverse event has resolved or stabilised.

8.9. Definition of End of Trial

The formal end of trial is the date of the last data capture following the last visit of the last participant.

9. INVESTIGATIONAL MEDICINAL PRODUCT (IMP)

9.1. Intervention group (IMP Description)

This study will use an open label design, so no blinding of the treatment allocation, or encapsulation of trial medications will be used, although treatment allocation will be concealed prior to consent and baseline assessment. Patients allocated to the intervention group of the trial will have one antihypertensive medication of the treating GP's choice stopped, in line with existing guidelines, where appropriate. Specifically, participating GPs will be encouraged to identify previously unrecognised contraindications to medication, defined by the STOPP criteria[45] (see below), and withdraw this medication:

- Thiazide diuretic with a history of gout (may exacerbate gout).
- Beta-blocker in combination with verapamil (risk of symptomatic heart block).
- Non-cardioselective beta-blocker with chronic obstructive pulmonary disease (risk of bronchospasm).
- Calcium channel blockers with chronic constipation (may exacerbate constipation).
- Use of diltiazem or verapamil with NYHA Class III or IV heart failure (may worsen heart failure).

In the absence of any obvious contraindications, or a strong clinical reason for continuing despite a STOPP criteria being met, GPs will be asked to reduce antihypertensive medications in reverse of the NICE C+A+D algorithm for older patients,[46] removing the most recently prescribed therapy beginning with thiazide (or thiazide-like) diuretics, ACE inhibitors or angiotensin II receptor blockers and then calcium channel blockers (see Appendix D). The decision to reduce antihypertensive medication will require medical input based on indications, co-morbidities and blood pressure and whilst the study team will provide the aforementioned withdrawal algorithm, the final decision will be left to the consulting GP. All patients in the trial will remain on at least one antihypertensive (the aim of the trial to assess the safety of removing one antihypertensive, not examine the optimal number/schedule of medications to reduce).

Once a medication has been removed, GPs/other appropriate, delegated healthcare professionals will be expected to closely monitor the participant's response to medication reduction carefully. GPs/other appropriate, delegated healthcare professionals will be given advice about what and when to monitor (Appendix E) but this will be left flexible to allow the GP/other appropriate, delegated healthcare professional to manage the patient in the way they see best. Broadly speaking, patients will be expected to return to their GP for at least one routine safety follow-up visit around 4 weeks after randomisation (±2 weeks). If systolic blood pressure increases beyond what is considered clinically safe (≥150mmHg, current target recommended by NICE)[46] during this visit, the patient will be asked to return for further safety follow-ups and if the raised blood pressure persists, or adverse events occur, GPs/other appropriate, delegated healthcare professionals will be expected to re-adjust medication (dose or type) in line with Appendix E, rendering the likelihood of a serious adverse event occurring very low.

All participants randomised to the medication reduction arm of the trial will be offered a blood pressure monitor for self-monitoring of blood pressure. They will be trained using protocols developed in the previous TASMIN trials[44,47] and will be given simple and clear instructions to contact their GP/other appropriate, delegated healthcare professional if blood pressure rises above what is considered clinically safe (i.e. home systolic blood pressure >145mmHg on all readings) (see Appendix F). Patients will be asked to self-monitor (or have a carer monitor) at least 4 times per week in the last week of each month of follow-up (weeks 4, 8 and 12), although they can monitor more frequently if they wish. Differential use of self-monitoring in the intervention group, or indeed in the control group (many patients now self-monitor routinely) is not expected to impact on the study results, since there is good evidence that self-

monitoring only affects blood pressure levels if used in combination with a co-intervention.[48] All other clinical care will continue as usual.

In the event that participating in this study affects a practice's ability to meet QOF targets (*i.e.* those which recommend treatment to targets in specific patient subgroups which may not be met if antihypertensive medication is reduced), it will be recommended that relevant patients are exception reported as "not suitable" in all related QOF submissions.

9.2. Control group

Those allocated to the control arm of the study will continue usual clinical care (i.e. they will continue to take antihypertensive medications as prescribed and will not self-monitor unless already doing so). No other medication changes will be mandated and participating GPs will be asked to manage all other care according usual clinical practice. Individuals in the control group will not be given the option to self-monitor, although those who already self-monitor routinely (prior to the trial), or choose to begin during the trial will not be excluded.

9.3. Compliance with Trial Treatment

Since this is a trial of medication reduction, compliance with the trial treatment will involve not taking the medication, which has been de-prescribed. Because individuals in the intervention arm will not be given a prescription for the de-prescribed medication, it will be hard for them not to comply (and take therapy they should not be taking, unless they have a supply of tablets from prior to the de-prescribing of treatment). There are no validated instruments for measuring compliance with medication reduction. Nonetheless, participants will all be asked to recall if they have taken any de-prescribed medications during the follow-up period, at the 12 week visit, and their response will be documented on the CRF. Adherence to control treatments and remaining therapies (which have not been de-prescribed) will be examined at follow-up by giving each patient the Medication Adherence Rating Scale (MARS) Questionnaire.[41] GP prescribing data will be collected from practice computer systems by the research assistant as a measure of GP compliance with the study protocol.

9.4. Concomitant Medication

All other (non-blood pressure lowering) medication taken by participants will be at the discretion of participating practices. No other medication changes will be mandated and participating GPs will be asked to manage all other care according usual clinical practice. Prescribed and relevant over the counter medications taken will be recorded at baseline and follow-up.

9.5. Post-trial Treatment

Continuation of medication reduction after the trial is complete will be at the discretion of the consulting GP/other appropriate, delegated healthcare professional. The patient remains the responsibility of their GP during and after the trial, and therefore under will continue under normal care. The study team will not provide further guidance on medication reduction, or provide blood pressure monitors for self-monitoring of blood pressure outside the trial period.

10. QUALITATIVE SUB STUDIES

Embedded within the trial will be two qualitative studies: scoping work to understand the perspectives of patients and GPs and to inform recruitment approaches, followed by an iterative examination of recruitment within the trial. This work will be led and coordinated from Cambridge.

10.1. Qualitative study 1: interviews with doctors and patient

To generate understanding about the barriers and facilitators to reducing antihypertensive medications, and inform development of trial recruitment procedures and materials, we will conduct face-to-face interviews with GPs and patients. These will take place prior to the main trial.

10.1.1. Participant identification and recruitment

Both GPs and patients will be recruited to participate in the first interview study from practices within the Cambridgeshire study region. The study team will, in discussion with the NIHR Clinical Research Network (CRN), approach potential GP participants with an information sheet outlining what participation would involve. All interested GPs will be followed up by a member of the study team to discuss the interview and the requirements of the chart-stimulated recall approach (see below for details of this). In line with qualitative sampling approaches, we will seek a broad range of opinion by endeavouring to approach GPs working in varying practice settings, including larger and smaller practise sizes and both rural and urban locations. We anticipate interviewing around 15 GPs in total: analysis will commence alongside subsequent interviews to enable the study team to monitor the depth and range of data being collected.

GPs agreeing to participate in an interview will be asked, in collaboration with practice staff, to identify potential patients to additionally approach for interview. We will apply the same inclusion criteria as in the trial, seeking to interview patients aged ≥80 years, with controlled blood pressure (systolic blood pressure <150mmHg) receiving ≥2 antihypertensive medications with no compelling indication for medication continuation and whom the GP considers may benefit from medication reduction due to existing polypharmacy, co-morbidity and frailty. However, in contrast with the trial, the only exclusion criteria at interview will be capacity to consent to and participate in an interview, as determined by the GP. Those deemed eligible will be sent letters of invitation from their GP, including a participant information sheet and consent form. Patients will also be approached opportunistically, via a telephone call from their GP, or, in those participants enrolled into the main trial who agree, via a telephone call from the research team. Those expressing an interest in the study over the phone will be sent a participant information sheet and consent form.

Patients interested in participating will be asked to return an expression of interest slip by post, email or call the study team directly using the study telephone number: a researcher will then arrange a convenient time for interview. Patients not responding to the first invitation will receive one reminder letter (up to four weeks after the first letter) or if possible, a direct telephone call inviting them to participate. All follow-up telephone calls will be made by practice staff and potential participants will not be contacted directly by research staff until they have expressed an interest in participating in the study.

Interviews will take place at a convenient location for the patient, such as in their own home or, if they prefer, at their GP practice. As with GPs, we anticipate conducting around 15 interviews with patients to generate sufficient data for the purposes of our analyses.

10.1.2. Informed consent

For both GPs and patients, written informed consent will be taken by the researcher prior to the commencement of each interview. If participants have previously sent a consent form to the study team prior to the date on which the interview takes place, this will be reviewed and verbally re-confirmed. Consent forms will include permission to audio-record the interview and for anonymised quotes to be used in research reports and publications.

10.1.3. Interview approach

Interviews with GPs will use a chart-stimulated recall approach to explore the factors, which influence their treatment choices in older hypertensive patients. We will draw on anonymised records from patients eligible for the main trial, using these to focus discussions about how GPs would feel about reducing antihypertensive medications in these patients. To achieve this, participating GPs will be asked, prior to the interview, to identify two patients whose clinical cases they would like to reflect on. Patient anonymity will be protected at all times: GPs will be asked not to divulge patient-identifiable information during interviews, such as names or residential locations. During the interview, discussions will include how a medication reduction decision might vary between patients, and include open-ended questions focusing on the doctor's approach to the management of hypertension and how this has changed over time.

Interviews with patients will use 'brown bag' medication review techniques[49] to work together during the interview to create a complete record of medication held, with a commentary on usage from the participants' perspective. Following this logging exercise, we will use diagrammatic elicitation techniques in which interviewees are supported to complete a relational map outlining their conditions and medications and their perceived inter-relationships and meaning. These sketches will be used as the basis for a discussion on the implications of withdrawing antihypertensive medications, and what this "gap" might mean for the patient. Open-ended questions will focus on perceptions of their need for and role of antihypertensives, experiences of being on antihypertensives, and perceived needs after cessation of treatment.

10.1.4. Data analysis

All interviews that are transcribed will be transcribed verbatim. Visual data will be digitally scanned. All data will be stored and organised in NVivo. Interview and visual data from GP and patient interviews will be subjected to thematic analysis, with a particular orientation to exploring clinical and patient perspectives on the barriers and facilitators to reducing anti-hypertensives. Analyses will be used both inform the development of materials and approaches to be used in the trial and to understand GPs' and patients' attitudes to and concerns regarding medication burden and optimisation.

10.2. Qualitative study 2: assessment of trial recruitment and data collection procedures

The aim of this second qualitative study will be to inform understanding of the presentation of information within recruitment appointments, and how this might impact on consent to participate, with a view to ensuring robust procedures in an iterative process. We will draw on methods previously used in the ProtecT trial,[50] and further developed by the QuinteT (Qualitative Research Integrated in Trials)[51] team, aiming to facilitate the ability of patients to make an informed decision about their participation in the trial. To achieve this, we will audio record consultations between GPs/research assistants and eligible patients, to observe the nature of discussions about the OPTiMISE trial. This qualitative study is fully embedded within the conduct of the feasibility trial: full consent procedures are outlined in section 8.2. We will aim to record about 15 consultations at each of five practices in the internal feasibility study, giving us a pool of 75 consultations for analysis. Assuming recruitment rates of around 15% are achieved,

approximately 10 -12 observed consultations would include a patient who consents to participate. We will also record a subset of follow-up appointments to examine patient's experiences of participating in the trial.

Thematic analysis will be undertaken on a sample of around 15-20 consultations comprising patients who did/did not consent to participate, to consider (a) terminology used, (b) presentation of the deprescribing approach and (c) presentation of randomisation. This will inform on-going trial procedures and future implementation should the results suggest that medication reduction is an appropriate strategy in older individuals.

10.3. Integration of qualitative sub-studies with trial procedures

To ensure swift implementation of procedural changes as a result of themes identified through concurrent data analysis in the qualitative studies, we will hold two dissemination 'away days' with the study team. These days will be designed specifically to debate observations and analytical ideas identified through the qualitative interviews alongside the latest recruitment rates from the feasibility study, and to subsequently plan strategies to deal with any arising issues. They will offer a longer, more focused time to develop strategies which will maximise the success of the trial, compared to traditional trial steering committees. Monthly meetings across centres, and bi-annual steering committee meetings will also be held to ensure appropriate flow of information between all members of the multi-centre project team.

11. ECONOMIC SUB STUDY

We have previously developed Markov cost-effectiveness models to estimate the long-term costs and benefits from blood pressure lowering in younger populations.[52] These models do not include harms of treatment, which are assumed similar in both arms, an assumption which may not hold in an older population. We will adapt this model to include harms of treatment with adjustment of the effects of blood pressure lowering on cardiovascular disease risk, costs and quality-adjusted-life years (QALYs) to match the older population involved in this work. Particular attention will be given to how small changes in blood pressure level impact on patient outcomes, regardless of whether or not the trial demonstrates medication reduction to be non-inferior to usual care. Costs of the therapies prescribed, side effects and acute and long term costs of cardiovascular events will be obtained within the trial and from the literature. Quality of life on each treatment strategy will be obtained from the trial data on EQ-5D 5L, and previous studies will inform utility values for cardiovascular disease health states impact of side effects. The model will determine the cost per additional QALY gained of the medication reduction intervention versus usual care and analysis will be from a health and social services perspective. The model will be run over patient lifetime, with costs and benefits discounted at a rate of 3.5%. Extensive sensitivity analyses, including probabilistic sensitivity analysis, will evaluate parameter uncertainty and a value of information exercise will assess whether a further trial would be appropriate and which parameters would be most sensitive to change and should therefore be chosen as outcomes for such a trial. This work will be led by S Jowett (Honorary Senior Lecturer at Keele University).

12. SAFETY REPORTING

12.1. Definitions

Adverse Event (AE)	Any untoward medical occurrence in a participant to whom a medicinal product has been administered (or taken away), including occurrences which are not necessarily caused by or related to that product.
Adverse Reaction (AR)	An untoward and unintended response in a participant to an investigational medicinal product which is related to any dose which is/or is not administered to that participant.<br>The phrase "response to an investigational medicinal product" means that a causal relationship between a trial medication (or lack of) and an AE is at least a reasonable possibility, i.e. the relationship cannot be ruled out.<br>All cases judged by either the reporting medically qualified professional or the Sponsor as having a reasonable suspected causal relationship to the trial medication qualify as adverse reactions.
Serious Adverse Event (SAE)	A serious adverse event is any untoward medical occurrence that:<br>• results in death<br>• is life-threatening<br>• requires inpatient hospitalisation or prolongation of existing hospitalisation<br>• results in persistent or significant disability/incapacity<br>• consist of a congenital anomaly or birth defect<br>Other 'important medical events' may also be considered serious if they jeopardise the participant or require an intervention to prevent one of the above consequences.<br>NOTE: The term "life-threatening" in the definition of "serious" refers to an event in which the participant was at risk of death at the time of the event; it does not refer to an event, which hypothetically might have caused death if it were more severe.
Serious Adverse Reaction (SAR)	An adverse event that is both serious and, in the opinion of the reporting Investigator, believed with reasonable probability to be due to one (or lack of) of the trial treatments, based on the information provided.
Suspected Unexpected Serious Adverse Reaction (SUSAR)	A serious adverse reaction deemed by the investigator to be either related to the medication withdrawal (the study IMP) or the nature and severity of which is not consistent with the information about the medicinal product in question set out in the summary of product characteristics (SmPC) for that product.

NB: to avoid confusion or misunderstanding of the difference between the terms "serious" and "severe", the following note of clarification is provided: "Severe" is often used to describe intensity of a specific event, which may be of relatively minor medical significance. "Seriousness" is the regulatory definition supplied above.

12.2. Causality

The relationship of each adverse event to the trial medication must be determined by a medically qualified individual according to the following definitions:

- **Unrelated** – where an event is not considered to be related to the IMP
- **Possibly** – although a relationship to the IMP cannot be completely ruled out, the nature of the event, the underlying disease, concomitant medication or temporal relationship make other explanations possible
- **Probably** – the temporal relationship and absence of a more likely explanation suggest the event could be related to the IMP.
- **Definitely** – the known effects of the IMP, its therapeutics class or based on challenge testing suggest that the IMP is the most likely cause.

All AEs (SAEs) labelled possibly, probably or definitely will be considered as related to the IMP.

12.3. Trial specific issues around patient safety

This trial has important safety issues which are described and addressed below.

12.3.1. Risks of treatment/medication reduction

In this elderly, potentially frail population, the major risks of treatment are the potential for falls due to lower blood pressure which can lead to subsequent complications and sometimes death. Medication reduction could be associated with an increased risk of major cardiovascular events or cardiac failure. All patients enrolled into the trial will be informed of the risks of medication continuation and/or reduction in patient information sheets prior to consent and will be followed up carefully throughout the trial.

12.3.2. Trial follow-up

Potential 'side effects' to medication reduction will be monitored with self-monitoring of blood pressure and by the consulting GP/nurse/Healthcare Assistant/other appropriate, delegated healthcare professional at the scheduled 4 week follow-up. This period of follow-up was chosen because it will ensure complete drug washout (most treatment trials wait at least month between instructing patients to stop taking medication and measuring blood pressure in the trial run-in phase) and is in keeping with standard procedures when adding/removing drugs in routine practice. The trial is sufficiently short that if any serious adverse events were to occur in one of the trial arms (e.g. MI or stroke), the trial could be stopped before significant numbers of individuals came to harm.

12.3.3. Measures to minimise the risks associated with medication reduction

To ensure the risks to patients enrolled in the intervention arm of the trial are not unacceptably high, consulting GPs/other appropriate, delegated healthcare professionals will be asked to follow the flowchart in Appendix E. Consulting GPs/other appropriate, delegated healthcare professional's application of these criteria throughout the trial will be monitored by the data monitoring committee. Specifically, GPs/other appropriate, delegated healthcare professionals will be expected to re-introduce therapy if the patient presents with one of the following:

a) The patient has clinic systolic blood pressure reading >180 mmHg or clinic diastolic blood pressure reading > 110 mmHg (defined as the mean of 2nd and 3rd readings taken within the same visit).

b) The patient has a clinic systolic blood pressure reading ≥150 mmHg or clinic diastolic blood pressure reading ≥90 mmHg (defined as the mean of 2nd and 3rd readings taken within the same visit) at repeated safety follow-up visits.
c) The GP/other appropriate, delegated healthcare professional feels there is a clinical need for re-introduction of treatment

12.4. Recording Procedures for Adverse Events

All site staff will be appropriately trained in the procedures to follow and the forms to use by the PC-CTU prior to study initiation. Regular central monitoring for all studies and site monitoring, as determined by the trial specific risk assessment, will be used to ensure that all adverse events are identified and acted on appropriately.

Adverse events that are observed by the Investigator or reported by the participant may be reported at any time but will be specifically asked about and recorded on the CRF at 12 week follow-up, whether or not attributed to trial intervention.

The following information will be recorded: description, date of onset and end date, severity, assessment of relatedness to trial medication, other suspect drug or device and action taken. Follow-up information should be provided.

The severity of events will be assessed on the following scale: 1 = mild, 2 = moderate, 3 = severe. The severity of events, and the relationship of AEs to the study medication, will be assessed by the local medically qualified investigator or a medically qualified member of the research team. AEs considered related to the withdrawal of medication (the intervention), will be followed until resolution or the event is considered stable, clinically insignificant or asymptomatic. All related AEs that result in a participant's withdrawal from the study or are present at the end of the study, should be followed up until a satisfactory resolution occurs.

It will be left to the recruiting physician's clinical judgment whether or not an AE is of sufficient severity to require re-introduction of the participant's withdrawn treatment and the reason will be recorded. A participant may also voluntarily have treatment re-introduced due to what he or she perceives as an intolerable AE. If either of these occurs, the participant must be given appropriate care under medical supervision until symptoms cease or the condition becomes stable.

12.5. Reporting Procedures for Serious Adverse Events

All SAEs occurring during the study (from randomisation to the end of the individual's 12 week follow-up appointment), either observed by the recruiting physician or reported by the participant, whether or not attributed to study intervention, will be recorded and forwarded by the site to PC-CTU, using the "PC-CTU SAE Report Form" following assessment for seriousness and relatedness by the site clinician. This form will be completed and faxed and/or sent using secure email, to the PC-CTU using the number/email quoted on the report form. As a minimum, the following information will be recorded:

- Description
- Date of onset
- End date
- Severity
- Assessment of relatedness to study medication

- Other suspect drug or device
- Action taken

Follow-up information should be provided as necessary.

SAEs must be reported to the PC-CTU within 24 hours of discovery or notification of the event. The PC-CTU will acknowledge receipt of the SAE Report Form using the PC-CTU 'SAE Form Receipt' document. This receipt will be emailed and faxed to the site physician. If the site physician does not receive a receipt within 24hrs of them sending the report (during office hours), they should re-send the SAE Report Form to the PC-CTU by email or fax and telephone ahead.

The documentation will be reviewed by the Trial Management Team and the 'SAE Checklist' will be completed and retained by the PC-CTU. Following the initial check of the report, any additional information will be requested, and the CI or their medically qualified designated representative will review and evaluate the report for seriousness, causality and expectedness. In the event of a SUSAR the reporting timelines stated below will be followed. If there have been two assessments of causality made, the site physician's assessment cannot be downgraded. Where there is a discrepancy the worst case assessment is used for reporting purposes. The PC-CTU will also ensure that SAE reports are reviewed by the DMEC, at meetings held every 6 months. This arrangement will be reviewed by the DMEC prior to, and during the trial, depending on the expected and observed rate of SAEs.

Additional information, as it becomes available, will also be reported on the paper SAE Report Form (i.e. updating the original form) and returned to the PC-CTU by email or fax as above. The SAE Report Form will be filed in the Trial Master File according to PC-CTU SOP TM112 'Trial Master File and associated files', with copies filed in the patient's notes, the Case Record Form file and the Investigator Site File.

Trial Managers complete regular reports reviewed by the senior members of the PC-CTU. One of the metrics contained within this reporting is the number of SAEs reported and the cumulative number of SAEs for each study. Any concerns identified will be immediately raised with the Chief Investigator and may be tabled for discussion at the regular PC-CTU Management Committee meetings or referred to the study's DMEC for review. The DMEC also monitors the frequency and pattern of events reported as part of its independent oversight of the trial. The expectedness of adverse events occurring as a result of re-introduction of withdrawn medication will be determined according to the latest version of the Summary of medicinal Product Characteristics (SmPC, section 4.8). There are no sections of the SmPC, or previous clinical studies which detail expected adverse events as a result of medication withdrawal (the study IMP) and therefore all SAEs at least possibly related, and not as a result of re-introduction of withdrawn medication, will be considered unexpected and reported as SUSARs.

12.6. Reporting Procedures for SUSAR

All SUSARs will be reported by the CI to the relevant Competent Authority and to the REC and other parties as applicable. For fatal and life-threatening SUSARs, this will be done no later than 7 calendar days after the Sponsor or delegate is first aware of the reaction. Any additional relevant information will be reported within 8 calendar days of the initial report. All other SUSARs will be reported within 15 calendar days. Treatment codes will be un-blinded for specific participants.

Principal Investigators will be informed of all SUSARs for the relevant IMP for all studies with the same Sponsor, whether or not the event occurred in the current trial.

12.7. Data Monitoring and Ethics Committee

- A DMEC will be convened, including a GP/Geriatrician, statistician and consultant clinical pharmacologist. They will convene regularly prior to, during and following the trial, and will report to and advise the TSC and the TMG. The TSC will have independent chairs and 'stop guideline' authority to advise early termination of the trial in the event of safety concerns or futility such as poor recruitment rates. Together, the responsibilities of the DMEC and TSC committees are:
    - To safeguard the safety, rights and well-being of the trial participants.
    - To systematically monitor the trial data and review any analysis as outlined in the Statistical Analysis Plan or as requested by the TSC.
    - To evaluate the risk of the trial continuing and take appropriate action where necessary.
    - To consider data emerging from other related studies and its potential impact on the trial, if requested by the TSC.
    - To pick up any trends, such as increases in un/expected events, and take appropriate action.
    - To seek additional advice or information from investigators where required.
    - To act or advise, through the Chairman or other consultant, on incidents occurring between meetings that require rapid assessment.

12.8. Development Safety Update Reports

In addition to the expedited reporting above, the CI shall submit a Developmental Safety Update Report to the Competent Authority (the MHRA), Ethics Committee, Host NHS Trust and sponsor in line with PC-CTU SOP TM119 "Pharmacovigilance". This report will be submitted once a year throughout the trial within 60 days of the date of the anniversary of the CTA, or on request.

13. STATISTICS

A Statistical Analysis Plan for all analyses to be conducted will be produced separately. Below is a brief summary of the main proposed analyses. Qualitative and cost-effectiveness analyses are described in sections 10 and 11 respectively.

13.1. Description of Statistical Methods

The primary and secondary analyses will be by ITT, unless explicitly stated otherwise. The primary analysis will be a non-inferiority analysis by means of the "two one-sided test" (TOST) procedure,[53] whereby the $(1 - 2\alpha) \times 100\%$ confidence interval for the relative risk of participants with systolic blood pressure at 12 weeks below 150mmHg between the medication reduction group and the usual care group is calculated. Therefore, for $\alpha = 0.025$ the 95% confidence interval will be calculated. If the lower limit of the confidence interval is more than 0.9 (equal to a risk difference of 10%) then the research

hypothesis that medication reduction will by non-inferior in terms of blood pressure control to usual care will be accepted.

The relative risk and its confidence interval will be obtained by means of a generalised linear mixed effects model specifying a binomial distribution with a log link function. The response will be binary indicator of whether the person has a systolic blood pressure below 150mmHg at 12 weeks. Practice will be included in the model as a random effect. Adjustment will be made for baseline blood pressure by including it as a fixed effect. In addition, covariates found to be predictive of missingness will be included in the model.

As a secondary analysis of the primary outcome, a per-protocol (PP) analysis will be performed. The purpose of this analysis to support the non-inferiority research hypothesis, as an ITT analysis can be anticonservative for a non-inferiority hypothesis.[53] Participants who received the medication reduction intervention in the PP analysis will be defined as a participant in the medication reduction arm who maintained their medication reduction throughout the 12 week follow-up period. Accepting the research hypothesis for both ITT and PP analyses will lend strength to the conclusions of the study. If the PP analysis leads to a different conclusion, then the reasons for non-compliance of participants who did not follow the medication reduction intervention will be investigated to explain the discrepancy. To support this investigation, as a secondary analysis the proportion of participants in the medication reduction arm who maintained their medication reduction throughout the 12 week follow-up period will be reported.

The difference between the intervention and usual care of the changes in the following secondary outcomes will be analysed by means of linear mixed effects model, adjusting for the baseline level of the outcome and baseline systolic blood pressure and including practice as a random effect: systolic blood pressure, EQ-5D-5L and the Frailty index/frail scale. The difference in the rate of side effects and adverse events between the medication reduction and usual care arms will be analysed by means of a logistic mixed effects model adjusting for baseline systolic blood pressure and including practice as a random effect. Exploratory subgroup analyses of blood pressure control, change in blood pressure and maintenance of medication reduction will be conducted by different levels of baseline frailty, functional independence, cognitive function, number of medications prescribed at baseline and number of co-morbidities at baseline.

A sensitivity analysis will be conducted where participants whose BP was measured at home will be excluded from the analysis, as well as an analysis where the BP measurements are imputed for these participants. The results of these two sensitivity analyses will be compared to the primary analysis to examine whether the place of measurement affects the primary outcome.

13.1.1. Long term follow-up

A Statistical Analysis Plan for the long term follow-up will be prepared to provide further details of the different analyses proposed. All models proposed will be tested for their assumptions and will use alternative statistical methods if the assumptions of the proposed model failed.

Long-term follow-up objectives and outcomes are listed in Appendix H. Time-to-event outcomes from randomisation will be analysed using a mixed effects Cox proportional hazards model, adjusting for randomised group, baseline systolic blood pressure and including practice as a random effect. These time-to-event long-term primary and secondary outcomes are: all-cause hospitalisation or death, emergency hospitalisation (where this is as an in-patient), all-cause death, hospitalisation or death with: cardiovascular disease (defined as myocardial infarction, stroke, or heart failure); stroke; myocardial infarction, hospitalisation due to: falls; acute kidney injury; syncope; hypotension; fracture; electrolyte abnormalities, a diagnosis of dementia. Where more than one of any one of these events is plausible, as

far as possible, these primary and secondary outcomes will also be analysed as counts of events by means of a generalised linear Poisson mixed effects model, adjusting for randomised group, baseline systolic blood pressure and including practice as a random effect. However, if problem of over dispersion occurred or where event counts are considered low, these outcomes will be analysed as binary outcomes instead, using generalised linear logistic regression mixed effects models, adjusting for randomised group, baseline systolic blood pressure and including practice as a random effect. For outcomes with very low event rate where covariate adjustment is not possible, then unadjusted analysis will be performed.

Three year outcomes related to systolic blood pressure control and change in blood pressure and maintenance of medication reduction will be analysed using the same analytical models used to examine these outcomes at 12 week follow-up (see section 13.1). The difference between the intervention and usual care arms for the count of primary care consultations relating to hypertension (reported by staff type) will be analysed by means of a generalised linear Poisson mixed effects model, adjusting for randomised group, baseline systolic blood pressure and including practice as a random effect.

The difference between the intervention and usual care arms for the change in antihypertensive medication prescription (from baseline) will be analysed by means of a generalised linear mixed effects Poisson model, adjusting for randomised group, baseline systolic blood pressure, baseline antihypertensive medication prescription and including practice as a random effect.

The difference between the intervention and usual care arms for the number of prescribed medications at 3 year follow-up will be analysed by means of linear mixed effects model, adjusting for randomised group, baseline systolic blood pressure and including practice as a random effect.

Exploratory analyses of rates of all-cause hospitalisation or death, systolic blood pressure change and systolic blood pressure control will be conducted by different levels of baseline frailty, baseline functional independence, baseline cognitive function, number of antihypertensive medications prescribed at baseline, number of co-morbidities at baseline.

13.2. The Number of Participants

Assuming that 100% of patients in the usual care group, and 96% of those in the medication reduction group have controlled systolic blood pressure levels (<150mmHg) at follow-up, approximately 540 patients would be required to detect a non-inferior difference in systolic blood pressure control between groups. Calculations assume a 10% non-inferiority margin, 90% power, alpha of 2.5%, 10% loss to follow-up and a 10% dilution effect due to cross-over between arms. There is no existing precedent for an appropriate margin of non-inferiority in a trial of this nature and the paucity of existing literature on the topic makes one difficult to model. The margin of 10% has been chosen to inform future doctor-patient discussions about medication reduction: if the non-inferiority margin is met, it will suggest that for every ten patients who have their medication reduced, nine will still have controlled blood pressure at 12 weeks follow-up.

Based on previous data from Primary Care,[11] approximately 92 patients would be eligible for this study per practice recruited (average sized [n=7,000]). Assuming a conservative recruitment rate of 15%, we would require approximately 39 practices (13 from each of three centres: Oxford, Cambridge, Southampton), each randomising 14 patients to the study.

Recruitment will continue until 540 participants have been randomised and then all patients who have already been booked in for consent visits will also be seen and randomised, if eligible.

13.3. The Level of Statistical Significance

For the non-inferiority analysis, the two one sided test procedure will be used with the level of significance set at 2.5%. For all other analyses, the level of significance will be 5% two-sided significant level. P-values will be adjusted for any multiple comparisons in order to maintain an overall type I error rate of 5%.

13.4. Criteria for the Termination of the Trial

The trial is of a method of management through medication reduction, rather than a specific medicinal product. It is not anticipated that the trial will be terminated unless on the advice of the DMEC in the case of a series of Suspected Unexpected Serious Adverse Reactions (SUSARs). No statistical interim analysis is planned for the main trial.

13.5. Procedure for Accounting for Missing, Unused, and Spurious Data.

Missing data will be reported with reasons given where available, and the missing data pattern will be examined. We will explore the mechanism of missing data by means of logistic regression models which will explore if missingness (i.e. whether the primary outcome is missing or not) is related to measured baseline variables. Covariates found to be predictive of missingness will, where appropriate, be included as a covariate in the analysis model.

13.6. Inclusion in Analysis

All data will be included in the analysis as far as possible to allow full ITT analysis, though there will inevitably be the problem of missing data due to withdrawal, loss to follow-up, or non-response questionnaire items. For the PP analysis, all participants will be included in the analysis, but those participants randomised to the medication reduction arm will be assigned to the control arm if they failed to maintain their medication reduction throughout the 12 week follow-up period.

13.7. Procedures for Reporting any Deviation(s) from the Original Statistical Plan

The final statistical plan will be agreed prior to final data lock and prior to any analyses taking place. Any deviation thereafter will be reported in the final trial report.

14. DATA MANAGEMENT

14.1. Source Data

Source documents are where data are first recorded, and from which participants' CRF data are obtained. These include, but are not limited to, Primary Care and hospital records (from which medical history and previous and concurrent medication may be summarised into the CRF), clinical and office charts, pharmacy records, diaries, and correspondence.

CRF entries will be considered source data if the CRF is the site of the original recording (e.g. there is no other written or electronic record of data; *e.g.* baseline clinic blood pressure measurements). All

documents will be stored safely in confidential conditions. On all trial-specific documents, other than the signed consent, the participant will be referred to by the trial participant number/code, not by name.

14.2. Access to Data

Direct access will be granted to authorised representatives from the Sponsor, host institution (University of Oxford OPTiMISE research team) and the regulatory authorities to permit trial-related monitoring, audits and inspections. To ensure data transparency, the trial has been registered on the EU Clinical Trials Register (EudraCT) and will be registered on the International Standard Randomised Controlled Trial Number (ISRCTN) registry before the first participant is recruited.

14.3. Data Handling and Record Keeping

All trial data (expect specific questionnaires not validated for electronic data capture) will be entered on to electronic CRFs which will link directly to the trial database. This clinical database will be built and managed by the PC-CTU in line with the PC-CTU SOPs and will hold and allow data management of all data points required to conduct the final analysis. The clinical database will be built on an externally validated secure web-based platform allowing for data tracking by use of date stamped audit logs. Within this database, participants will be identified only by a unique study ID to offer patient confidentiality and protect against bias. A separate database will be used to securely store identifiable patient information required to contact patients and permit long term follow-up in the future. Access to these data will be strictly on a need to know basis. The identifiers will be held separately from the CRFs collecting clinical data. The unique study identifier will generated for every patient enrolled to the study and this will be entered onto both study databases to permit linkage of identifiable and anonymised clinical data where necessary. Double data entry will be employed for entry of the unique study identifier onto both databases to ensure accuracy. Each database will include secure login for staff at participating sites and facilities for manual entry of data and upload of files where appropriate. A clinical data manager will be assigned to the study supervised by Oxford PC-CTU's Senior Clinical Data Specialist and PC-CTU SOPs will be followed.

15. QUALITY ASSURANCE PROCEDURES

The trial will be conducted in accordance with the current approved protocol, GCP, relevant regulations and PC-CTU standard operating procedures. The PC-CTU has in place procedures for assessing risk management for trials which will outline the monitoring required. The investigators and all trial related site staff will receive appropriate training in Good Clinical Practice and trial procedures.

Regular monitoring will be performed according to GCP. Data will be evaluated for compliance with the protocol and accuracy in relation to source documents. Following written standard operating procedures, the monitors will verify that the clinical trial is conducted and data are generated, documented and reported in compliance with the protocol, GCP and the applicable regulatory requirements.

The PC-CTU Trial Management Group will be responsible for the monitoring of all aspects of the trial's conduct and progress and will ensure that the protocol is adhered to and that appropriate action is taken to safeguard participants and the quality of the trial itself. The TMG will be comprised of individuals responsible for the trial's day to day management (e.g the CI, trial manager, statistician, data manager) and will meet regularly throughout the course of the trial.

A TSC will be convened at 6 month intervals to provide overall supervision of the trial and ensure its conduct is in accordance with the principles of GCP and the relevant regulations. The role of a TSC is to provide overall supervision of the trial and ensure that it is being conducted in accordance with the principles of GCP and the relevant regulations. The TSC will agree the trial protocol and any protocol amendments and provide advice to the investigators on all aspects of the trial. The TSC will consist of members who are independent of the investigators, in particular an independent chairperson.

An independent DMEC meets at 6 monthly intervals before, and until the end of the trial. They will review the accruing trial and safety data to ensure trial site staff and participants are aware of any relevant safety information and to determine whether any reasons exist for the trial to be discontinued (See section 12.6).

16. SERIOUS BREACHES

The Medicines for Human Use (Clinical Trials) Regulations, PC-CTU SOP TM125 “Trial Related Deviations and Serious Breaches” contains a requirement for the notification of "serious breaches" to the MHRA within 7 days of the Sponsor becoming aware of the breach.

A serious breach is defined as “A breach of GCP or the trial protocol which is likely to affect to a significant degree:

(a) the safety or physical or mental integrity of the subjects of the trial; or
(b) the scientific value of the trial”.

In the event that a serious breach is suspected the Sponsor must be contacted within 1 working day. In collaboration with the C.I., the serious breach will be reviewed by the Sponsor and, if appropriate, the Sponsor will report it to the REC committee, Regulatory authority and the NHS host organisation within seven calendar days.

17. ETHICAL AND REGULATORY CONSIDERATIONS

17.1. Declaration of Helsinki

The Investigator will ensure that this trial is conducted in accordance with the principles of the Declaration of Helsinki.

17.2. Guidelines for Good Clinical Practice

The Investigator will ensure that this trial is conducted in accordance with relevant regulations and with Good Clinical Practice.

17.3. Approvals

The protocol, informed consent form, participant information sheet and any proposed advertising material will be submitted to an appropriate Research Ethics Committee (REC), regulatory authorities (MHRA in the UK), host institution(s) and HRA for written approval. The Investigator will submit and, where necessary, obtain approval from the above parties for all substantial amendments to the original approved documents.

17.4. Reporting

The CI shall submit once a year throughout the clinical trial, or on request, an Annual Progress Report to the REC, host organisation and Sponsor. In addition, an End of Trial notification and final report will be submitted to the MHRA, the REC, host organisation and Sponsor.

17.5. Participant Confidentiality

The trial staff will ensure that the participants' anonymity is maintained. The participants will be identified only by a participant ID number on all trial documents and in the electronic clinical database. All data will be stored securely on an electronic study database and will comply with the General Data Protection Regulation (GDPR) and Data Protection Act 2018, which require data to be de-identified as soon as it is practical to do so. The study database will be managed according to Standard Operating Procedures maintained by the PC CTU. Access rights to data and applications software will be clearly defined and staff authorised to access personal data will be formally notified in writing of the permissible scope of their access. Data access will be limited to specific members of the research team (trained in data protection policy) including the chief investigator (as study guarantor), data manager and database programmer. For each database application, system users will be given a valid user system account name (username ID), and a password known only to that user to prevent unauthorised use of systems. All data will be entered into the database through a reliably encrypted gateway.

Confidentiality of potential participants in the programme will be maintained by making the initial searches of the practice computer systems and subsequent study invitations the responsibility of the practice. All data held in paper form (e.g. consent forms) will be kept in locked filing cabinets and will only be accessible by trial staff and authorised personnel.

17.6. Expenses and Benefits

Reasonable travel expenses for any visits additional to normal care will be reimbursed on production of receipts, or a mileage allowance provided as appropriate. For patients with limited mobility and no access to their own form of transport, pre-paid taxis will be offered to ensure that accessibility doesn't prevent them from being able to participate. Patients in the intervention arm of the study will be provided with clinically validated BP monitoring equipment during the trial.

17.7. Other Ethical Considerations

This research involves older participants, some of whom may be considered vulnerable. This is necessary since it is these frail, vulnerable populations who could potentially gain the most from antihypertensive medication reduction. Great care will be taken to ensure all potential participants have the trial clearly explained, and are given sufficient time to decide whether to give informed consent. This will include provision of simplified, patient information sheets with large fonts, video infographics to explain the study to those who find it difficult to read and extended GP consultation periods for explaining the study and taking informed consent.

We do not anticipate any other ethical considerations, other than those outlined above.

18. FINANCE AND INSURANCE

18.1. Funding

This trial is funded by the National Institute for Health Research (NIHR) Oxford Collaborations for Leadership in Applied Research and Care (CLARHC) and the NIHR School for Primary Care Research (SPCR). The long term follow-up has been funded by the British Heart Foundation.

18.2. Insurance

The University has a specialist insurance policy in place which would operate in the event of any participant suffering harm as a result of their involvement in the research (Newline Underwriting Management Ltd, at Lloyd's of London). NHS indemnity operates in respect of the clinical treatment that is provided.

19. PUBLICATION POLICY

The Investigators will be involved in reviewing drafts of the manuscripts, abstracts, press releases and any other publications arising from the study. Authors will acknowledge that the study was funded by the NIHR Oxford CLARHC and the NIHR SPCR. Authorship will be determined in accordance with the ICMJE guidelines and other contributors will be acknowledged.

All research outputs from this work will be published in peer-reviewed journals. Study findings will be presented at regional, national and international conferences to ensure maximum dissemination amongst academic and clinical colleagues. Where possible, local and national media will be engaged to bring the research findings to a wider audience. We will also use social media (e.g. Twitter, blogs) to disseminate the progress and findings to a wider audience. 'Patient friendly' study summary documents and infographics will be made available to all participants at the end of the trial via the study website and distributed to relevant patient groups (e.g. British Heart Foundation, Age UK), ensuring widespread dissemination amongst service users. Regular trial updates and final results will be further disseminated using the communication structures developed by the NIHR Oxford CLAHRC and the SPCR (website, newsletters, symposia, etc.).

It is anticipated that the findings of this trial will support better patient-centred management plans for the prevention of cardiovascular disease in older individuals and will be made available for the next iterations of the NICE hypertension and multi-morbidity guidelines.

20. REFERENCES

1. SPRINT Investigators. A Randomized Trial of Intensive versus Standard Blood-Pressure Control. *New England Journal of Medicine* 2015; **373**(22): 2103-16.

2. Beckett NS, Peters R, Fletcher AE, et al. Treatment of hypertension in patients 80 years of age or older. *New England Journal of Medicine* 2008; **358**(18): 1887-98.

3. Cushman WC, Evans GW, Byington RP, et al. Effects of intensive blood-pressure control in type 2 diabetes mellitus. *The New England journal of medicine* 2010; **362**(17): 1575-85.

4. Office for National Statistics. Mid-year populations estimates: Aging, fastest increase in the 'oldest old'. http://www.ons.gov.uk, 2010.

5. Barnett K, Mercer SW, Norbury M, Watt G, Wyke S, Guthrie B. Epidemiology of multimorbidity and implications for health care, research, and medical education: a cross-sectional study. *Lancet* 2012; **380**(9836): 37-43.

6. Payne RA, Avery AJ, Duerden M, Saunders CL, Simpson CR, Abel GA. Prevalence of polypharmacy in a Scottish primary care population. *European journal of clinical pharmacology* 2014; **70**(5): 575-81.

7. Bradley MC, Motterlini N, Padmanabhan S, et al. Potentially inappropriate prescribing among older people in the United Kingdom. *BMC geriatrics* 2014; **14**: 72.

8. Sato I, Akazawa M. Polypharmacy and adverse drug reactions in Japanese elderly taking antihypertensives: a retrospective database study. *Drug, healthcare and patient safety* 2013; **5**: 143-50.

9. Opondo D, Eslami S, Visscher S, et al. Inappropriateness of medication prescriptions to elderly patients in the primary care setting: a systematic review. *PloS one* 2012; **7**(8): e43617.

10. Violan C, Foguet-Boreu Q, Roso-Llorach A, et al. Burden of multimorbidity, socioeconomic status and use of health services across stages of life in urban areas: a cross-sectional study. *BMC public health* 2014; **14**: 530.

11. Sheppard JP, Singh S, Fletcher K, McManus RJ, Mant J. Impact of age and sex on primary preventive treatment for cardiovascular disease in the West Midlands, UK: cross sectional study. *BMJ;* 2012; **345**: e4535.

12. Bejan-Angoulvant T, Saadatian-Elahi M, Wright JM, et al. Treatment of hypertension in patients 80 years and older: the lower the better? A meta-analysis of randomized controlled trials. *Journal of hypertension* 2010; **28**(7): 1366-72.

13. Benetos A, Labat C, Rossignol P, et al. Treatment With Multiple Blood Pressure Medications, Achieved Blood Pressure, and Mortality in Older Nursing Home Residents: The PARTAGE Study. *JAMA internal medicine* 2015; **175**(6): 989-95.

14. Tinetti ME, Han L, Lee DS, et al. Antihypertensive medications and serious fall injuries in a nationally representative sample of older adults. *JAMA internal medicine* 2014; **174**(4): 588-95.

15. Fried TR, Tinetti ME, Towle V, O'Leary JR, Iannone L. Effects of benefits and harms on older persons' willingness to take medication for primary cardiovascular prevention. *Archives of internal medicine* 2011; **171**(10): 923-8.

16. Marshall IJ, Wolfe CD, McKevitt C. Lay perspectives on hypertension and drug adherence: systematic review of qualitative research. *BMJ (Clinical research ed)* 2012; **345**: e3953.

17. Schuling J, Gebben H, Veehof LJ, Haaijer-Ruskamp FM. Deprescribing medication in very elderly patients with multimorbidity: the view of Dutch GPs. A qualitative study. *BMC family practice* 2012; **13**: 56.

18. Scott IA, Anderson K, Freeman CR, Stowasser DA. First do no harm: a real need to deprescribe in older patients. *The Medical journal of Australia* 2014; **201**(7): 390-2.

19. Pajewski NM, Williamson JD, Applegate WB, et al. Characterizing Frailty Status in the Systolic Blood Pressure Intervention Trial. *The journals of gerontology Series A, Biological sciences and medical sciences* 2016; **71**(5): 649-55.

20. Kulminski A, Yashin A, Ukraintseva S, et al. Accumulation of health disorders as a systemic measure of aging: Findings from the NLTCS data. *Mechanisms of ageing and development* 2006; **127**(11): 840-8.

21. Romero-Ortuno R, Kenny RA. The frailty index in Europeans: association with age and mortality. *Age and ageing* 2012; **41**(5): 684-9.

22. Iyer S, Naganathan V, McLachlan AJ, Le Couteur DG. Medication withdrawal trials in people aged 65 years and older: a systematic review. *Drugs & aging* 2008; **25**(12): 1021-31.

23. Moonen JE, Foster-Dingley JC, de Ruijter W, et al. Effect of Discontinuation of Antihypertensive Treatment in Elderly People on Cognitive Functioning--the DANTE Study Leiden: A Randomized Clinical Trial. *JAMA internal medicine* 2015; **175**(10): 1622-30.

24. Correa Leite ML, Firmo JO, Loyola Filho AI, Lima-Costa MF. Discontinuation of anti-hypertensive drugs increases 11-year cardiovascular mortality risk in community-dwelling elderly (the Bambui Cohort Study of Ageing). *BMC public health* 2014; **14**: 725.

25. Chapman RH, Benner JS, Petrilla AA, et al. Predictors of adherence with antihypertensive and lipid-lowering therapy. *Archives of internal medicine* 2005; **165**(10): 1147-52.

26. Chapman RH, Petrilla AA, Benner JS, Schwartz JS, Tang SS. Predictors of adherence to concomitant antihypertensive and lipid-lowering medications in older adults: a retrospective, cohort study. *Drugs & aging* 2008; **25**(10): 885-92.

27. Califf RM, Alexander KP. Cardiovascular research in the elderly: challenges, opportunities, and impact. *Postgraduate medicine* 2002; **112**(6 Suppl Heart): 6-11.

28. Mant J, Hobbs FD, Fletcher K, et al. Warfarin versus aspirin for stroke prevention in an elderly community population with atrial fibrillation (the Birmingham Atrial Fibrillation Treatment of the Aged Study, BAFTA): a randomised controlled trial. *Lancet* 2007; **370**(9586): 493-503.

29. Tolmie EP, Mungall MM, Louden G, Lindsay GM, Gaw A. Understanding why older people participate in clinical trials: the experience of the Scottish PROSPER participants. *Age and ageing* 2004; **33**(4): 374-8.

30. Fletcher B, Gheorghe A, Moore D, Wilson S, Damery S. Improving the recruitment activity of clinicians in randomised controlled trials: a systematic review. *BMJ open* 2012; **2**(1): e000496.

31. Clegg A, Bates C, Young J, et al. Development and validation of an electronic frailty index using routine primary care electronic health record data. *Age and ageing* 2016; **45**(3): 353-60.

32. Antoniou EE, Draper H, Reed K, Burls A, Southwood TR, Zeegers MP. An empirical study on the preferred size of the participant information sheet in research. *Journal of medical ethics* 2011; **37**(9): 557-62.

33. Mattu GS, Heran BS, Wright JM. Overall accuracy of the BpTRU--an automated electronic blood pressure device. *Blood pressure monitoring* 2004; **9**(1): 47-52.

34. Lahrmann H, Cortelli P, Hilz M, Mathias CJ, Struhal W, Tassinari M. EFNS guidelines on the diagnosis and management of orthostatic hypotension. *European journal of neurology* 2006; **13**(9): 930-6.

35. Herdman M, Gudex C, Lloyd A, et al. Development and preliminary testing of the new five-level version of EQ-5D (EQ-5D-5L). *Quality of life research : an international journal of quality of life aspects of treatment, care and rehabilitation* 2011; **20**(10): 1727-36.

36. Morley JE, Malmstrom TK, Miller DK. A simple frailty questionnaire (FRAIL) predicts outcomes in middle aged African Americans. *The journal of nutrition, health & aging* 2012; **16**(7): 601-8.

37. Sulter G, Steen C, De Keyser J. Use of the Barthel index and modified Rankin scale in acute stroke trials. *Stroke; a journal of cerebral circulation* 1999; **30**(8): 1538-41.

38. Mitnitski AB, Mogilner AJ, Rockwood K. Accumulation of deficits as a proxy measure of aging. *TheScientificWorldJournal* 2001; **1**: 323-36.

39. Rockwood K, Song X, MacKnight C, et al. A global clinical measure of fitness and frailty in elderly people. *CMAJ : Canadian Medical Association journal = journal de l'Association medicale canadienne* 2005; **173**(5): 489-95.

40. Nasreddine ZS, Phillips NA, Bedirian V, et al. The Montreal Cognitive Assessment, MoCA: a brief screening tool for mild cognitive impairment. *Journal of the American Geriatrics Society* 2005; **53**(4): 695-9.

41. Horne R, Weinman J. Patients' beliefs about prescribed medicines and their role in adherence to treatment in chronic physical illness. *Journal of psychosomatic research* 1999; **47**(6): 555-67.

42. de Vries NM, Staal JB, van Ravensberg CD, Hobbelen JS, Olde Rikkert MG, Nijhuis-van der Sanden MW. Outcome instruments to measure frailty: a systematic review. *Ageing research reviews* 2011; **10**(1): 104-14.

43. Searle SD, Mitnitski A, Gahbauer EA, Gill TM, Rockwood K. A standard procedure for creating a frailty index. *BMC geriatrics* 2008; **8**: 24.

44. McManus RJ, Mant J, Haque MS, et al. Effect of self-monitoring and medication self-titration on systolic blood pressure in hypertensive patients at high risk of cardiovascular disease: the TASMIN-SR randomized clinical trial. *Jama* 2014; **312**(8): 799-808.

45. Gallagher P, Ryan C, Byrne S, Kennedy J, O'Mahony D. STOPP (Screening Tool of Older Person's Prescriptions) and START (Screening Tool to Alert doctors to Right Treatment). Consensus validation. *International journal of clinical pharmacology and therapeutics* 2008; **46**(2): 72-83.

46. National Clinical Guideline C. National Institute for Health and Clinical Excellence Guidance CG127. Hypertension: The Clinical Management of Primary Hypertension in Adults: Update of Clinical Guidelines 18 and 34. London: Royal College of Physicians (UK); 2011.

47. McManus RJ, Mant J, Bray EP, et al. Telemonitoring and self-management in the control of hypertension (TASMINH2): a randomised controlled trial. *Lancet* 2010; **376**(9736): 163-72.

48. Tucker KL, Sheppard JP, Stevens R, et al. Individual patient data meta-analysis of self-monitoring of blood pressure (BP-SMART). *Journal of Human Hypertension* 2015; **29**(10): 635-.

49. Nathan A, Goodyer L, Lovejoy A, Rashid A. 'Brown bag' medication reviews as a means of optimizing patients' use of medication and of identifying potential clinical problems. *Family practice* 1999; **16**(3): 278-82.

50. Donovan J, Mills N, Smith M, et al. Quality improvement report: Improving design and conduct of randomised trials by embedding them in qualitative research: ProtecT (prostate testing for cancer and treatment) study. Commentary: presenting unbiased information to patients can be difficult. *BMJ (Clinical research ed)* 2002; **325**(7367): 766-70.

51. Donovan JL, Rooshenas L, Jepson M, et al. Optimising recruitment and informed consent in randomised controlled trials: the development and implementation of the Quintet Recruitment Intervention (QRI). *Trials* 2016; **17**(1): 283.

52. Kaambwa B, Bryan S, Jowett S, et al. Telemonitoring and self-management in the control of hypertension (TASMINH2): a cost-effectiveness analysis. *European journal of preventive cardiology* 2014; **21**(12): 1517-30.

53. Walker E, Nowacki AS. Understanding equivalence and noninferiority testing. *Journal of general internal medicine* 2011; **26**(2): 192-6.

54. Bulpitt CJ, Beckett NS, Cooke J, et al. Results of the pilot study for the Hypertension in the Very Elderly Trial. *Journal of hypertension* 2003; **21**(12): 2409-17.

55. Warwick J, Falaschetti E, Rockwood K, et al. No evidence that frailty modifies the positive impact of antihypertensive treatment in very elderly people: an investigation of the impact of frailty upon treatment effect in the HYpertension in the Very Elderly Trial (HYVET) study, a double-blind, placebo-controlled study of antihypertensives in people with hypertension aged 80 and over. *BMC medicine* 2015; **13**: 78.

56. Statistics OfN. http://wwwonsgovuk; 2010.

57. Barnett K MS, Norbury M, Watt G, Wyke S, Guthrie B. Epidemiology of multimorbidity and implications for health care, research, and medical education: a cross-sectional study. *Lancet* 2012; **380**(9836): 37-43.

58. Payne RA AA, Duerden M, Saunders CL, Simpson CR, Abel GA. Prevalence of polypharmacy in a Scottish primary care population. *European journal of clinical pharmacology* 2014; **70**(5): 575-81.

59. Bradley MC MN, Padmanabhan S, Cahir C, Williams T, Fahey T, Hughes CM. Potentially inappropriate prescribing among older people in the United Kingdom. *BMC geriatrics* 2014; **14**: 72.

60. Sato I AM. Polypharmacy and adverse drug reactions in Japanese elderly taking antihypertensives: a retrospective database study. *Drug, healthcare and patient safety* 2013; **5**: 143-50.

61. Opondo D ES, Visscher S, de Rooij SE, Verheij R, Korevaar JC, Abu-Hanna A. Inappropriateness of medication prescriptions to elderly patients in the primary care setting: a systematic review. *PloS one* 2012; **7**(8): e43617.

62. Violan C F-BQ, Roso-Llorach A, Rodriguez-Blanco T, Pons-Vigues M, Pujol-Ribera E, Munoz-Perez MA, Valderas JM. Burden of multimorbidity, socioeconomic status and use of health services across stages of life in urban areas: a cross-sectional study. *BMC public health* 2014; **14**: 530.

63. Sheppard JP SS, Fletcher K, McManus RJ, Mant J. Impact of age and sex on primary preventive treatment for cardiovascular disease in the West Midlands, UK: cross sectional study. *BMJ (Clinical research ed)* 2012; **345**: e4535.

64. Beckett NS PR, Fletcher AE, Staessen JA, Liu L, Dumitrascu D, Stoyanovsky V, Antikainen RL, Nikitin Y, Anderson C, Belhani A, Forette F, Rajkumar C, Thijs L, Banya W, Bulpitt CJ. Treatment of hypertension in patients 80 years of age or older. *The New England journal of medicine* 2008; **358**(18): 1887-98.

65. Bejan-Angoulvant T S-EM, Wright JM, Schron EB, Lindholm LH, Fagard R, Staessen JA, Gueyffier F. Treatment of hypertension in patients 80 years and older: the lower the better? A meta-analysis of randomized controlled trials. *Journal of hypertension* 2010; **28**(7): 1366-72.

66. Benetos A LC, Rossignol P, Fay R, Rolland Y, Valbusa F, Salvi P, Zamboni M, Manckoundia P, Hanon O, Gautier S. Treatment With Multiple Blood Pressure Medications, Achieved Blood Pressure, and Mortality in Older Nursing Home Residents: The PARTAGE Study. *JAMA internal medicine* 2015.

67. Tinetti ME HL, Lee DS, McAvay GJ, Peduzzi P, Gross CP, Zhou B, Lin H. Antihypertensive medications and serious fall injuries in a nationally representative sample of older adults. *JAMA internal medicine* 2014; **174**(4): 588-95.

68. Fried TR TM, Towle V, O'Leary JR, Iannone L. Effects of benefits and harms on older persons' willingness to take medication for primary cardiovascular prevention. *Archives of internal medicine* 2011; **171**(10): 923-8.

69. Marshall IJ WC, McKevitt C. Lay perspectives on hypertension and drug adherence: systematic review of qualitative research. *BMJ (Clinical research ed)* 2012; **345**: e3953.

70. Schuling J GH, Veehof LJ, Haaijer-Ruskamp FM. Deprescribing medication in very elderly patients with multimorbidity: the view of Dutch GPs. A qualitative study. *BMC family practice* 2012; **13**: 56.

71. Scott IA AK, Freeman CR, Stowasser DA. First do no harm: a real need to deprescribe in older patients. *The Medical journal of Australia* 2014; **201**(7): 390-2.

72. Sheppard JP, Burt J, al MLe. Effect of Antihypertensive Medication Reduction vs Usual Care on Short-term Blood Pressure Control in Patients With Hypertension Aged 80 Years and Older The OPTIMISE Randomized Clinical Trial. 2020; **323**(20).

73. Thomas R Sullivan IRW, Amy B Salter, Philip Ryan, Katherine J Lee. Should multiple imputation be the method of choice for handling missing data in randomized trials? 2016; **27**(9).

21. APPENDIX A: TRIAL FLOW CHART

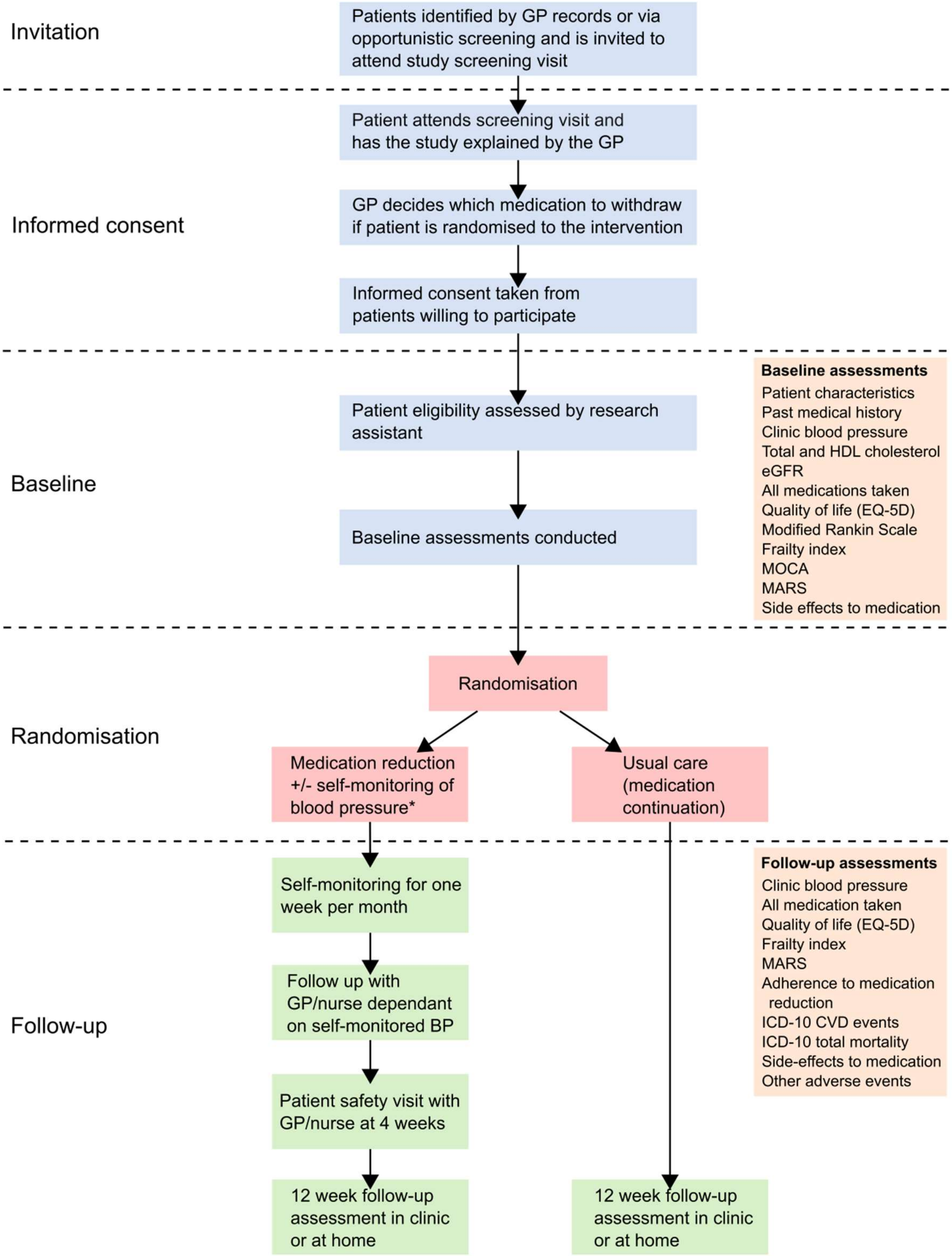

Please refer to Appendix H for Long Term follow-up details.

*Monitoring of blood pressure at home will be encouraged but those not willing or able will still be included in the trial. All patient will be asked to attend a safety monitoring visit with their GP/nurse/healthcare assistant/other appropriate, delegated healthcare professional four weeks after baseline.

GP = General practitioner; BP = Blood pressure; HDL = High density lipoprotein; ICD = International Statistical Classification of Diseases and Related Health Problems; CVD = Cardiovascular disease; eGFR = estimated Glomerular Filtration Rate (eGFR); MARS = Medication Adherence Rating Scale; MOCA = Montreal Cognitive Assessment

22. APPENDIX B: DATA COLLECTION SOURCES AND SCHEDULE

No.	Variable	From medical notes	Measured at clinic	Recorded at Baseline	Recorded at Follow-up
**1**	Age		✓	✓	
**2**	Sex		✓	✓	
**3**	Ethnicity		✓	✓	
**4**	Marital status		✓	✓	
**5**	Education		✓	✓	
**6**	Duration of hypertension	✓		✓	
**7**	Past medical history	✓		✓	
**8**	Alcohol consumption		✓	✓	✓
**9**	Smoking		✓	✓	✓
**10**	Height		✓	✓	✓
**11**	Weight		✓	✓	✓
**12**	Clinic blood pressure (sitting and standing)		✓	✓	✓
**13**	Cholesterol (total and HDL)	✓		✓	
**14**	estimated Glomerular Filtration Rate (eGFR)	✓		✓	
**15**	Prescribed or over the counter medications (all medications)*	✓	✓	✓	✓
**16**	Quality of life (according to EQ-5D-5L)[35]		✓	✓	✓
**17**	Functional independence (defined by modified Rankin Scale)[37]		✓	✓	
**18**	Frailty (according to the FRAIL scale)[36]		✓	✓	✓
**19**	Frailty (according to the frailty index and electronic frailty index)[31,39]	✓	✓	✓	✓
**20**	Cognitive function (defined by the Montreal Cognitive Assessment [MoCA])[40]		✓	✓	
**21**	Adherence to medication (according to the Medication Adherence Rating Scale (MARS) Questionnaire)[41]		✓	✓	✓
**22**	Adherence to medication reduction		✓		✓
**23**	ICD-10 coded Cardiovascular events and mortality during the trial	✓			✓
**24**	Recording of potential side effects to medication		✓	✓	✓
**25**	Recording of adverse events	✓	✓		✓

HDL = High density lipoprotein; ICD = International Statistical Classification of Diseases and Related Health Problems

*Drug substance/name, formulation, dose, frequency, start date and adherence over past 12 months (according to clinical system)

Please refer to Appendix H for details of outcome data collected for Long Term follow-up.

23. APPENDIX C: ITEMS INCLUDED IN FRAILTY INDEX ASSESSMENT

Adapted from Searle *et al.,* and Clegg *et al.* (the original Frailty Index and electronic Frailty Index),[31,43] Morley *et al.* (the FRAIL Scale),[36] the HYVET[54,55] and OPTIMED trials. Items permit estimation of frailty according to the original frailty index (FI; for comparison with SPRINT and HYVET trials),[43] the electronic frailty index (eFI)[31] and the frail scale (FS).[36]

No.	Item	Source	Deficit type	Coding	Routine data	Patient data	FI	eFI	FS
1.	Activities prevented by pain/discomfort	OPTIMED	Symptom	Yes (1), No (0)	✓		✓	✓	
2.	Alzheimer's Disease or other dementia	OPTIMED	Disease	Yes (1), No (0)	✓		✓		
3.	Angina	Morley	Disease	Yes (1), No (0)	✓		✓	✓	✓
4.	Any fall in the past month	New	Symptom	Yes (1), No (0)		✓	✓		
5.	Arthritis or rheumatism	Searle et al.,	Disease	Yes (1), No (0)	✓		✓	✓	✓
6.	Asthma	Morley	Disease	Yes (1), No (0)	✓		✓		✓
7.	Atrial Fibrillation	Clegg et al.,	Disease	Yes (1), No (0)	✓		✓	✓	
8.	Autoimmune disease	OPTIMED	Disease	Yes (1), No (0)	✓		✓		
9.	Back pain (excluding arthritis)	OPTIMED	Symptom	Yes (1), No (0)	✓		✓		
10.	Bowel disorder including faecal incontinence	OPTIMED	Disease	Yes (1), No (0)	✓		✓		
11.	Cancer	Searle et al.,	Disease	Yes (1), No (0)	✓		✓		✓
12.	Chronic Kidney disease	Morley	Disease	Yes (1), No (0)	✓		✓	✓	✓
13.	Chronic lung disease	Searle et al.,	Disease	Yes (1), No (0)	✓		✓	✓	✓
14.	Cognition problems (but no dementia diagnosed)	OPTIMED	Disability	Yes (1), No (0)	✓		✓	✓	
15.	Derived trouble with vision	OPTIMED	Disability	Yes (1), No (0)	✓		✓	✓	
16.	Dexterity problems	OPTIMED	Disability	Yes (1), No (0)	✓		✓		
17.	Diabetes	Searle et al.,	Disease	Yes (1), No (0)	✓		✓	✓	✓
18.	Difficulty walking 10 steps without aids or resting (resistance)	Morley	Disability	Yes (1), No (0)		✓	✓		✓
19.	Difficulty walking 100 yards without aids (ambulation)	Morley	Disability	Yes (1), No (0)		✓	✓		✓
20.	Dizziness	Clegg et al.,	Symptom	Yes (1), No (0)	✓		✓	✓	

21.	Dyspnoea	Clegg et al.,	Symptom	Yes (1), No (0)	✓		✓	✓	
22.	Emotional problems	OPTIMED	Disability	Yes (1), No (0)	✓		✓		
23.	Epilepsy	OPTIMED	Disease	Yes (1), No (0)	✓		✓		
24.	Fall resulting in hospitalisation	New	Symptom	Yes (1), No (0)	✓		✓	✓	
25.	Feeling depressed	Searle et al.,	Symptom	Most of the time (1), sometimes (0.5), rarely (0)		✓	✓		
26.	Feeling lonely	Searle et al.,	Symptom	Most of the time (1), sometimes (0.5), rarely (0)		✓	✓		
27.	Feeling tired a lot of the time (fatigue)	Morley	Symptom	1 = All of the time, 0.75 = Most of the time, 0.50 = Some of the time, 0.25 = A little of the time, 0 = None of the time		✓	✓		✓
28.	Foot problems	Clegg et al.,	Disease	Yes (1), No (0)	✓		✓	✓	
29.	Fragility fracture	Clegg et al.,	Disease	Yes (1), No (0)	✓		✓	✓	
30.	Haematological disorders (anaemia, CML etc.)	OPTIMED	Disease	Yes (1), No (0)	✓		✓	✓	
31.	Hearing problems	OPTIMED	Disability	Yes (1), No (0)	✓		✓	✓	
32.	Heart failure	Searle et al.,	Disease	Yes (1), No (0)	✓		✓	✓	✓
33.	Heart valve disease	Clegg et al.,	Disease	Yes (1), No (0)	✓		✓	✓	
34.	High BP or hypertension or treated BP	Searle et al.,	Disease	Yes (1), No (0)	✓		✓	✓	✓
35.	Housebound	Clegg et al.,	Disability	Yes (1), No (0)	✓		✓	✓	
36.	Hypotension/syncope	Clegg et al.,	Disease	Yes (1), No (0)	✓		✓	✓	
37.	Loss of weight in the past year	Morley	Symptom	≥5% (1), <5% (0)	✓		✓	✓	✓
38.	Mobility problems	OPTIMED	Disability	Yes (1), No (0)	✓		✓	✓	
39.	Needing help bathing	Searle et al.,	Disability	Yes (1), No (0)		✓	✓		
40.	Needing help for housework	Searle et al.,	Symptom	Yes (1), No (0)		✓	✓		
41.	Needing help getting in and out of a chair	Searle et al.,	Disability	Yes (1), No (0)		✓	✓		
42.	Needing help in moving about the house	Searle et al.,	Symptom	Yes (1), No (0)		✓	✓		
43.	Needing help taking medication	Searle et al.,	Symptom	Yes (1), No (0)		✓	✓		
44.	Orthostatic Hypertension	HYVET	Symptom	Yes (1), No (0)	✓		✓		
45.	Osteoporosis	Clegg et al.,	Disease	Yes (1), No (0)	✓		✓	✓	

46.	Overwright or obese	HYVET	Symptom	BMI <25 (0), ≥25 but <30 (0.5), ≥30 (1)	✓		✓		
47.	Parkinsonism and tremor	Clegg et al.,	Disease	Yes (1), No (0)	✓			✓	
48.	Peripheral vascular disease	Clegg et al.,	Disease	Yes (1), No (0)	✓			✓	
49.	Polypharmacy	Clegg et al.,	Sign	Yes (1), No (0)	✓			✓	
50.	Previous Myocardial Infarction	Searle et al.,	Disease	Yes (1), No (0)	✓		✓		✓
51.	Previous stroke	Searle et al.,	Disease	Yes (1), No (0)	✓		✓	✓	✓
52.	Receiving home care services	OPTIMED	Symptom	Yes (1), No (0)	✓	✓	✓	✓	
53.	Self-rating of Health	Searle et al.,	Symptom	Poor (1), Fair (0.75), Good (0.5), Very Good (0.25), Excellent (0)		✓	✓		
54.	Skin ulcers	OPTIMED	Disease	Yes (1), No (0)	✓		✓	✓	
55.	Sleep disturbance	Clegg et al.,	Sign	Yes (1), No (0)	✓			✓	
56.	Social vulnerability	Clegg et al.,	Disability	Yes (1), No (0)	✓			✓	
57.	Stomach or intestinal ulcers	OPTIMED	Disease	Yes (1), No (0)	✓		✓	✓	
58.	Thyroid condition or treatment	OPTIMED	Disease	Yes (1), No (0)	✓		✓	✓	
59.	Urinary incontinence	HYVET	Symptom	Yes (1), No (0)	✓		✓	✓	
60.	Urinary system disease	Clegg et al.,	Disease	Yes (1), No (0)	✓			✓	

24. APPENDIX D: MEDICATION REDUCTION ALGORITHM

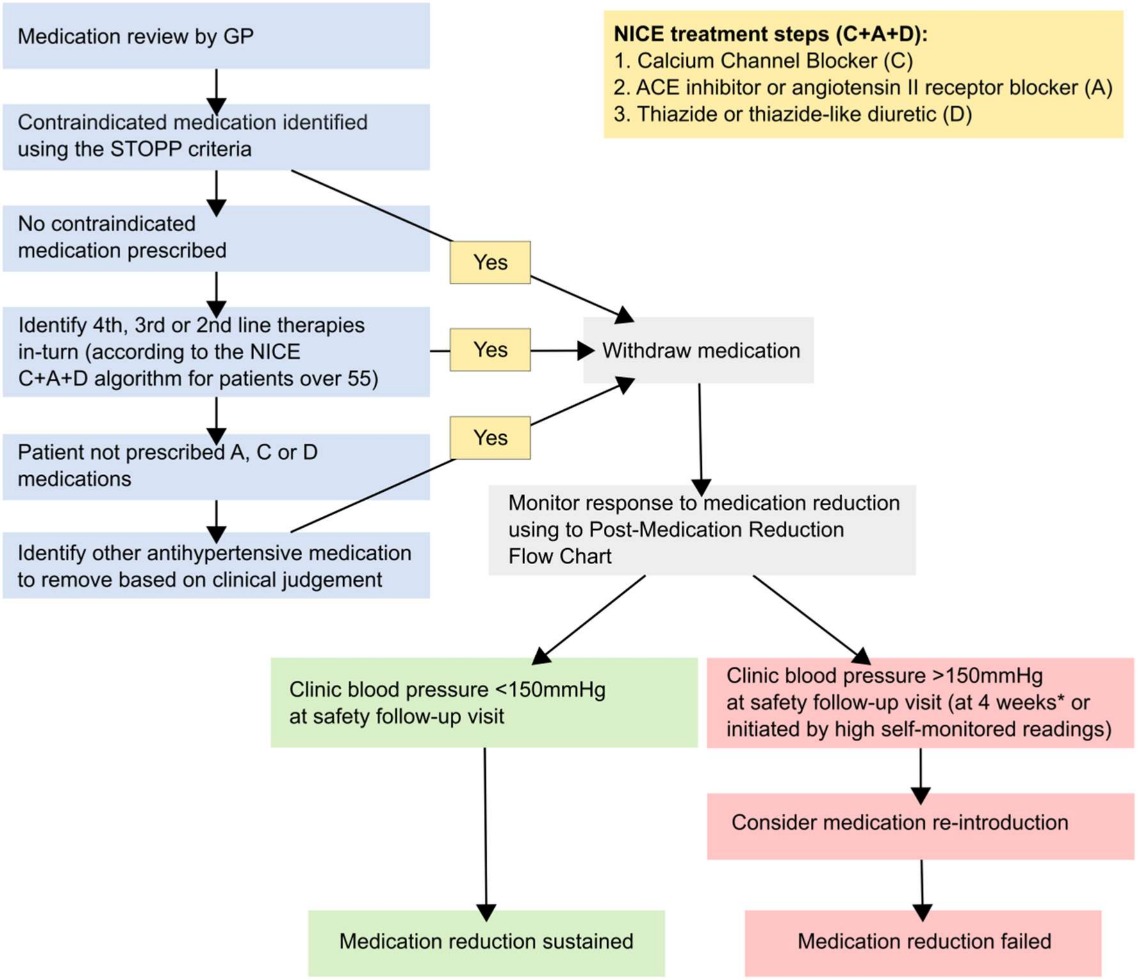

*Initial safety follow-up visit at 4 weeks may vary depending on side effects experienced and a repeat safety follow-up visit 1 week later may be appropriate before re-introducing medication – GPs/other appropriate, delegated healthcare professionals are asked to follow post medication reduction monitoring flow chart (Appendix E).

STOPP criteria[45]

Withdraw the one of the following medications if any of the ensuing contraindications are identified:

- Thiazide diuretic with a history of gout (may exacerbate gout).
- Beta-blocker in combination with verapamil (risk of symptomatic heart block).
- Non-cardioselective beta-blocker with chronic obstructive pulmonary disease (risk of bronchospasm).
- Calcium channel blockers with chronic constipation (may exacerbate constipation).
- Use of diltiazem or verapamil with NYHA Class III or IV heart failure (may worsen heart failure).

25. APPENDIX E: POST MEDICATION REDUCTION MONITORING FLOW CHART

- The full effects of most oral antihypertensives can last for up to 4-6 weeks. Frequent monitoring in the initial 4 weeks after drug withdrawal is thus not required unless BP levels are extreme or there are other clinical concerns (see below).
- Where systolic/diastolic BP values fall into different categories, consider the higher value
- BP should be taken as the averaged second and third measurements using a validated monitor
- Standard clinical care/monitoring should align with NICE recommendations[46]

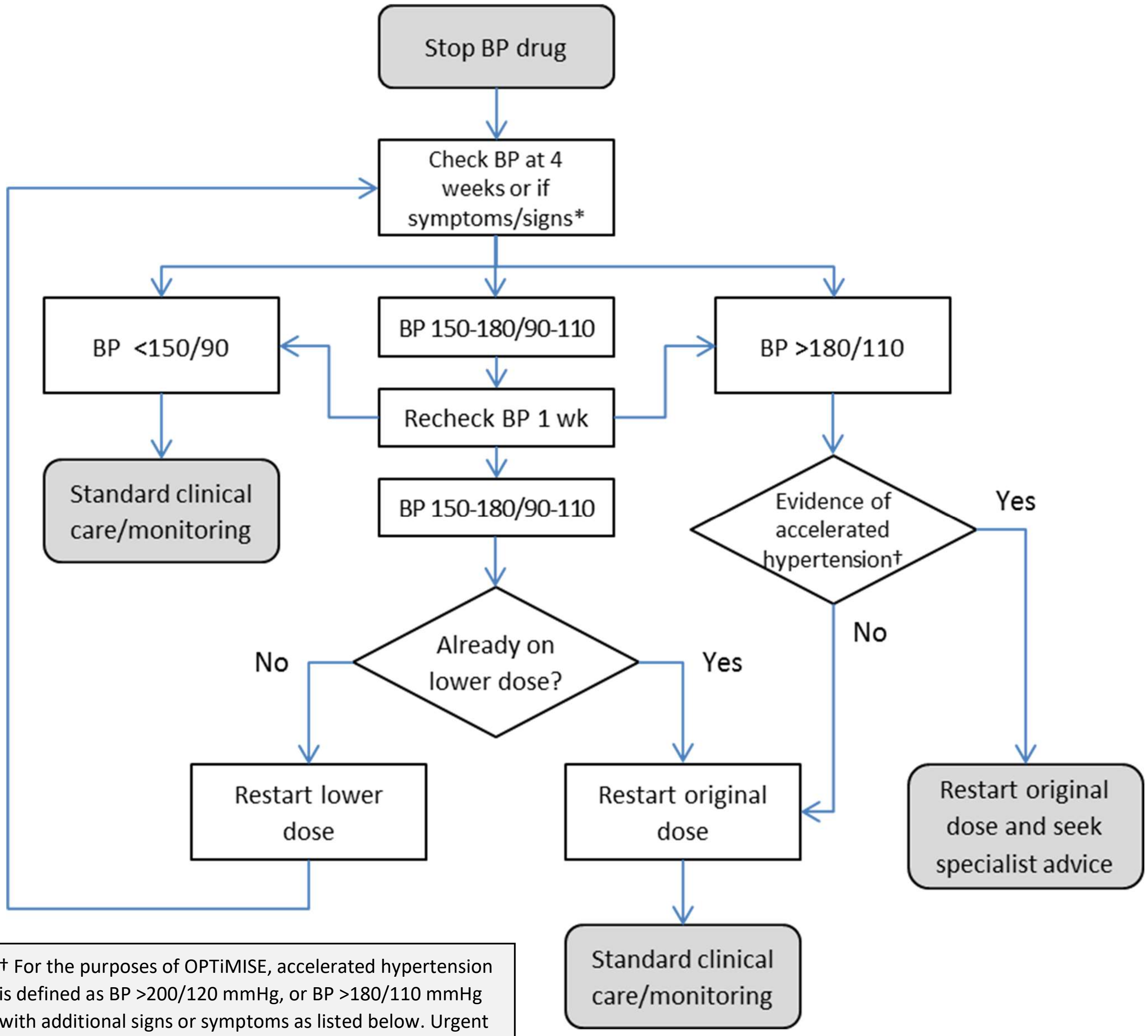

† For the purposes of OPTiMISE, accelerated hypertension is defined as BP >200/120 mmHg, or BP >180/110 mmHg with additional signs or symptoms as listed below. Urgent (same day) expert opinion should be sought.

- Neurological symptoms: headache, seizures, confusion, cerebrovascular event
- Respiratory symptoms: breathlessness, pulmonary oedema (and other signs of heart failure)
- Cardiac symptoms: Chest pain
- Vision problems: Visual disturbance, Papilloedema
- Other symptoms: Nausea and vomiting

*Signs and symptoms directly related to elevated BP are not anticipated, but BP should checked if any of the following symptoms occur:

- Palpitations (withdrawal of rate-limiting drug such as verapamil, diltiazem or beta-blocker)
- Prostatism (withdrawal of alpha blocker)
- Peripheral oedema (withdrawal of diuretic)

26. APPENDIX F: SELF-MONITORING PROTOCOL (TRAFFIC LIGHT SYSTEM)

UNDERSTANDING YOUR MEASUREMENTS

For patients 80 years and over

For RED or BLUE readings you will need to repeat them initially and if they remain too high or too low you will be advised to seek medical advice.

In each case, the top reading is the SYStolic and bottom reading DIAstolic.

Colour	Level	Blood Pressure	Action
RED	HIGH	SYS 171 or more<br>OR<br>DIA 106 or more	**Your BP is too high.**<br>Make an appointment within 48 hours to see your GP or nurse.
AMBER	RAISED	SYS 146-170<br>OR<br>DIA 86-105	**Your BP is raised.**<br>If you have persistent AMBER readings (4 or more days of the week) then you should contact from your GP/Practice nurse as you may need your medication altered.
GREEN	NORMAL	SYS 100-145<br>AND<br>DIA 85 or less	**Your BP is normal.**<br>This is fine provided that you have no side effects.
BLUE	LOW	SYS 99 or less	**Your BP is too low.**<br>Make an appointment within 48 hours to see your GP or nurse.

27. APPENDIX G: AMENDMENT HISTORY

Amendment No.	Protocol Version No.	Date issued	Author(s) of changes	Details of Changes made
1	2.0	13.01.2017	Sheppard, J.; McManus, R.; Temple, E.	The expectedness of SARs must be assessed more appropriately in the context of this trial. The expectedness of adverse events occurring as a result of re-introduction of withdrawn medication will be determined according to the latest version of the Summary of medicinal Product Characteristics (SmPC, section 4.8). There are no sections of the SmPC, or previous clinical studies which detail expected adverse events as a result of medication withdrawal (the study IMP) and therefore all SAEs at least possibly related, and not as a result of re-introduction of withdrawn medication, will be considered unexpected and reported as SUSARs. This replaces wording that SAEs will not be assessed for expectedness.<br><br>The definition of SUSAR was also clarified in Section 12.1 for the context of this trial.<br><br>Unclear definitions of adverse events were also removed to avoid confusion.<br><br>It was also clarified that adverse events that are observed by the Investigator or reported by the participant may be reported at any time.
2	3.0	15.11.17	Sheppard, J.; McManus, R.; Temple, E.	The eligibility criteria were clarified to include a more complete list of antihypertensives and to make it clear that no antihypertensive medications can have changed in the past 4 weeks for a patient to be eligible.<br><br>Amended text to ensure the guidelines for re-introducing

				antihypertensive medication are consistent throughout, referring to Appendix E.<br><br>Certain trial procedures written in the protocol were changed to allow the GP to delegate other appropriate people to do them, where appropriate (see protocol for details of appropriate delegates for each task).<br><br>P30 changed to read, 'All interviews that are transcribed will be transcribed verbatim'<br><br>P23 changed to read, 'Blood pressure will be measured using the clinically validated BpTRU blood pressure monitor'
4	4.0	07.09.18	Sheppard, J.; McManus, R.; Temple, E.	Amended text to clarify that patients who are already booked in for a consent visit once 540 participants have been randomised may be recruited.<br><br>A secondary objective of 'Determine the difference in the change in mean clinic diastolic blood pressure (from baseline) between the two groups at 12 week follow-up' was added.<br><br>A corresponding secondary outcome measure of 'Change in mean clinic diastolic blood pressure (from baseline) at 12 week follow-up' was added.<br><br>'Previous myocardial infarction' is not measured as part of the electronic Frailty Index, Appendix C has been corrected accordingly.<br><br>Planned trial period amended to 31/12/2024 to include the 5 year long term follow up already specified in the protocol.
5	4.1	29.10.2021	Sheppard, J.; McManus, R.; Smith, A	We have made changes to give further clarification to the methods of data collection and analysis for the

				long term follow-up element of the OPTiMISE trial. The long term outcome measures are further clarified in Appendix H.<br><br>We have included British Heart Foundation as funders for the long term follow-up.
6 (NS01)	6.0	17.04.2023	Sheppard, J.; McManus, R.; Smith, A	Changes to the long term follow-up section of the study Protocol (section 13.1.1) to 1.clarify that emergency hospitalisations refer to instances where this is as an in-patient, 2. to remove revascularisation within the definition of cardiovascular disease as although this is a type of CVD it is usually planned rather than sudden and resulting in emergency hospitalisation, 3. clarify that we will only be collecting primary care consultations relating to hypertension and not all primary care consultations, and 4. to update the details of the Sponsor's office.

Protocol amendments must be submitted to the Sponsor for approval prior to submission to the REC committee or MHRA.

28. APPENDIX H: LONG TERM FOLLOW-UP

We will collect the following data for all participants giving consent for long-term follow-up in the OPTiMISE trial.

	**Objectives**	**Outcome Measures**	**Timepoint**
**Primary**	Determine the difference in time to all-cause hospitalisations or death between medication reduction and usual care	All-cause hospitalisation or death during follow-up	≥3 years post randomisation (maximum follow-up possible)
**Secondary**	Determine the difference in time to emergency hospitalisation between medication reduction and usual care	Emergency hospitalisation (all-cause admissions which are unpredictable and at short notice because of clinical need; ‘method of admission’ codes 21-25 and 28 [admission where	≥3 years post randomisation (maximum follow-up possible)

		this is as an in-patient]) during follow-up	
	Determine the difference in time to all-cause death between medication reduction and usual care	All-cause death during follow-up	≥3 years post randomisation (maximum follow-up possible)
	Determine the difference in cardiovascular disease between medication reduction and usual care	Hospitalisation or death with cardiovascular disease (defined as:<br>i) myocardial infarction<br>ii) stroke<br>iii) heart failure)<br>during follow-up	≥3 years post randomisation (maximum follow-up possible)
	Determine the difference in stroke between medication reduction and usual care	Hospitalisation or death with stroke during follow-up	≥3 years post randomisation (maximum follow-up possible)
	Determine the difference in myocardial infarction between medication reduction and usual care	Hospitalisation or death with myocardial infarction event during follow-up	≥3 years post randomisation (maximum follow-up possible)
	Determine the difference in hospitalisations due to falls between medication reduction and usual care	Hospitalisation due to falls during follow-up	≥3 years post randomisation (maximum follow-up possible)
	Determine the difference in hospitalisations with acute kidney injury between medication reduction and usual care	Hospitalisation with acute kidney injury during follow-up	≥3 years post randomisation (maximum follow-up possible)
	Determine the difference in hospitalisations with syncope between medication reduction and usual care	Hospitalisation with syncope during follow-up	≥3 years post randomisation (maximum follow-up possible)
	Determine the difference in hospitalisations with hypotension between medication reduction and usual care	Hospitalisation with hypotension during follow-up	≥3 years post randomisation (maximum follow-up possible)
	Determine the difference in	Hospitalisation with fracture during follow-up	≥3 years post randomisation

	hospitalisations with fracture between medication reduction and usual care		(maximum follow-up possible)
	Determine the difference in hospitalisations with electrolyte abnormalities between medication reduction and usual care	Hospitalisation with electrolyte abnormalities during follow-up	≥3 years post randomisation (maximum follow-up possible)
	Determine the difference in diagnoses of dementia between medication reduction and usual care	Diagnosis of dementia during follow-up	≥3 years post randomisation (maximum follow-up possible)
	Determine the difference in the change of antihypertensive medication prescription (from baseline) between medication reduction and usual care	Number of antihypertensive medication prescription (from randomisation) at 3 year follow-up.	3 years post randomisation
	Determine the proportion of patients in intervention arm who maintain medication reduction throughout follow-up	Proportion of patients randomised to the intervention arm who maintain medication reduction	3 years post randomisation
	Determine the difference in all prescribed medications between medication reduction and usual care	Number of prescribed medications at 3 year follow-up	3 years post randomisation
	Determine the difference in the change in mean clinic systolic blood pressure (from baseline) between medication reduction and usual care	Change in mean clinic systolic blood pressure (from randomisation) at 3 year follow-up.	Date of the routine blood pressure reading taken closest to 3 years post randomisation
	Determine the difference in the change in mean clinic diastolic blood pressure (from baseline) between medication reduction and usual care	Change in mean clinic diastolic blood pressure (from randomisation) at 3 year follow-up	Date of the routine blood pressure reading taken closest to 3 years post randomisation

	Determine the difference in the proportion of patients with clinically safe levels (defined as the proportion of patients with SBP <150mmHg) between medication reduction and usual care	The proportion of patients with controlled systolic blood pressure at 3 year follow-up	Date of the routine blood pressure reading taken closest to 3 years post randomisation
	Determine the difference in primary care consultations relating to hypertension between medication reduction and usual care	Number of primary care consultations relating to hypertension (reported by staff type undertaking the consultation) during 3 year follow-up	3 years post randomisation
**Exploratory analyses**	Subgroup analyses by different levels of baseline frailty (electronic frailty index score ±0·12 [fit vs. frail])	• All-cause hospitalisation or death<br>• Change in mean clinic systolic blood pressure (from randomisation)<br>• Proportion of patients with controlled systolic blood pressure	≥3 years post randomisation (maximum follow-up possible)
	Subgroup analyses by different levels of baseline functional independence (Modified Rankin score ±2)	• All-cause hospitalisation or death<br>• Change in mean clinic systolic blood pressure (from randomisation)<br>• Proportion of patients with controlled systolic blood pressure	≥3 years post randomisation (maximum follow-up possible)
	Subgroup analyses by different levels of baseline cognitive function (MoCA score ±26)	• All-cause hospitalisation or death<br>• Change in mean clinic systolic blood pressure (from randomisation)<br>• Proportion of patients with controlled systolic blood pressure	≥3 years post randomisation (maximum follow-up possible)
	Subgroup analyses by number of antihypertensive medications prescribed at baseline (±3 medications)	• All-cause hospitalisation or death<br>• Change in mean clinic systolic blood pressure (from randomisation)<br>• Proportion of patients with controlled systolic blood pressure	≥3 years post randomisation (maximum follow-up possible)

	Subgroup analyses by number of co-morbidities at baseline (±4 morbidities)	• All-cause hospitalisation or death<br>• Change in mean clinic systolic blood pressure (from randomisation)<br>• Proportion of patients with controlled systolic blood pressure	≥3 years post randomisation (maximum follow-up possible)

Appendix 3. OPTiMISE Trial Extension Statistical Analysis Plan V1.0 27.09.2023

Primary Care Clinical Trials Unit

STATISTICAL ANALYSIS PLAN

OPTiMISE: Optimising Treatment for Mild Systolic Hypertension in the Elderly

OPTiMISE: OPtimising Treatment for MIld Systolic hypertension in the Elderly: a randomised controlled trial *(N.B. This covers the 3 year long term follow-up only, other analyses are covered in a separate Statistical Analysis Plan)*

Version number and date: 1.0 27.09.2023

Based on protocol version 6.0 dated 17.04.2023

Table of Contents

Table of Contents 68

1 Introduction 70

1.1 Preface 70

1.2 Purpose and scope of the plan 70

1.3 Trial overview 71

1.4 Objectives 71

2 Trial design 77

2.1 Outcomes measures 77

2.1.1 Primary outcome 78

2.1.2 Secondary outcomes 78

2.1.3 Non-protocol-specified outcomes 89

2.2 Target population 104

2.3 Sample size 105

2.4 Randomisation and blinding in the analysis stage 105

3 Analysis – General considerations 106

3.1 Descriptive statistics 106

3.2 Characteristics of participants 106

3.3 Definition of population for analysis 106

3.4 Pooling of investigational sites 106

3.5 Data Monitoring Committee And Interim Analyses 106

4 PRIMARY ANALYSIS 107

4.1 Primary outcome 107

4.2 Handling missing data 107

4.3 Handling outliers 107

4.4 Handling multi-centre/clustered data 107

4.5 Multiple comparisons and multiplicity 107

4.6 Model assumptions 108

5 SECONDARY ANALYSIS 108

5.1 Per-protocol analysis of primary outcome 108

5.2 Time-to-event outcomes 108

5.3 Count outcomes 109

5.4 Binary outcomes 109

5.5 Blood pressure outcomes 109
5.6 Maintenance of medication reduction 110
5.7 Count of primary care consultations 110
5.8 Change of antihypertensive medication prescription 111
5.9 Difference in all prescribed medications 111
5.10 Increase/decrease/maintenance of antihypertensive medication (non-protocol-specified outcome) 111
6 SENSITIVITY ANALYSIS 111
7 SUBGROUP ANALYSES 112
8 VALIDATION 113
9 CHANGES TO THE PROTOCOL OR PREVIOUS VERSIONS OF SAP 113
10 References 114
11 Appendices 116
11.1 Appendix I. Flow chart of study activities up to 12 weeks follow-up 116
11.2 Appendix II. Outcome assessment schedule 117
11.3 Appendix III. Flow diagram of trial participants 118
11.4 Appendix IV. Number of antihypertensive drugs in medications: 119
11.5 Appendix V. List of ICD-10 codes to identify outcomes from NHS England data 122

1 Introduction

Trial title: OPtimising Treatment for MIld Systolic hypertension in the Elderly: a randomised controlled trial

Short title: OPTiMISE

Ethics Ref: 16/SC/0628

This Statistical Analysis Plan (SAP) supports the long-term follow up (at least 3 years post-randomisation) aspects of version 6.0 of the protocol, dated 17th April 2023

1.1 Preface

Chief Investigator: Professor Richard McManus – richard.mcmanus@phc.ox.ac.uk

Lead Scientific Investigator: Dr James Sheppard – james.sheppard@phc.ox.ac.uk

Clinical Trial Manager: Anne Smith – anne.smith@phc.ox.ac.uk

Trial Statistician: Eleanor Temple – eleanor.temple@phc.ox.ac.uk

Lead Statistician: Professor Ly-Mee Yu – ly-mee.yu@phc.ox.ac.uk

Data Manager: David Watt – david.watt@phc.ox.ac.uk

1.2 Purpose and scope of the plan

This plan covers the proposed analysis to be carried out for the long term follow-up objectives and outcomes listed in Appendix H of the OPTiMISE protocol. Analysis for the main OPTiMISE trial, covering analysis of whether antihypertensive medication reduction can achieve clinically safe levels of blood pressure at 12 weeks from randomisation, has been detailed in a separate OPTiMISE SAP previously and will not be covered here. This SAP covers only the long term follow-up aspects of the trial to assess whether antihypertensive medication reduction in elderly participants leads to increases/decreases in being hospitalised or dying over 3 years following recruitment into the trial.

Results reported in any papers reporting these long term results should follow this strategy. Subsequent analyses of a more exploratory nature will not be bound by this strategy, though they are expected to follow the broad principles laid down here. The principals are not intended to curtail exploratory analysis (for example, to decide cut-points for categorisation of continuous variables), nor to prohibit accepted practices (for example, data transformation prior to analysis), but they are intended to establish the rules that will be followed, as closely as possible, when analysing and reporting the trial.

The analysis strategy will be available on request when the principal papers are submitted for publication in a journal. Suggestions for subsequent analyses by journal editors or referees will be considered carefully, and carried out as far as possible in line with the principles of this analysis strategy; if reported, the source of the suggestion will be acknowledged.

Any deviations from this statistical analysis plan will be described and justified in the final report of this long term follow-up part of the trial. The analysis should be carried out by an identified, appropriately qualified, and experienced statistician, who should ensure the integrity of the data

during their processing. Examples of such procedures include quality control and evaluation procedures.

1.3 Trial overview

The population is ageing[56] and, consequently, the number of people living with age-related chronic conditions is increasing[57]. Polypharmacy is common in older persons, with up to 20% of those aged >80 years prescribed ten or more medications[58]. Polypharmacy is associated with increased risk of adverse drug reactions and frequent inappropriate prescribing [59,60]. Indeed, as many as 29% of elderly people are thought to receive potentially inappropriate prescriptions in Primary Care[61].

Hypertension is the number one co-morbid condition in older people with multiple chronic conditions [62]and 52% of those aged >80 years are prescribed two or more antihypertensive medications (equivalent to approximately 1.25 million people in the UK)[63]. Blood pressure lowering has been shown to be effective at preventing stroke and cardiovascular disease in healthy individuals aged >80 years with stage 2 hypertension (systolic blood pressure of >160mmHg)[64]. However, more recent evidence suggests that larger blood pressure reductions and multiple antihypertensive prescriptions may be harmful in older people[65,66]. A meta-analysis by Bejan-Angoulvant et al., found that large reductions in systolic blood pressure and higher intensity treatment may be associated with increased risk of all-cause mortality[65]. Evidence from observational studies also suggests that higher intensity blood pressure treatment is associated with increased risk of falls in older people[67], although this is also disputed[64].

Some patients consider the increased risk of falls and other adverse events to be as important as the risk of MI or stroke, particularly those taking medications for primary prevention of cardiovascular disease[68]. Thus, decisions over blood pressure lowering in the elderly, particularly the frail elderly, require the weighing of harms and quality of life. Studies of patients' attitudes towards hypertension treatment suggest there is widespread dislike of treatment and its side effects, fear of the long-term impact of taking medication, and consequent intentional non-adherence to treatment[69]. However, clinicians can often struggle to stop prescribing medication due to a perceived lack of evidence, fear of the reaction of other prescribers, and concern that patients will feel their care is being cut[70,71].

The 12 weeks following participant randomisation, requiring participant involvement aimed to answer whether antihypertensive medication reduction could be done safely, maintaining controlled blood pressure after 12 weeks. The results of this immediate follow-up of participants have been published[72]. The aim of the long term follow-up, looking is to assess whether antihypertensive medication reduction affects numbers of hospital stays or deaths three years after randomisation.

1.4 Objectives

The following objectives are for the long term follow-up aspects of the OPTiMISE trial that this SAP describes analysis for.

	**Objectives**	**Outcome Measures**	**Timepoint**
**Primary**	Determine the difference in time to all-cause hospitalisations or death between	Time to all-cause hospitalisation or death since randomisation	At least 3 years post randomisation (maximum follow-up possible)

	medication reduction and usual care		
**Secondary**	Determine the difference in time to emergency hospitalisation between medication reduction and usual care	Time to and count of emergency hospitalisations (all-cause admissions which are unpredictable and at short notice because of clinical need; 'method of admission' codes 21-25 and 28 [admission where this is as an in-patient]) from randomisation	At least 3 years post randomisation (maximum follow-up possible)
	Determine the difference in time to all-cause death between medication reduction and usual care	Participant died or not and time to all-cause death during follow-up	At least 3 years post randomisation (maximum follow-up possible)
	Determine the difference in cardiovascular disease between medication reduction and usual care	Time to and count of hospitalisation or death due to cardiovascular disease (defined as: i) myocardial infarction ii) stroke iii) heart failure) during follow-up	At least 3 years post randomisation (maximum follow-up possible)
	Determine the difference in stroke between medication reduction and usual care	Time to and count of hospitalisation or death due to stroke during followup	At least 3 years post randomisation (maximum follow-up possible)
	Determine the difference in myocardial infarction between medication reduction and usual care	Time to and count of hospitalisation or death due to myocardial infarction event during follow-up	At least 3 years post randomisation (maximum follow-up possible)
	Determine the difference in hospitalisations due to falls between medication reduction and usual care	Time to and count of hospitalisation due to falls during follow-up	At least 3 years post randomisation (maximum follow-up possible)
	Determine the difference in hospitalisations with acute kidney injury	Time to and count of hospitalisation due to acute kidney injury during followup	At least 3 years post randomisation (maximum follow-up possible)

	between medication reduction and usual care		
	Determine the difference in hospitalisations with syncope between medication reduction and usual care	Time to and count of hospitalisation due to syncope during follow-up	At least 3 years post randomisation (maximum follow-up possible)
	Determine the difference in hospitalisations with hypotension between medication reduction and usual care	Time to and count of hospitalisation due to hypotension during followup	At least 3 years post randomisation (maximum follow-up possible)
	Determine the difference in hospitalisations with fracture between medication reduction and usual care	Time to and count of hospitalisation due to fracture during follow-up	At least 3 years post randomisation (maximum follow-up possible)
	Determine the difference in hospitalisations with electrolyte abnormalities between medication reduction and usual care	Time to and count of hospitalisation due to electrolyte abnormalities during follow-up	At least 3 years post randomisation (maximum follow-up possible)
	Determine the difference in diagnoses of dementia between medication reduction and usual care	Participant received dementia diagnosis or not and time to diagnosis of dementia during follow-up	At least 3 years post randomisation (maximum follow-up possible)
	Determine the difference in the change of antihypertensive medication prescription (from baseline) between medication reduction and usual care	Change in number of antihypertensive medications prescribed (from randomisation) for each participant at 3 year follow-up.	3 years post randomisation
	Determine the proportion of patients in intervention arm who maintain medication reduction throughout follow-up	Participant maintained medication reduction or not (intervention arm only)	3 years post randomisation

	Determine the difference in all prescribed medications between medication reduction and usual care	Number of prescribed medications for each participant at 3 year follow-up	3 years post randomisation
	Determine the difference in the change in mean clinic systolic blood pressure (from baseline) between medication reduction and usual care	Change in mean clinic systolic blood pressure (from randomisation) at 3 year follow-up.	Date of the routine blood pressure reading taken closest to 3 years post randomisation
	Determine the difference in the change in mean clinic diastolic blood pressure (from baseline) between medication reduction and usual care	Change in mean clinic diastolic blood pressure (from randomisation) at 3 year follow-up	Date of the routine blood pressure reading taken closest to 3 years post randomisation
	Determine the difference in the proportion of patients with clinically safe levels (defined as the proportion of patients with SBP <150mmHg) between medication reduction and usual care	Participant has controlled systolic blood pressure or not at 3 year follow-up	Date of the routine blood pressure reading taken closest to 3 years post randomisation
	Determine the difference in primary care consultations relating to hypertension between medication reduction and usual care	Count of primary care consultations relating to hypertension (reported by staff type undertaking the consultation) during 3 year follow-up	3 years post randomisation
**Exploratory analyses**	Subgroup analyses by different levels of baseline frailty (electronic frailty index score ≤0·12 vs >0.12 [fit vs. frail])	• Time to all-cause hospitalisation or death • Change in mean clinic systolic blood pressure (from randomisation) • Proportion of patients with controlled systolic blood pressure	• At least 3 years post randomisation (maximum follow-up possible) for hospitalisation or death. • Date of the routine blood pressure reading taken closest to 3 years post randomisation for

			blood pressure outcome measures.
	Subgroup analyses by different levels of baseline functional independence (Modified Rankin score ≤2 vs >2)	• All-cause hospitalisation or death • Change in mean clinic systolic blood pressure (from randomisation) • Proportion of patients with controlled systolic blood pressure	• At least 3 years post randomisation (maximum follow-up possible) for hospitalisation or death.<br>• Date of the routine blood pressure reading taken closest to 3 years post randomisation for blood pressure outcome measures.
	Subgroup analyses by different levels of baseline cognitive function (MoCA score <26 vs ≥26)	• All-cause hospitalisation or death • Change in mean clinic systolic blood pressure (from randomisation) • Proportion of patients with controlled systolic blood pressure	• At least 3 years post randomisation (maximum follow-up possible) for hospitalisation or death.<br>• Date of the routine blood pressure reading taken closest to 3 years post randomisation for blood pressure outcome measures.
	Subgroup analyses by number of antihypertensive medications prescribed at baseline (2 vs ≥3 medications)	• All-cause hospitalisation or death • Change in mean clinic systolic blood pressure (from randomisation) • Proportion of patients with controlled systolic blood pressure	• At least 3 years post randomisation (maximum follow-up possible) for hospitalisation or death.<br>• Date of the routine blood pressure reading taken closest to 3 years post randomisation for blood pressure outcome measures.
	Subgroup analyses by number of comorbidities at baseline (≤4 vs >4 morbidities)	• All-cause hospitalisation or death • Change in mean clinic systolic blood pressure (from randomisation) • Proportion of patients with controlled systolic blood pressure	• At least 3 years post randomisation (maximum follow-up possible) for hospitalisation or death.<br>• Date of the routine blood pressure reading taken closest to 3 years post

			randomisation for blood pressure outcome measures.

2 Trial design

This trial used a Primary Care based, open label, randomised controlled trial design. 540 elderly participants (aged at least 80 years old) with controlled blood pressure who were taking 2 or more antihypertensive medications were required to be randomised to be able to answer the research question. Potential participants were invited to attend a screening visit at their GP practice and those fulfilling the eligibility criteria and who gave informed consent underwent baseline measurements for the study. Extracted data was entered directly into the study database using eCRFs. Following baseline measurements, individuals were randomised to a strategy of medication reduction (intervention) or usual care (control). Those in the intervention arm were invited to self-monitor their blood pressure, reporting any consistently high readings to their GP/other appropriate, delegated healthcare professional. All individuals in the intervention arm of the trial were asked to attend a routine safety follow-up visit with their GP/other appropriate, delegated healthcare professional, four weeks (±2 weeks) after randomisation. All patients were asked to attend a 12 week (±2 weeks) follow-up with the trial facilitator, either at their GP practice or at their home; the trial facilitator repeated all measurements taken at baseline. The trial up to 12 weeks of initial follow-up is shown in the flow diagram in section 32.1. After 12 week follow-up, there were no further face-to-face visits, but long-term follow-up of mortality, hospital admissions and primary care data at least 3 years post-randomisation will be undertaken via NHS England's patient tracking service and medical notes review. This analysis details how this remote long-term follow-up of data will be used.

2.1 Outcomes measures

A summary of the study objectives and outcomes can be found in section 1.4. All of the timepoints to measure these long term follow-up outcomes, relevant to this SAP are also listed in the table in section 1.4. There are three intended sources of data for the long-term follow-up: NHS England data, ORCHID data and manual notes review. Some data will be captured by more than one data source for each participant. There will be no data checking/querying between sources of data. The hierarchy of priority of data sources, where there is more than one source for a particular variable is: NHS England data; then ORCHID data; then manual notes review data. This priority order reflects the less room for human error from the NHS England and ORCHID datasets. However, where it was not possible to link a participant to NHS datasets held by NHS England, the datasets from the other sources will be used for that participant. All diagnosis codes have been requested from NHS England so where diagnoses form part of the outcome, an event will be determined as having occurred if a diagnosis code is present, regardless of whether it is a primary or secondary diagnosis. For time to first event outcomes, if the event did not take place before the date of 3 year follow-up, the date of censoring will be taken as:

1. Date of death (if it is not the outcome of interest and is not the same date as the outcome of interest).
2. If data from NHS England is being used in this analysis: last date of any admission found in the admissions NHSE dataset,
3. If data from ORCHID is being used in this analysis: date of ORCHID data extraction,
4. If data from manual notes review is being used in this analysis: date participant noted to have de-registered from the practice or date noted that electronic medical notes became

inactive in manual notes review (notes made in *alt_id_mnr* variable) or if no date recorded here, date of follow-up for manual notes review (*fu_dat_mnr)*.

Date of death will be taken from either NHSE data or from the manual notes review data, if either is available. If both are available and the dates of death differ, the date of death provided by NHSE will be used.

2.1.1Primary outcome

The primary outcome is a composite variable of time to first event of all-cause hospitalisation and death during follow-up. The date of first hospitalisation for any reason or date of death (whichever comes first) after randomisation from data received from NHS England, will be used to calculate time to first hospitalisation/death. If it is not possible to link data from NHS England to a participant (for example, if the participant withdrew from data collection in the trial or it has not been possible to obtain a participant's NHS number), the manual notes review will be used for that participant. From the manual notes review, the date of first hospitalisation *(inpadat[1-25]_mnr)* or the date of death *(dthdt_mnr)* if the participant has died (*dthyn_mnr = yes)* (whichever comes first during the follow-up) will be used. At least 3 years post-randomisation, details of all hospitalisations between randomisation and the time of data collection are collected. These details include date of admission. Whether or not the participant has died since randomisation and date of death, where applicable, are also collected at least 3 years post-randomisation. The data will be collected from randomisation and date of first hospitalisation or death could be any time between randomisation and time of data collection (at least 3 years post-randomisation).

In the instance that participant has died and date of death is not known the date of death will be estimated as the latest date of any data collected about the participant from NHS England, or (where NHS England data is not available for the participant), from the latest date of the data captured on the manual notes review as this is the last date of person being known to be alive.

The primary endpoint is the time to first hospitalisation or death, whichever comes first, from randomisation.

N.B. Where baseline CRF variables are referred to throughout, these may be from the baseline or rescreening visit CRFs, whichever is later. If a variable is present in both the baseline and rescreening CRFs then the latest rescreening CRF values will be used instead of baseline CRF values. If rescreening CRF the suffix will be *'_rs'* instead of *'_bl'*.

2.1.2 Secondary outcomes

3 Emergency hospitalisation

One of the secondary objectives is to determine the difference in time to emergency hospitalisation between medication reduction and usual care. Counts of emergency hospitalisation will also be measured:

i) **Time to first emergency hospitalisation** (where this is as an in-patient) will be calculated using the date of first emergency hospitalisation where the participant was admitted, after randomisation from data received from NHS England after randomisation. If it is not possible to link data from NHS England to a participant, the manual notes review will be used for that participant. In the manual notes review, dates of admissions between randomisation and time of follow-up are collected at time of follow-up (at least 3 years post-randomisation). The earliest date of admission *(inpadat[1-25]_mnr)* following

randomisation, from method of admissions selected as 'Emergency Admission' of any subtype *(inpcode_mnr=1-6)* will be used to calculate time from randomisation. If subtype of admission is unknown (i.e. *inpcode_mnr=8)* this admission will not be classed as emergency admission.

ii) **The count of all emergency hospitalisations** as defined in i) above by 3 years post-randomisation for each participant will be calculated. Any recorded beyond 3 years post-randomisation will not count towards this total. If event count is low, a binary indicator variable will be derived *(participant has emergency hospitalisation by 3 years post-randomisation (as defined in i) above) = 1, participant does not have emergency hospitalisation by 3 years post-randomisation (as defined in i) above) = 0)*. Where there is only an emergency hospitalisation recorded beyond 3 years post-randomisation this will be classed as not having had emergency hospitalisation by 3 years post-randomisation.

4 All-cause death

One of the secondary objectives is to determine the difference in time to all-cause death between medication reduction and usual care. Time to all-cause death as well as death as a binary outcome will be measured:

i) **Time to all-cause death** will be calculated using the date of all-cause death after randomisation from data received from NHS England.. If it is not possible to link data from NHS England to a participant, the manual notes review will be used for that participant. From the manual notes review for those participants who have died *(dthyn_mnr=yes)*, their date of death *(dthdt_mnr)* will be used to calculate the time from randomisation.

ii) **All-cause death at 3 year (Yes/No)** will be derived using the date of all-cause death after randomisation from data received from NHS England*(participant died at 3 years post-randomisation = 1, alive at 3 years post-randomisation = 0)*. If it is not possible to link data from NHS England to a participant, the manual notes review will be used for that participant. Any death after 3 years post-randomisation will be classed as alive at 3 years post-randomisation:

:

- Participant died at 3 years post-randomisation =1 if participant has died *(dthyn_mnr=yes)* and date of death *(dthdt_mnr)* is ≤3 years post-randomisation
- Participant died at 3 years post-randomisation =0 if:
  - participant has not died (*dthyn_mnr=no)*

    OR
  - Participant has died (*dthyn_mnr=yes)* AND *date of death (dthdt_mnr) >3 years post-randomisation*

In the instance that participant has died and date of death is not known, the date of death will be estimated as the latest date of any data collected about the participant from NHS England, or (where NHS England data is not available for the participant), from the latest date of the data captured on the manual notes review as this is the last date of person being known to be alive.

5 Cardiovascular disease

One of the secondary objectives is to determine the difference in cardiovascular disease between medication reduction and usual care. Time to first hospitalisation or death due to cardiovascular disease as well as count of hospitalisations and death due to cardiovascular disease will be measured:

i) **Time to first hospitalisation or death due to cardiovascular disease** after randomisation will be measured using the date of first record of cardiovascular disease or death due to cardiovascular disease (see section 32.5 for corresponding ICD-10 codes) after randomisation from data received from NHS England at least 3 years post-randomisation. If it is not possible to link data from NHS England to a participant, the manual notes review will be used for that participant. From the manual notes review the date of relevant admission or date of death (whichever is earliest) will be used. Date of hospitalisations with cardiovascular disease is defined as date of admissions *(inpadat[1-25]_mnr)* following randomisation, where the reason for hospitalisation is given as either: myocardial infarction; stroke heart failure *(inpreas[1-25]_mnr=1-4)*. Date of death with cardiovascular disease is defined as date of death *(dthdt_mnr)* when participant has died *(dthyn_mnr =yes)*, where cause of death is given as any of: myocardial infarction; stroke; heart failure *(dthcause_mnr=1-4).*

ii) **The count of all hospitalisations or death due to cardiovascular disease** as defined in i) above, which are ≤3 years post-randomisation for each participant will be calculated. Any recorded beyond 3 years post-randomisation will not count towards this total. If event count is low, a binary indicator variable will be derived *(participant has hospitalisation or death due to cardiovascular disease by 3 years post-randomisation (as defined in i) above) = 1, participant does not have hospitalisation or death due to cardiovascular disease by 3 years post-randomisation (as defined in i) above) = 0)*. Where there is only a hospitalisation or death due to cardiovascular disease recorded beyond 3 years post-randomisation this will be classed as not having had hospitalisation or death due to cardiovascular disease by 3 years post-randomisation.

6 Stroke

One of the secondary objectives is to determine the difference in stroke between medication reduction and usual care. Time to first hospitalisation or death due to stroke as well as count of hospitalisations and death due to strokes will be measured:

i) **Time to first hospitalisation or death due to stroke after randomisation** will be measured using the date of first record of stroke or death due to stroke (see section 32.5 for corresponding ICD-10 codes) after randomisation from data received from NHS England at least 3 years post-randomisation. If it is not possible to link data from NHS England to a participant, the manual notes review will be used for that participant. From the manual notes review the dates of relevant admissions or date of death (whichever is earliest) will be used. Date of a hospitalisation due to stroke is defined as date of admission *(inpadat[1-25]_mnr)* following randomisation, where the reason for hospitalisation is given as stroke *(inpreas_mnr=2)*. Date of death due to stroke is defined as date of death *(dthdt_mnr)* when participant has died *(dthyn_mnr=yes)*, where cause of death is given as stroke *(dthcause_mnr=2).*

ii) **The count of all hospitalisations or death due to stroke** as defined in i) above by 3 years post-randomisation for each participant will be calculated. Any recorded beyond 3 years post-randomisation will not count towards this total. If event count is low, a binary indicator variable will be derived *(participant has hospitalisation or death due to stroke by 3 years post-randomisation (as defined in i) above) = 1, participant does not have hospitalisation or death due to stroke by 3 years post-randomisation (as defined in i) above) = 0)*. Where there is only a hospitalisation or death due to stroke recorded beyond 3 years post-randomisation this will be classed as not having had hospitalisation or death due to stroke by 3 years post-randomisation.

7 Myocardial infarction

One of the secondary outcomes is to determine the difference in myocardial infarction between medication reduction and usual care. Time to first hospitalisation or death due to myocardial infarction as well as count of hospitalisations and death due to myocardial infarction will be measured:

i) **Time to first hospitalisation or death due to myocardial infarction** after randomisation will be measured using the date of first record of myocardial infarction or death due to myocardial infarction (see section 32.5 for corresponding ICD-10 codes) after randomisation from data received from NHS England at least 3 years post-randomisation. If it is not possible to link data from NHS England to a participant, the manual notes review will be used for that participant. From the manual notes review the dates of relevant admissions or date of death (whichever is earliest) will be used. Date of a hospitalisation due to myocardial infarction is defined as date of admission *(inpadat[1-25]_mnr)* following randomisation, where the reason for hospitalisation is given as myocardial infarction *(inpreas_mnr=1)*. Date of death with myocardial infarction is defined as date of death *(dthdt_mnr)*
when participant has died *(dthyn_mnr=yes)*, where cause of death is given as myocardial infarction *(dthcause_mnr=1)*.

ii) **The count of all hospitalisations or death due to myocardial infarction** as defined in i) above which are ≤3 years post-randomisation for each participant will be calculated. Any recorded beyond 3 years post-randomisation will not count towards this total. If event count is low, a binary indicator variable will be derived *(participant has hospitalisation or death due to myocardial infarction by 3 years post-randomisation (as defined in i) above) = 1, participant does not have hospitalisation or death due to myocardial infarction by 3 years post-randomisation (as defined in i) above) = 0)*. Where there is only a hospitalisation or death due to myocardial infarction recorded beyond 3 years post-randomisation this will be classed as not having had hospitalisation or death due to myocardial infarction by 3 years post-randomisation.

8 Hospitalisation due to falls

One of the secondary outcomes is to determine the difference in hospitalisations due to falls between medication reduction and usual care. Time to first hospitalisation due to fall as well as count of hospitalisations due to fall will be measured:

i) **Time to first hospitalisation due to fall** after randomisation will be measured using the date of first record of hospitalisation due to fall (see section 32.5 for corresponding ICD-10 codes) after randomisation from data received from NHS England at least 3 years post-randomisation. If it is not possible to link data from NHS England to a participant, the manual notes review will be used for that participant. From the manual notes review the date of earliest relevant admission will be used. Date of a hospitalisation due to a fall is defined as date of admission *(inpadat[1-25]_mnr)* following randomisation, where the reason for hospitalisation is given as Falls *(inpreas_mnr=5)*.

ii) **The count of all hospitalisations due to falls** as defined in i) above by 3 years post-randomisation for each participant will be calculated. Any recorded beyond 3 years post-randomisation will not count towards this total. If event count is low, a binary indicator variable will be derived *(participant has hospitalisation due to fall by 3 years post-randomisation (as defined in i) above) = 1, participant does not have hospitalisation due to fall by 3 years post-randomisation (as defined in i) above) = 0)*. Where there is only a hospitalisation due to fall recorded beyond 3 years post-randomisation this will be classed as not having had hospitalisation due to fall by 3 years post-randomisation.

9 Hospitalisation due to acute kidney injury

One of the secondary outcomes is to determine the difference in hospitalisations due to acute kidney injury between medication reduction and usual care. Time to first hospitalisation due to acute kidney injury as well as count of hospitalisations due to acute kidney injury will be measured:

i) **Time to first hospitalisation due to acute kidney injury** after randomisation will be measured using the date of first record of hospitalisation due to acute kidney injury (see section 32.5 for corresponding ICD-10 codes) after randomisation from data received from NHS England at least 3 years post-randomisation. If it is not possible to link data from NHS England to a participant, the manual notes review will be used for that participant. From the manual notes review the date of earliest relevant admission will be used. Date of a hospitalisation due to acute kidney injury is defined as date of admission *(inpadat[1-25]_mnr)* following randomisation, where the reason for hospitalisation is given as acute kidney failure *(inpreas_mnr=6)*.

ii) **The count of all hospitalisations due to acute kidney injury** as defined in i) above by 3 years post-randomisation for each participant will be calculated. Any recorded beyond 3 years post-randomisation will not count towards this total. If event count is low, a binary indicator variable will be derived *(participant has hospitalisation due to acute kidney injury by 3 years post-randomisation (as defined in i) above) = 1, participant does not have hospitalisation due to acute kidney injury by 3 years post-randomisation (as defined in i) above) = 0)*. Where there is only a hospitalisation due to acute kidney injury recorded beyond 3 years post-randomisation this will be classed as not having had hospitalisation due to acute kidney injury by 3 years post-randomisation.

10 Hospitalisation due to syncope

One of the secondary outcomes is to determine the difference in hospitalisations due to syncope between medication reduction and usual care. Time to first hospitalisation due to syncope as well as count of hospitalisations due to syncope will be measured:

i) **Time to first hospitalisation due to syncope** after randomisation will be measured using the date of first record of hospitalisation due to syncope (see section 32.5 for corresponding ICD-10 codes) after randomisation from data received from NHS England at least 3 years post-randomisation. If it is not possible to link data from NHS England to a participant, the manual notes review will be used for that participant. From the manual notes review the date of earliest relevant admission will be used. Date of a hospitalisation due to syncope is defined as date of admission *(inpadat[1-25]_mnr)* following randomisation, where the reason for hospitalisation is given as syncope *(inpreas_mnr=7)*.

ii) **The count of all hospitalisations due to syncope** as defined in i) above by 3 years post-randomisation for each participant will be calculated. Any recorded beyond 3 years post-randomisation will not count towards this total. If event count is low, a binary indicator variable will be derived *(participant has hospitalisation due to syncope by 3 years post-randomisation (as defined in i) above) = 1, participant does not have hospitalisation due to syncope by 3 years post-randomisation (as defined in i) above) = 0)*. Where there is only a hospitalisation due to syncope recorded beyond 3 years post-randomisation this will be classed as not having had hospitalisation due to syncope by 3 years post-randomisation.

11 Hospitalisation due to hypotension

One of the secondary outcomes is to determine the difference in hospitalisations with hypotension between medication reduction and usual care. Time to first hospitalisation with hypotension as well as count of hospitalisations due to hypotension will be measured:

i) **Time to first hospitalisation due to hypotension** after randomisation will be measured using the date of first record of hospitalisation due to hypotension (see section 32.5 for corresponding ICD-10 codes) after randomisation from data received from NHS England at least 3 years post-randomisation. If it is not possible to link data from NHS England to a participant, the manual notes review will be used for that participant. From the manual notes review the date of earliest relevant admission will be used. Date of a hospitalisation due to hypotension is defined as date of admission *(inpadat[1-25]_mnr)* following randomisation, where the reason for hospitalisation is given as hypotension *(inpreas_mnr=8)*.

ii) **The count of all hospitalisations due to hypotension** as defined in i) above by years post-randomisation for each participant will be calculated. Any recorded beyond 3 years post-randomisation will not count towards this total. If event count is low, a binary indicator variable will be derived *(participant has hospitalisation due to hypotension by 3 years post-randomisation (as defined in i) above) = 1, participant does not have hospitalisation due to hypotension by 3 years post-randomisation (as defined in i) above) = 0)*. Where there is only a hospitalisation due to hypotension recorded beyond 3 years post-randomisation this will be classed as not having had hospitalisation due to hypotension by 3 years post-randomisation.

12 Hospitalisation due to fracture

One of the secondary outcomes is to determine the difference in hospitalisations due to fracture between medication reduction and usual care. Time to first hospitalisation due to fracture as well as count of hospitalisations due to fracture will be measured:

i) **Time to first hospitalisation due to fracture** after randomisation will be measured using the date of first record of hospitalisation due to fracture (see section 32.5 for corresponding ICD-10 codes) after randomisation from data received from NHS England at least 3 years post-randomisation. If it is not possible to link data from NHS England to a participant, the manual notes review will be used for that participant. From the manual notes review the date of earliest relevant admission will be used. Date of a hospitalisation due to fracture is defined as date of admission *(inpadat[1-25]_mnr)* following randomisation, where the reason for hospitalisation is given as fracture *(inpreas_mnr=9)*.

ii) **The count of all hospitalisations due to fracture** as defined in i) above by 3 years post-randomisation for each participant will be calculated. Any recorded beyond 3 years post-randomisation will not count towards this total. If event count is low, a binary indicator variable will be derived *(participant has hospitalisation due to fracture by 3 years post-randomisation (as defined in i) above) = 1, participant does not have hospitalisation due to fracture by 3 years post-randomisation (as defined in i) above) = 0)*. Where there is only a hospitalisation due to fracture recorded beyond 3 years post-randomisation this will be classed as not having had hospitalisation due to fracture by 3 years post-randomisation.

13 Hospitalisation due to electrolyte abnormalities

One of the secondary outcomes is to determine the difference in hospitalisations due to electrolyte abnormalities between medication reduction and usual care. Time to first hospitalisation due to electrolyte abnormalities as well as count of hospitalisations due to electrolyte abnormalities will be measured:

i) **Time to first hospitalisation due to electrolyte abnormalities** after randomisation will be measured using the date of first record of hospitalisation due to electrolyte abnormalities (see section 32.5 for corresponding ICD-10 codes) after randomisation from data received from NHS England at least 3 years post-randomisation. If it is not possible to link data from NHS England to a participant, the manual notes review will be used for that participant. From the manual notes review the date of earliest relevant admission will be used. Date of a hospitalisation due to electrolyte abnormalities is defined as date of admission *(inpadat[1-25]_mnr)* following randomisation, where the reason for hospitalisation is given as electrolyte abnormalities *(inpreas_mnr=10)*.

ii) **The count of all hospitalisations due to electrolyte abnormalities** as defined in i) above by 3 years post-randomisation for each participant will be calculated. Any recorded beyond 3 years post-randomisation will not count towards this total. If event count is low, a binary indicator variable will be derived *(participant has hospitalisation due to electrolyte abnormalities by 3 years post-randomisation (as defined in i) above) = 1, participant does not have hospitalisation due to electrolyte abnormalities by 3 years post-randomisation (as defined in i) above) = 0)*. Where there is only a hospitalisation

due to electrolyte abnormalities recorded beyond 3 years post-randomisation this will be classed as not having had hospitalisation due to electrolyte abnormalities by 3 years post-randomisation.

14 Diagnosis of dementia

One of the secondary outcomes is to determine the difference in diagnoses of dementia between medication reduction and usual care. Data received from the ORCHID database will be used. Time to diagnosis of dementia as well as diagnosis of dementia as a binary outcome will be measured:

iii) **Time to diagnosis of dementia** will use date of diagnosis of dementia (where diagnosis of dementia is listed)(see section 32.5 for corresponding ORCHID codes). Where the participant's practice is not registered to share data with the ORCHID database or if there are any other reasons that the participant's data is not available via ORCHID, date of diagnosis of dementia will be taken as whichever date of dementia diagnosis is earliest from either NHS England dataset or manual notes review if received for that participant. If only one of either the NHS England dataset or manual notes review are received for a participant, only that data will be used to derive this variable. Date of first record of admission with a diagnosis of dementia recorded from data received from NHS England at least 3 years post-randomisation (see section 32.5 for corresponding ICD-10 codes) after randomisation will be used. . Or in the manual notes review at least 3 years post-randomisation, date of diagnosis of dementia *(dmndt_mnr)*, where the participant has had a diagnosis of dementia *(dmnyn_mnr=yes)* will be used.

iv) **A binary variable for diagnosis of dementia** will be calculated using the date of diagnosis of dementia after randomisation from data received from ORCHID *(participant diagnosed with dementia at 3 years post-randomisation = 1, participant not diagnosed with dementia at 3 years post-randomisation = 0)*. Where the participant's practice is not registered to share data with the ORCHID database or if there are any other reasons that the participant's data is not available via ORCHID, date of diagnosis of dementia will be taken as whichever date of dementia diagnosis is earliest from either NHS England dataset or manual notes review if received for that participant. If only one of either the NHS England dataset or manual notes review are received for a participant, only that data will be used to derive this variable. . Any diagnosis of dementia beyond 3 years post-randomisation will be classed as not diagnosed with dementia at 3 years post-randomisation:

- Participant diagnosed with dementia at 3 years post-randomisation =1 if participant has been diagnosed with dementia (from NHS England dataset, ICD-10 code associated with dementia diagnosis is present OR from manual notes review: *dmnyn_mnr =yes)* and date of diagnosis of dementia (from NHS England dataset, date of first record of admission with a diagnosis of dementia recorded OR from manual notes review: *dmndt_mnr)* is ≤3 years post-randomisation
- Participant diagnosed with dementia at 3 years post-randomisation =0 if:
  - Participant has not been diagnosed with dementia (from NHS England dataset, no ICD-10 code associated with diagnosis of dementia AND from manual notes review: *dmnyn_mnr =no)*

    OR

- Participant has been diagnosed with dementia (from NHS England dataset, ICD-10 code associated with dementia diagnosis is present OR from manual notes review: *dmnyn_mnr =yes)* AND date of diagnosis (from NHS England dataset, date of first record of admission with a diagnosis of dementia recorded AND from manual notes review: *dmndt_mnr)* >3 years post-randomisation.

Where it is noted in the manual notes review that participant has been diagnosed with dementia and date of diagnosis of dementia (*dmndt_mnr)* is not known the date of diagnosis will be estimated as the date of the manual notes review.

15 Change of antihypertensive medication prescription from baseline

One of the secondary outcomes is to determine the difference in the change of antihypertensive medication prescription (from baseline) between medication reduction and usual care.

Data from the manual notes review (which was in turn imported using the main OPTiMISE trial database) will be used to provide the numbers of AHT medications prescribed at baseline. Number of total antihypertensive medications taken at baseline is defined as total number of AHT medications listed at baseline *(…_bl_name).*

Number of total AHT medications taken at follow-up will be found using data received from the ORCHID database. Current AHT medication prescriptions will be summed. Where the participant's practice is not registered to share data with the ORCHID database or if there are any other reasons that the participant's data is not available via ORCHID, then the manual notes review CRF data will be used. From the manual notes review, number of AHT medications taken at time of follow-up is defined as total number of AHT medications from the first 12 weeks of participation in the trial that are still prescribed *(…_stlpresc_mnr =1(yes))* plus any other currently prescribed AHT medications, *(ahttrt[1-11]_[1-2]_mnr)*

Change of antihypertensive medication prescription from baseline is calculated as:
Number of AHT medications prescribed at follow-up - Number of AHT medications prescribed at baseline

N.B. Some combined therapies count as more than one AHT medication (please see section 32.4 for details of how many AHT medications each therapy contains). Where an "other" AHT medication is recorded, not listed in section 32.4, a clinician will be consulted to inform how many AHT medications make up the therapy.

16 Maintain medication reduction

One of the secondary outcomes is to determine the proportion of patients in intervention arm who maintain medication reduction throughout follow-up. A binary variable (1=maintained medication reduction, 0=not maintained medication reduction) for those randomised to medication reduction will be used to analyse this. Maintaining medication reduction is defined as the total number of AHT medications at time of follow-up is less than the total number of AHT medications at baseline. The total number of AHT medications at baseline will be taken from the manual notes review list of AHT medications prescribed at baseline (*…_bl_name)*. Some AHT medications in combined therapies count as more than one AHT medication so names of medications must be checked to calculate how many AHT medications make up the therapy name. The total number of current AHT medications at

time of follow-up will be found using the total of current AHT medications at time of follow-up from ORCHID database. As above, special consideration needs to be given for combined therapies and how many AHT medications these are made up of.

Where the participant's practice is not registered to share data with the ORCHID database or if there are any other reasons that the participant's data is not available via ORCHID, then the manual notes review CRF data will be used to calculate the total number of AHT medications at time of follow-up. This will be calculated as:

> Number of medications prescribed at baseline *(…_bl_name)* where they are listed as still prescribed at time of follow-up *(…_stlpresc_mnr = 1 (Yes)).*

Plus

> Any other currently prescribed AHT medications *(ahttrt[1-11]_[1-2]_mnr)*

As above, special consideration needs to be given for combined therapies and how many AHT medications these are made up of.

In order to be defined as having maintained medication reduction, the total number of AHT medications at time of follow-up must be less than the total number of AHT medications at baseline. *N.B.* Some AHT medications in combined therapies count as more than one AHT medication so names of medications must be checked to calculate how many AHT medications make up the therapy name.

17 Number of all prescribed medications

One of the secondary outcomes is to determine the difference in all prescribed medications between medication reduction and usual care. Data received from the ORCHID database will be used. All currently prescribed (at time of follow-up) medications listed will be summed.

Where the participant's practice is not registered to share data with the ORCHID database or if there are any other reasons that the participant's data is not available via ORCHID, then the manual notes review CRF data at least 3 years post-randomisation will be used. The total will be calculated from the currently prescribed medications *(cmtrt[1-20]_mnr)* plus the number of total AHT medications taken at follow-up, as defined in section 15.

18 Change in mean clinic systolic blood pressure from baseline

One of the secondary outcomes is to determine the difference in the change in mean clinic systolic blood pressure (from baseline) between medication reduction and usual care.

Mean clinic systolic blood pressure at baseline is defined as the mean of the second and third systolic blood pressure readings at the baseline visit *(sys[2-3]_bl).* This value will be taken from the main trial analysis dataset.

Mean clinic systolic blood pressure at follow-up is defined as the systolic blood pressure measurement closest to 3 years post-randomisation found in the data received from the ORCHID database. Where the participant's practice is not registered to share data with the ORCHID database or if there are any other reasons that the participant's data is not available via ORCHID, then the manual notes review at follow-up will be used. The systolic blood pressure measurement recorded here *(sys[1-10]_mnr)* that is closest to the date 3 years post-randomisation will be used. Where there is more than one blood pressure measurement on the date that is closest to 3 years post-

randomisation, the mean of all of the systolic blood pressure measurements recorded on that date will be taken as the mean clinic systolic blood pressure at follow-up.
Change in mean clinic systolic blood pressure from baseline is calculated as:
Mean clinic systolic blood pressure at follow-up - Mean clinic systolic blood pressure at baseline

19 Change in mean clinic diastolic blood pressure from baseline

One of the secondary outcomes is to determine the difference in the change in mean clinic diastolic blood pressure (from baseline) between medication reduction and usual care.

Mean clinic diastolic blood pressure at baseline is defined as the mean of the second and third diastolic blood pressure readings at the baseline visit *(dia[2-3]_bl).* This value will be taken from the main trial analysis dataset.

Mean clinic systolic blood pressure at follow-up is defined as the diastolic blood pressure measurement closest to 3 years post-randomisation found in the data received from the ORCHID database. Where the participant's practice is not registered to share data with the ORCHID database or if there are any other reasons that the participant's data is not available via ORCHID, then the manual notes review at follow-up will be used. The diastolic blood pressure measurement recorded here *(dia[1-10]_mnr)* that is closest to the date 3 years post-randomisation will be used. Where there is more than one blood pressure measurement on the date that is closest to 3 years post-randomisation, the mean of all of the diastolic blood pressure measurements recorded on that date will be taken as the mean clinic diastolic blood pressure at follow-up.

Change in mean clinic diastolic blood pressure from baseline is calculated as:
Mean clinic diastolic blood pressure at follow-up - Mean clinic diastolic blood pressure at baseline

20 Controlled systolic blood pressure

One of the secondary outcomes is to determine the difference in the proportion of patients with clinically safe levels (defined as the proportion of patients with SBP <150mmHg) between medication reduction and usual care. A binary indicator will be created of whether the participant has systolic blood pressure below 150mmHg as the systolic blood pressure measurement closest to 3 years post-randomisation from the data received from the ORCHID database. Where the participant's practice is not registered to share data with the ORCHID database or if there are any other reasons that the participant's data is not available via ORCHID, then the manual notes review will be used. Systolic blood pressure measurement (*sys[1-10]_mnr*) closest to 3 years post-randomisation will be used to create the binary indicator in the same way. Where there is more than one blood pressure measurement on the date that is closest to 3 years post-randomisation, the mean of all of the systolic blood pressure measurements recorded on that date will be taken as the clinic systolic blood pressure at follow-up to calculate the binary indicator of controlled systolic blood pressure from.

21 Primary care consultations

One of the secondary outcomes is to determine the difference in primary care consultations relating to hypertension between medication reduction and usual care. Total primary care consultations, total GP visits, total practice nurse visits, total pharmacist visits and total 'other' visits relating to hypertension will all be calculated. These total numbers of visits will be calculated from summing each visit relating to hypertension from randomisation up until 3 years post-randomisation for each participant from the data received from the ORCHID database, split by each type of visit. Visit dates beyond 3 years post-randomisation for each participant will not count towards this total. 'Total

primary care consultations' will sum all of the primary care consultations relating to hypertension given, including all types of visit. Where the participant's practice is not registered to share data with the ORCHID database or if there are any other reasons that the participant's data is not available via ORCHID, then the manual notes review will be used:Total numbers of: GP visits *(gppn[1-25]_mnr = 1)*; Practice Nurse visits *(gppn[1-25]_mnr = 2)*; Pharmacist visits *(gppn[1-25]_mnr = 3)* and Other visits *(gppn[1-25]_mnr = 4)* where the date of visit (*gppndat[1-25]_mnr)* ≤3 years after randomisation date for each given participant and reason for the visit is "BP related" *(gppnreas[1-25]_mnr = 1)*, will be reported from the manual notes review at follow-up.

21.1.1 Non-protocol-specified outcomes

22 Controlled diastolic blood pressure

An additional analysis not specified in the protocol is to determine the difference in the proportion of patients with clinically safe levels (defined as the proportion of patients with DBP <90mmHg) between medication reduction and usual care. A binary indicator will be created of whether the participant has diastolic blood pressure below 90mmHg as the blood pressure measurement closest to 3 years post-randomisation from the data received from the ORCHID database. Where the participant's practice is not registered to share data with the ORCHID database or if there are any other reasons that the participant's data is not available via ORCHID, then the manual notes review will be used. The diastolic blood pressure measurement (*dia[1-10]_mnr*) closest to 3 years post-randomisation will be used to create the binary indicator in the same way. Where there is more than one blood pressure measurement on the date that is closest to 3 years post-randomisation, the mean of all of the diastolic blood pressure measurements recorded on that date will be taken as the clinic diastolic blood pressure at follow-up to calculate the binary indicator of controlled diastolic blood pressure from.

23 Increase/decrease/maintenance of antihypertensive medication

An additional outcome not specified in the protocol is to determine the proportion of patients in each arm who have reduced/maintained/increased the number of antihypertensive medications at 3 years follow-up compared with baseline. A categorical variable (1 = decreased number of antihypertensive medications, 2 = maintained number of antihypertensive medications, 3 = increased number of antihypertensive medications) for all participants in each treatment group will be used to analyse this.

These categories are defined as:

If number of AHT medications prescribed at follow-up - Number of AHT medications prescribed at baseline:

<0 : reduced antihypertensive medication (categorical variable = 1)

0 : maintained number of antihypertensive medications (categorical variable = 2)

>0 : increased number of antihypertensive medications (categorical variable = 3)

The total number of AHT medications at baseline will be taken from the manual notes review list of AHT medications prescribed at baseline (*…_bl_name)*. Some AHT medications in combined therapies count as more than one AHT medication so names of medications must be checked to calculate how

many AHT medications make up the therapy name. The total number of current AHT medications at time of follow-up will be found using the total of current AHT medications at time of follow-up from ORCHID database. As above, special consideration needs to be given for combined therapies and how many AHT medications these are made up of.

Where the participant's practice is not registered to share data with the ORCHID database or if there are any other reasons that the participant's data is not available via ORCHID, then the manual notes review CRF data will be used to calculate the total number of AHT medications at time of follow-up. This will be calculated as:

Number of medications prescribed at baseline *(…_bl_name*) where they are listed as still prescribed at time of follow-up *(…_stlpresc_mnr = 1 (Yes)).*

Plus

Any other currently prescribed AHT medications (*ahttrt[1-11]_[1-2]_mnr*)

As above, special consideration needs to be given for combined therapies and how many AHT medications these are made up of.

N.B. Some AHT medications in combined therapies count as more than one AHT medication so names of medications must be checked to calculate how many AHT medications make up the therapy name.

	Outcome	Data management and derivation of outcome	Database and variable used in the derivation:	If intended variables are not available due to not having received dataset that includes this participant, alternative manual notes review variables used:
Primary	**Time to death/all-cause hospitalisation**	Time to death or all-cause hospitalisation will be computed as time (in days) to date of death (if participant has died) or time to first hospitalisation, whichever is first. Date of censoring will be taken as date of follow-up or date of de-registration from practice (as applicable).	NHS England (see section 32.5 for corresponding ICD-10 codes to define these):<br>• Date of first hospitalisation for any reason<br>• Date of death<br>• Date of NHS England dataset download	Manual notes review:<br>• Date of first hospitalisation: inpadat[1-25]_mnr<br>• Date of death: dthdt_mnr<br>• Whether participant has died: dthyn_mnr<br>• Date of manual notes review completion: fu_dat_mnr<br>• Date of de-registration: alt_id_mnr
Secondary	i) Time to emergency hospitalisation<br>ii) Count of emergency hospitalisations	Time to emergency hospitalisation (where this is as an in-patient) will be computed as time (in days) to first admission that is classed as emergency admission. Date of censoring will be taken as date of follow-up or date of de-registration from practice (as applicable). Count of emergency hospitalisations will be computed as all hospitalisations classed as emergency hospitalisations within 3 years post-randomisation.	NHS England:<br>• Date of emergency hospitalisation(s) and date of corresponding admission(s).<br>• Date of NHS England dataset download	Manual notes review:<br>• Date of hospitalisation: inpadat[1-25]_mnr<br>• Method of admission: inpcode[1-25]_mnr (= 1-6 is emergency admission)<br>• Date of manual notes review completion: fu_dat_mnr<br>• Date of de-registration: alt_id_mnr

	Outcome	Data management and derivation of outcome	Database and variable used in the derivation:	If intended variables are not available due to not having received dataset that includes this participant, alternative manual notes review variables used:
	i) Time to all-cause death<br>ii) Risk of all-cause death	Time to all-cause death will be computed as time (in days) to death if they have died. Date of censoring will be taken as date of follow-up or date of de-registration from practice (as applicable). Participant will be classed as died or alive at 3 years post-randomisation.	NHS England (see section 32.5 for corresponding ICD-10 codes to define these):<br>• Whether participant has died<br>• Date of all-cause death<br>• Date of NHS England dataset download	Manual notes review:<br>• Date of death: dthdt_mnr<br>• Whether participant has died: dthyn_mnr (=yes if participant has died)<br>• Date of manual notes review completion: fu_dat_mnr<br>• Date of de-registration: alt_id_mnr
	i) Time to cardiovascular disease<br>ii) Count of cardiovascular disease	Time to cardiovascular disease will be computed as time (in days) to first hospitalisation or death due to cardiovascular disease (whichever is first). Date of censoring will be taken as date of follow-up or date of de-registration from practice (as applicable).<br>Count of cardiovascular disease will be computed as all hospitalisations and deaths due to cardiovascular disease within 3 years post-randomisation.	NHS England (see section 32.5 for corresponding ICD-10 codes to define these):<br>• Date of cardiovascular disease admission(s)<br>• Date of death due to cardiovascular disease<br>• Date of NHS England dataset download	Manual notes review:<br>• Date of hospitalisation: inpadat[1-25]_mnr<br>• Reason for hospitalisation: Inpreas[1-25]_mnr (=1-4 is cardiovascular disease)<br>• Date of death: dthdt_mnr<br>• Whether participant has died: dthyn_mnr (=yes if participant has died)<br>• Cause of death: dthcause_mnr (=1-4 is cardiovascular disease)<br>• Date of manual notes review completion: fu_dat_mnr<br>• Date of de-registration: alt_id_mnr

	Outcome	Data management and derivation of outcome	Database and variable used in the derivation:	If intended variables are not available due to not having received dataset that includes this participant, alternative manual notes review variables used:
	i) Time to stroke<br>ii) Count of stroke	Time to stroke will be computed as time (in days) to first hospitalisation or death due to stroke (whichever is first). Date of censoring will be taken as date of follow-up or date of de-registration from practice (as applicable). Count of stroke will be computed as all hospitalisations and deaths due to stroke within 3 years post-randomisation.	NHS England (see section 32.5 for corresponding ICD-10 codes to define these):<br>• Date of stroke(s)<br>• Date of death due to stroke<br>• Date of NHS England dataset download	Manual notes review:<br>• Date of hospitalisation: inpadat[1-25]_mnr<br>• Reason for hospitalisation: Inpreas[1-25]_mnr (=2 is stroke)<br>• Date of death: dthdt_mnr<br>• Whether participant has died: dthyn_mnr (=yes if participant has died)<br>• Cause of death: dthcause_mnr (=2 is stroke)<br>• Date of manual notes review completion: fu_dat_mnr<br>• Date of de-registration: alt_id_mnr

	Outcome	Data management and derivation of outcome	Database and variable used in the derivation:	If intended variables are not available due to not having received dataset that includes this participant, alternative manual notes review variables used:
	i) Time to myocardial infarction.<br>ii) Count of myocardial infarctions	Time to myocardial infarction will be computed as time (in days) to first hospitalisation or death due to myocardial infarction (whichever is first). Date of censoring will be taken as date of follow-up or date of de-registration from practice (as applicable).<br>Count of myocardial infarction will be computed as all hospitalisations and deaths due to myocardial infarction within 3 years post-randomisation.	NHS England (see section 32.5 for corresponding ICD-10 codes to define these):<br>• Date of myocardial infarction(s)<br>• Date of death due to myocardial infarction<br>• Date of NHS England dataset download	Manual notes review:<br>• Date of hospitalisation: inpadat[1-25]_mnr<br>• Reason for hospitalisation: Inpreas[1-25]_mnr (=1 is myocardial infarction)<br>• Date of death: dthdt_mnr<br>• Whether participant has died: dthyn_mnr (=yes if participant has died)<br>• Cause of death: dthcause_mnr (=1 is myocardial infarction)<br>• Date of manual notes review completion: fu_dat_mnr<br>• Date of de-registration: alt_id_mnr
	i) Time to hospitalisation due to falls<br>ii) Count of hospitalisations due to falls	Time to hospitalisation due to falls will be computed as time (in days) to first hospitalisation due to falls. Date of censoring will be taken as date of follow-up or date of de-registration from practice (as applicable).<br>Count of hospitalisations due to falls will be computed as all hospitalisations due to falls within 3 years post-randomisation	NHS England (see section 32.5 for corresponding ICD-10 codes to define these):<br>• Date of hospitalisation(s) due to fall<br>• Date of NHS England dataset download	Manual notes review:<br>• Date of hospitalisation: inpadat[1-25]_mnr<br>• Reason for hospitalisation: Inpreas[1-25]_mnr (=5 is due to fall)<br>• Date of manual notes review completion: fu_dat_mnr<br>• Date of de-registration: alt_id_mnr

	Outcome	Data management and derivation of outcome	Database and variable used in the derivation:	If intended variables are not available due to not having received dataset that includes this participant, alternative manual notes review variables used:
	i) Time to hospitalisation due to acute kidney injury<br>ii) Count of hospitalisations due to acute kidney injury	Time to hospitalisation due to acute kidney injury will be computed as time (in days) to first hospitalisation due to acute kidney injury. Date of censoring will be taken as date of follow-up or date of de-registration from practice (as applicable).<br>Count of hospitalisations due to acute kidney injury will be computed as all hospitalisations due to acute kidney injury within 3 years post-randomisation	NHS England (see section 32.5 for corresponding ICD-10 codes to define these):<br>• Date of hospitalisation(s) due to acute kidney injury<br>• Date of NHS England dataset download	Manual notes review:<br>• Date of hospitalisation: inpadat[1-25]_mnr<br>• Reason for hospitalisation: Inpreas[1-25]_mnr (=6 is acute kidney failure)<br>• Date of manual notes review completion: fu_dat_mnr<br>• Date of de-registration: alt_id_mnr
	i) Time to hospitalisation due to syncope<br>ii) Count of hospitalisations due to syncope	Time to hospitalisation due to syncope will be computed as time (in days) to first hospitalisation due to syncope. Date of censoring will be taken as date of follow-up or date of de-registration from practice (as applicable).<br>Count of hospitalisations due to syncope will be computed as all hospitalisations due to syncope within 3 years post-randomisation	NHS England (see section 32.5 for corresponding ICD-10 codes to define these):<br>• Date of hospitalisation(s) due to syncope<br>• Date of NHS England dataset download	Manual notes review:<br>• Date of hospitalisation: inpadat[1-25]_mnr<br>• Reason for hospitalisation: Inpreas[1-25]_mnr (=7 is syncope)<br>• Date of manual notes review completion: fu_dat_mnr<br>• Date of de-registration: alt_id_mnr

	Outcome	Data management and derivation of outcome	Database and variable used in the derivation:	If intended variables are not available due to not having received dataset that includes this participant, alternative manual notes review variables used:
	i) Time to hospitalisation due to hypotension<br>ii) Count of hospitalisations due to hypotension	Time to hospitalisation due to hypotension will be computed as time (in days) to first hospitalisation due to hypotension. Date of censoring will be taken as date of follow-up or date of de-registration from practice (as applicable).<br>Count of hospitalisations due to hypotension will be computed as all hospitalisations due to hypotension within 3 years post-randomisation	NHS England (see section 32.5 for corresponding ICD-10 codes to define these):<br>• Date of hospitalisation(s) due to hypotension<br>• Date of NHS England dataset download	Manual notes review:<br>• Date of hospitalisation: inpadat[1-25]_mnr<br>• Reason for hospitalisation: Inpreas[1-25]_mnr (=8 is hypotension)<br>• Date of manual notes review completion: fu_dat_mnr<br>• Date of de-registration: alt_id_mnr
	i) Time to hospitalisation due to fracture<br>ii) Count of hospitalisations due to fracture	Time to hospitalisation due to fracture will be computed as time (in days) to first hospitalisation due to fracture. Date of censoring will be taken as date of follow-up or date of de-registration from practice (as applicable).<br>Count of hospitalisations due to fracture will be computed as all hospitalisations due to fracture within 3 years post-randomisation	NHS England (see section 32.5 for corresponding ICD-10 codes to define these):<br>• Date of hospitalisation(s) due to fracture<br>• Date of NHS England dataset download	Manual notes review:<br>• Date of hospitalisation: inpadat[1-25]_mnr<br>• Reason for hospitalisation: Inpreas[1-25]_mnr (=9 is fracture)<br>• Date of manual notes review completion: fu_dat_mnr<br>• Date of de-registration: alt_id_mnr

	Outcome	Data management and derivation of outcome	Database and variable used in the derivation:	If intended variables are not available due to not having received dataset that includes this participant, alternative manual notes review variables used:
	iii) Time to hospitalisation due to electrolyte abnormalities<br>iv) Count of hospitalisations due to electrolyte abnormalities	Time to hospitalisation due to electrolyte abnormalities will be computed as time (in days) to first hospitalisation due to electrolyte abnormalities. Date of censoring will be taken as date of follow-up or date of de-registration from practice (as applicable).<br>Count of hospitalisations due to electrolyte abnormalities will be computed as all hospitalisations due to electrolyte abnormalities within 3 years post-randomisation	NHS England (see section 32.5 for corresponding ICD-10 codes to define these):<br>• Date of hospitalisation(s) due to electrolyte abnormalities<br>• Date of NHS England dataset download	Manual notes review:<br>• Date of hospitalisation: inpadat[1-25]_mnr<br>• Reason for hospitalisation: Inpreas[1-25]_mnr (=10 is electrolyte abnormalities)<br>• Date of manual notes review completion: fu_dat_mnr<br>• Date of de-registration: alt_id_mnr
	i) Time to diagnosis of dementia<br>ii) Risk of diagnosis of dementia	Time to diagnosis of dementia will be computed as time (in days) to diagnosis of dementia. Date of censoring will be taken as date of follow-up or date of de-registration from practice (as applicable).<br>Participant will be classed as diagnosed with dementia or not at 3 years post-randomisation.	ORCHID:<br>• Diagnosis of dementia<br>• Date of diagnosis of dementia<br>• Date of NHS England dataset download	Manual notes review:<br>• Date of diagnosis of dementia: dmndt_mnr<br>• Diagnosis of dementia: dmnyn_mnr (=yes is participant has diagnosis of dementia since randomisation)<br>• Date of manual notes review completion: fu_dat_mnr<br>• Date of de-registration: alt_id_mnr

	Outcome	Data management and derivation of outcome	Database and variable used in the derivation:	If intended variables are not available due to not having received dataset that includes this participant, alternative manual notes review variables used:
	Change of antihypertensive medication prescription from baseline	Change of antihypertensive medication prescription from baseline will use the sum of antihypertensive medication prescription at baseline and the sum of antihypertensive medication prescription at the time of 3 year follow-up	Manual notes review:<br>• Name of AHT medication at baseline: …bl_name<br>ORCHID:<br>• AHT medication names currently prescribed at time of 3 year follow-up	Manual notes review:<br>• AHT medications from the first 12 weeks of participation in the trial are still prescribed: …_stlpresc_mnr (=1(yes) is that the AHT medication is still currently prescribed)<br>• Any other currently prescribed AHT medications: ahttrt[1-11]_[1-2]_mnr
	Maintaining medication reduction at 3 years follow-up	Maintaining medication reduction at 3 years follow-up will be defined as the total number of AHT medications at time of 3 year follow-up is less than the total number of AHT medications at baseline	Manual notes review:<br>• Name of AHT medications at baseline: …_bl_name<br><br>ORCHID:<br>• AHT medication names currently prescribed at time of 3 year follow-up	Manual notes review:<br>• Whether the participant is still prescribed AHT medication from baseline: …_stlpresc_mnr (= 1(yes) is that the AHT medication is still currently prescribed)<br>• Name of AHT medications at baseline: …_bl_name<br>• Name of 'other' (not taken at baseline) AHT medications current at time of 3 year follow-up: ahttrt[1-11]_[1-2]_mnr

	Outcome	Data management and derivation of outcome	Database and variable used in the derivation:	If intended variables are not available due to not having received dataset that includes this participant, alternative manual notes review variables used:
	Number of all prescribed medications	Number of all prescribed medications will use sum of all currently prescribed medications at the time of 3 year follow-up	ORCHID data:<br>• All currently prescribed medication names	Manual notes review:<br>• Currently prescribed medications (not AHT medications): cmtrt[1-20]_mnr<br>• AHT medications from first 12 weeks of trial that are still prescribed: …_stlpresc_mnr =1(yes)<br>• Name of AHT medications at baseline: …_bl_name<br>• Other currently prescribed AHT medications: ahttrt[1-11]_[1-2]_mnr

	Outcome	Data management and derivation of outcome	Database and variable used in the derivation:	If intended variables are not available due to not having received dataset that includes this participant, alternative manual notes review variables used:
	Change in mean clinic systolic blood pressure from baseline	Change in mean clinic systolic blood pressure from baseline will use mean of second and third baseline systolic blood pressure values and systolic blood pressure value closest to 3 years post-randomisation	Baseline or rescreening CRF from original OPTiMISE trial database:<br>• Second and third systolic blood pressure readings at baseline or rescreening visit: sys[2-3]_bl or sys[2-3]_rs (taken from main trial analysis dataset)<br>ORCHID data:<br>• Systolic blood pressure value closest to 3 years post-randomisation	Manual notes review:<br>• Systolic blood pressure closest to 3 years post-randomisation: sys[1-10]_mnr

	Outcome	Data management and derivation of outcome	Database and variable used in the derivation:	If intended variables are not available due to not having received dataset that includes this participant, alternative manual notes review variables used:
	Change in mean clinic diastolic blood pressure from baseline	Change in mean clinic diastolic blood pressure from baseline will use mean of second and third baseline diastolic blood pressure values and diastolic blood pressure value closest to 3 years post-randomisation	Baseline or rescreening CRF from original OPTiMISE trial database:<br>• Second and third diastolic blood pressure readings at baseline or rescreening visit: dia[2-3]_bl or dia[2-3]_rs (taken from main trial analysis dataset)<br>ORCHID data:<br>• Diastolic blood pressure value closest to 3 years post-randomisation	Manual notes review:<br>• Diastolic blood pressure closest to 3 years post-randomisation:<br><br>dia[1-10]_mnr
	Controlled systolic blood pressure	Controlled systolic blood pressure will use the systolic blood pressure value closest to 3 years post-randomisation to determine if participant had controlled systolic blood pressure (<150mmHg) or not.	ORCHID data:<br>Systolic blood pressure value closest to 3 years post-randomisation	Manual notes review:<br>• Systolic blood pressure closest to 3 years post-randomisation:<br><br>sys[1-10]_mnr

	Count of primary care consultations	Number of primary care consultations relating to hypertension will be the sum of all primary care consultations relating to hypertension since randomisation, collected at 3 year follow-up.	ORCHID data:<br>• Number of GP visits relating to hypertension from randomisation to 3 years post-randomisation<br>• Number of practice nurse visits relating to hypertension from randomisation to 3 years post-randomisation<br>• Number of pharmacist visits relating to hypertension from randomisation to 3 years post-randomisation<br>• Number of 'other' (not GP/practice nurse/pharmacist) visits relating to hypertension from randomisation to 3 years post-randomisation	Manual notes review:<br><br>GP visits:<br>gppn[1-25]_mnr = 1 (GP)<br>Practice Nurse visits:<br>gppn[1-25]_mnr = *2 (practice nurse)*<br>Pharmacist visits:<br>gppn[1-25]_mnr = *3 (pharmacist)*<br>and Other visits:<br>gppn[1-25]_mnr = *4 ("other" type of visit)*<br><br>Date of visit:<br>gppndat[1-25]_mnr<br>Reason for visit:<br>gppnreas[1-25]_mnr = 1 *(BP related)*

	Outcome	Data management and derivation of outcome	Database and variable used in the derivation:	If intended variables are not available due to not having received dataset that includes this participant, alternative manual notes review variables used:
**Non-protocol-specified outcomes**	Controlled diastolic blood pressure	Controlled diastolic blood pressure will use the diastolic blood pressure value closest to 3 years post-randomisation to determine if participant had controlled diastolic blood pressure (<90mmHg) or not.	ORCHID data:<br>Diastolic blood pressure value closest to 3 years post-randomisation	Manual notes review:<br>• Diastolic blood pressure closest to 3 years post-randomisation:<br><br>dia[1-10]_mnr
	Increase/decrease/maintenance of antihypertensive medications	Change in number of antihypertensive medication from baseline will be categorised into increased number of AHT medications, maintained number of AHT medications and reduced number of AHT medications.	Manual notes review:<br>• Name of AHT medications at baseline:<br>…_bl_name<br>ORCHID:<br>AHT medication names currently prescribed at time of 3 year follow-up	Manual notes review:<br>• Whether the participant is still prescribed AHT medication from baseline:<br>…_stlpresc_mnr (= 1(yes) is that the AHT medication is still currently prescribed)<br>• Name of AHT medications at baseline:<br>…_bl_name<br>• Name of ‘other’ (not taken at baseline) AHT medications current at time of 3 year follow-up:<br><br>ahttrt[1-11]_[1-2]_mnr

23.1 Target population

For this long term follow-up of OPTiMISE trial participants, the population is the same as that for the main OPTiMISE trial, except that those participants who expressed explicitly that they no longer wanted data collection about them to take place are excluded. Participants are given the opportunity to again opt out of the further data collection for the long term follow-up, otherwise their continued participation is assumed. Where participants are deemed by their GP to potentially be lacking capacity, consultees must agree that the participant can remain in the study for the long term follow-up. Inclusion and exclusion criteria for the main OPTiMISE trial are listed below:

Inclusion criteria:

- Participant is willing and able to give informed consent for participation in the trial.
- Male or Female, aged 80 years or above.
- Clinic systolic blood pressure less than 150 mmHg (according to screening measurement at baseline – clinic blood pressure defined as the mean of the 2nd and 3rd readings taken at 1 minute intervals).
- Prescribed two or more antihypertensive medications to lower blood pressure for at least 12 months prior to trial entry. Antihypertensive medications defined as any ACE inhibitor, angiotensin II receptor blocker, calcium channel blocker, thiazide and thiazide-like diuretic, potassium-sparing diuretic, alpha-blocker,beta-blocker, vasodilator antihypertensives, centrally acting antihypertensives, direct renin inhibitors, adrenergic neurone blocking drugs or loop diuretics.
- Stable dose of antihypertensive medications for at least four weeks prior to trial entry.
- In the Investigator's opinion, could potentially benefit from medication reduction due to existing polypharmacy, co-morbidity, non-adherence or dislike of medicines and/or frailty (i.e. is different from those to which the results of the SPRINT trial are likely to apply)*
- In the Investigator's opinion, is able and willing to comply with all trial requirements.

*GPs will be given training from the research team during the site initiation visit on the findings of the SPRINT trial and other relevant trials and how these apply to patients in their practice.

Exclusion criteria

The participant may not enter the trial if ANY of the following apply:

- A participant has heart failure due to left ventricular systolic dysfunction (LVSD) and is on only ACE inhibitors/ARBs and/or beta-blockers and/or spironolactone (removing any of which would be contraindicated).
- A participant has heart failure but has not had an echocardiogram since its onset (might have undiagnosed LVSD and a compelling need for ACEI/ARB and Betablockers).
- Investigator deems that there is a compelling indication for medication continuation.
- Suffered a myocardial infarction or stroke within the past 12 months.
- Blood pressure being managed outside of primary care.
- A participant with secondary hypertension.
- A participant with previous accelerated or malignant hypertension.
- Unable to provide consent due to incapacity.
- Any other significant disease or disorder which, in the opinion of the Investigator, may either put the participants at risk because of participation in the trial, or may influence the result of the trial, or the participant's ability to participate in the trial (e.g. terminal illness, house bound and unable to attend baseline and follow up clinics).
- Participants who have participated in another research trial involving antihypertensive medication in the past 4 weeks.

23.2 Sample size

The sample to be included in the long term follow-up is the number of participants included in the main OPTiMISE trial minus anyone who has expressed that they no longer wish to have data collected about them or their consultee does not give agreement for data collection (as described in section 23.1). Sample size was calculated to answer the research questions from the main OPTiMISE trial and details can be found in the OPTiMISE main trial SAP.

23.3 Randomisation and blinding in the analysis stage

Details about the randomisation in the OPTiMISE main trial can be found in the OPTiMISE main trial SAP. The main OPTiMISE trial analysis has already taken place and results have been unblinded, but as far as possible, analysis will be done with trial statistician blinded to randomisation allocation. This will not be possible for analyses involving only one treatment group.

24 Analysis – General considerations

24.1 Descriptive statistics

Frequencies (with percentages) for binary and categorical variables and means (and standard deviations), or medians (with lower and upper quartiles) for continuous variables will be presented by intervention group as well as overall.

24.2 Characteristics of participants

Although baseline demographics of the main OPTiMISE trials have already been reported, baseline demographics of the long term follow-up analysis population will be reported, as this is expected to be a subsample of the main trial analysis population (as explained in section 23.1).

Summary statistics of the following baseline characteristics will be reported: sex, age (years), ethnicity, education, smoking status, alcohol consumption, standing SBP (3 mins), orthostatic hypotension, total cholesterol, HDL cholesterol, estimated eGFR, length of time patient has high blood pressure (years), height (cm), weight (kg), BMI (kg/m2), systolic blood pressure (mmHg), diastolic blood pressure (mmHg), modified Rankin Score, Cognitive Function (MoCA Score), Searle Frailty Index, eFrailty Index, Morley Frailty Score, EQ-5D-5L VAS, and EQ-5D-5L index, number of antihypertensives.

There will be no tests of statistical significance nor confidence intervals for the differences between randomised groups for any baseline variable, as any observed discrepancies in baseline characteristics between the randomised groups will be entirely due to chance. For prognostic baseline factors found to be markedly different between randomised groups (a difference between means of at least two standard deviations), a sensitivity analysis will be performed where these characteristics, if not already included, are included as additional covariates in the models for the analysis of the primary outcome. Due to the relative large sample size, these types of discrepancies are not expected.

24.3 Definition of population for analysis

After randomisation, participants will be analysed according to their allocated intervention group irrespective of what intervention they actually receive. As in the original OPTiMISE trial analysis, a per-protocol analysis of the primary outcome will also be undertaken as a secondary analysis.

24.4 Pooling of investigational sites

Clusters will consist of GP practices. Those practices with two or less participants will be pooled into an “other” GP practice for the purpose of the analyses by mixed effects models. If proposed mixed effects models fail to converge, other models (not mixed effects) will be used and GP practice will be omitted from the model.

24.5 Data Monitoring Committee And Interim Analyses

There will be no DMEC meetings or interim analyses during the long term follow-up of the OPTiMISE trial.

25 PRIMARY ANALYSIS

25.1 Primary outcome

Time to death/all-cause hospitalisation: Frailty Cox proportional hazards model, adjusting for randomised group, baseline systolic blood pressure and any covariates predictive of missingness (see section 25.2) as fixed effects and including practice as a random effect. However, if a random effect is not converging then a Cox proportional hazards model (without mixed effects) will be used with the same fixed effects.

A sensitivity analysis will analyse count of events by means of a generalised linear Poisson mixed effects model, adjusting for randomised group, baseline systolic blood pressure and including practice as a random effect. However, if problem of over dispersion occurred or where event counts are considered low, this will be analysed as a binary outcome instead, using generalised linear logistic regression mixed effects models, adjusting for randomised group, baseline systolic blood pressure and including practice as a random effect. As for other count outcomes, an event will only contribute to analysis if it occurred by 3 years post-randomisation.

25.2 Handling missing data

The availability of the outcome data for the primary outcome will be summarised overall and by randomised group. Participants will be censored at time of follow-up if the event has not occurred at time of follow-up.

The data missingness mechanism will be explored. Logistic regression models will explore any association between baseline characteristics (as listed in section 3.2) and availability of the primary outcome. Covariates found to be predictive of missingness ($P< 0.05$) will be included in the primary outcome analysis model. Should any covariate (to be included in the model) have missing baseline data, the overall mean of the covariate at baseline will replace the missing values[73] to enable all randomised participants with outcome data to be included in the analysis.

25.3 Handling outliers

Any outliers will be checked and verified to ensure that they are true values. Outliers will be identified as those observations three or more standard deviations from the mean. Once they have been confirmed, a sensitivity analysis will be carried out to assess the impact of these values on the results by excluding these participants.

25.4 Handling multi-centre/clustered data

Data will be clustered by GP practice. Random effects will be used to account for clustering at the GP practice level. However, if the model fails then a model not adjusting for GP practice will be used.

25.5 Multiple comparisons and multiplicity

The primary outcome is clearly stated in the protocol and no adjustments for multiple comparisons will be made for the secondary outcomes.   Any secondary outcomes that achieved statistical significance should be interpreted with caution.

25.6 Model assumptions

Standard residual diagnostics such as visual inspection of graphical representations of residuals/deviance residuals after fitting the model will be used to assess the assumption that the model fits the data well. The assumption of proportional hazards will be examined using residuals against survival time. If assumptions are violated then other, more appropriate models will be considered.

26 SECONDARY ANALYSIS

26.1 Per-protocol analysis of primary outcome

As a secondary analysis of the primary outcome, a per-protocol (PP) analysis will be performed. This will use the same methods of deriving the PP sample as the main trial used for the secondary PP analysis, i.e. participants who received the medication reduction intervention in the PP analysis will be defined as a participant in the medication reduction arm who maintained their medication reduction throughout the 12 week follow-up period. Those in the intervention arm who do not maintain medication reduction for the 12 week follow-up period will be excluded.

The analysis will proceed in the same way as the primary analysis outlined in section 25.1 but applied to the PP population.

26.2 Time-to-event outcomes

Time-to-event secondary outcomes will be analysed in the same way as the primary outcome. A mixed effects Cox proportional hazards model will be used, adjusting for randomised group, baseline systolic blood pressure and including practice as a random effect. Events will be included in the analysis regardless of when they occurred during follow-up.
These outcomes are:

- Emergency hospitalisation,
- All-cause death,
- Hospitalisation or death with:
    - Cardiovascular disease (defined as myocardial infarction, stroke, or heart failure)
    - Stroke;
    - Myocardial infarction,
- Hospitalisation due to:
    - Falls;
    - Acute kidney injury;
    - Syncope;
    - Hypotension;
    - Fracture;
    - Electrolyte abnormalities,
- A diagnosis of dementia.

26.3 Count outcomes

Where more than one of any one of the events for the secondary outcomes is plausible, as far as possible, these primary and secondary outcomes will also be analysed as counts of events by means of a generalised linear Poisson mixed effects model, adjusting for randomised group, baseline systolic blood pressure and including practice as a random effect. However, if problem of over dispersion occurred or where event counts are considered low, these outcomes will be analysed as binary outcomes instead, using generalised linear logistic regression mixed effects models, adjusting for randomised group, baseline systolic blood pressure and including practice as a random effect. For outcomes with very low event rate where covariate adjustment is not possible, then unadjusted analysis will be performed. Only participants who were still alive at 2 years 6 months will be included in the count analyses. For binary outcomes analyses, only those who did not have the event of interest and who died before 2 years 6 months post-randomisation will be excluded from the analysis (those who had the event but died sooner than 2 years 6 months will still be included in the analysis as having had the event).
These outcomes are:

- Emergency hospitalisation,
- Hospitalisation or death with:
    - Cardiovascular disease (defined as myocardial infarction, stroke, or heart failure)
    - Stroke;
    - Myocardial infarction,
- Hospitalisation due to:
    - Falls;
    - Acute kidney injury;
    - Syncope;
    - Hypotension;
    - Fracture;
    - Electrolyte abnormalities

26.4 Binary outcomes

Where only one event for a secondary outcome is possible, these outcomes will be analysed using generalised linear logistic regression mixed effects models, adjusting for randomised group, baseline systolic blood pressure and including practice as a random effect. Risk ratio will then be derived from the model. Participants will be excluded from the analysis of odds of diagnosis of dementia if they did not have a diagnosis of dementia and they died before 2 years 6 months post-randomisation (those who had a diagnosis of dementia but died sooner than 2 years 6 months will still be included in the analysis as having had the event).

These outcomes are:

- all-cause death
- diagnosis of dementia

26.5 Blood pressure outcomes

Three year outcomes related to systolic blood pressure control and change in blood pressure will be analysed using the same analytical models that were agreed to examine these outcomes at 12 week follow-up. Only participants who have blood pressure measurements collected between 2 years 6 months and 3 years 6 months will be included in these analyses.

These outcomes and methods are:

- **Change in mean clinic systolic blood pressure:** Linear mixed effects model where systolic blood pressure closest to 3 years post-randomisation is the outcome, and where baseline systolic blood pressure is specified as a covariate (fixed effect). The blood pressure measurement at baseline will be taken as the average of the second and third measurements. The model will also be adjusted for randomised group (fixed effect) and including practice as a random effect.
- **Change in mean clinic diastolic blood pressure:** Linear mixed effects model where diastolic blood pressure closest to 3 years post-randomisation is the outcome, and where baseline diastolic blood pressure and systolic blood pressure are specified as covariates (fixed effects). The blood pressure measurement at baseline will be taken as the average of the second and third measurements. The model will also be adjusted for randomised group and including practice as a random effect.
- **Proportion of patients with controlled systolic blood pressure**: The relative risk and its confidence interval will be obtained by means of a generalised linear logistic regression mixed effects model, which will be used to derive the corresponding risk ratio. The response will be binary indicator of whether the person has a systolic blood pressure below 150mmHg at 3 years. GP practice will be included in the model as a random effect. Adjustment will be made for baseline systolic blood pressure and randomised group by including them as fixed effects. In addition, covariates found to be predictive of missingness will be included in the model.

Proportion of patients with controlled diastolic blood pressure (non-protocol-specified outcome) will be analysed in the same way as for the proportion of patients with controlled systolic blood pressure above: The relative risk and its confidence interval will be obtained by means of a generalised linear logistic regression mixed effects model, which will be used to derive the corresponding risk ratio. The response will be binary indicator of whether the person has a diastolic blood pressure below 90mmHg at 3 years. GP practice will be included in the model as a random effect. Adjustment will be made for baseline diastolic blood pressure and randomised group by including them as fixed effects. In addition, covariates found to be predictive of missingness will be included in the model.

26.6 Maintenance of medication reduction

The proportion of participants found to have maintained medication reduction who were alive at time of follow-up will be calculated as a proportion of those in the intervention arm alive at follow-up.

26.7 Count of primary care consultations

The difference between the intervention and usual care arms for the count of primary care consultations relating to hypertension (reported by staff type) will be analysed by means of a generalised linear Poisson mixed effects model, adjusting for randomised group, baseline systolic blood pressure and including practice as a random effect. Only participants who were still alive at 2 years 6 months will be included in these analyses. If zero inflation or overdispersion occurs, a more appropriate model will be used instead. For the issue of zero inflation, a zero-inflated generalised linear Poisson regression model will be used, combining Poisson count model with the same covariates and random effect as above along with logit model with the same covariates, for

predicting excess zeros. For over-dispersion without zero-inflation a generalised linear negative binomial mixed effects model, with the same covariates and random effect as above will be used. If both zero inflation and overdispersion occurs a zero-inflated generalised linear negative binomial mixed effects model will be used, combining negative binomial model with the same covariates and random effect as above along with logit model with the same covariates, for predicting excess zeros.

26.8 Change of antihypertensive medication prescription

The difference between the intervention and usual care arms for the change in antihypertensive medication prescription (from baseline) will be analysed by means of a generalised linear mixed effects model, adjusting for randomised group, baseline systolic blood pressure, baseline antihypertensive medication prescription and including practice as a random effect. If participant died at any time before date of follow-up, they will not be included in this analysis.

26.9 Difference in all prescribed medications

The difference between the intervention and usual care arms for the number of prescribed medications at 3 year follow-up will be analysed by means of linear mixed effects model, adjusting for randomised group, baseline systolic blood pressure and including practice as a random effect. If participant died at any time before date of follow-up, they will not be included in this analysis.

26.10 Increase/decrease/maintenance of antihypertensive medication (non-protocol-specified outcome)

Descriptive statistics will be presented. The odds ratios of having increased/maintained/decreased antihypertensive medications at 3 years follow-up compared with baseline between intervention and usual care arms will be analysed by means of ordinal logistic mixed effects model, adjusting for randomised group, baseline systolic blood pressure and including practice as a random effect. If this model does not converge a simpler non-mixed effects model will be used. If participant died at any time before date of follow-up, they will not be included in this analysis.

27 SENSITIVITY ANALYSIS

Secondary analysis of primary outcome using per-protocol sample

Sensitivity analyses of the secondary per-protocol (PP) analyses of the primary outcome using the same methods of deriving the per-protocol samples included in the main trial will be performed, i.e. "A PP analysis is based on a response of "yes" to the question on maintaining medication reduction (*maintain_fu*). In the unlikely event that this outcome is coded as missing it will be assumed that maintenance of medication reduction has failed, and this participant will not be included in the PP analysis. A sensitivity analysis will be performed where all missing responses are coded as "yes", unless the participant has died, and the PP analysis will be repeated using the imputed *maintain_fu* variable. The logic for this analysis is that anyone who had a medication change would have needed to see their GP to get a new prescription."

Blood pressure outcomes

Descriptive statistics of numbers of participants in each group included in the blood pressure outcomes analyses will be shown, including details of numbers of participants with only blood pressure measurements before or after randomisation as well as those without any blood pressure measurements.

As a sensitivity analysis those participants who died before 2 years 6 months post-randomisation will also be included in the analysis using their last taken blood pressure measurement.

As a further sensitivity analysis those participants who died before 2 years 6 months post-randomisation will be considered to have had uncontrolled blood pressure and also included in the analysis. Uncontrolled blood pressure will be defined as the highest blood pressure measurement recorded in the observed dataset for measurements taken between 2 years 6 months and 3 years 6 months post-randomisation. It will also be checked whether those participants in the control group had particularly high or low blood pressure during this time. If low blood pressure is observed, this sensitivity analysis will also be carried out with 'uncontrolled blood pressure' for those who died before 2 years 6 months post-randomisation defined as the lowest blood pressure measurement recorded in the observed dataset for measurements taken between 2 years 6 months and 3 years 6 months post-randomisation.

Another sensitivity analysis of all of the blood pressure outcomes will take the mean of the three closest blood pressure readings to 3 years post-randomisation instead of the one reading closest to 3 years post-randomisation as the mean clinic blood pressure at follow-up. At least one of the blood pressure measurements must be from between 2 years 6 months and 3 years 6 months post-randomisation for the participant to be included in this analysis. If multiple blood pressure measurements were taken on one of the contributing dates closest to 3 years post-randomisation, all of the blood pressure measurements from this day will contribute to the mean clinic blood pressure at follow-up, even if this takes the total contributing blood pressure measurements beyond three.

<u>All outcomes</u>

For baseline characteristics found to be markedly different between randomised groups (a difference between means of at least two standard deviations), a sensitivity analysis will be performed where these characteristics are not already included, are included as additional covariates in the models for the analysis of the secondary outcomes. Due to the relatively large sample size and that no such differences were found during the main trial analysis[72], these types of discrepancies are not expected.

<u>Medication reduction</u>

As a sensitivity analysis, participants who died before 2 years 6 months will also be included in the calculated proportion of participants in the intervention arm who maintained medication reduction, where this data is available.

28 SUBGROUP ANALYSES

Exploratory analyses of rates of all-cause hospitalisation or death, systolic blood pressure change and systolic blood pressure control will be conducted by different levels of the following subgroups:

- baseline frailty (electronic frailty index score ≤0·12 vs >0.12 [fit vs. frail]), as in main trial analysis.

- baseline functional independence (Modified Rankin score ≤2 vs >2), as in main trial analysis.
- baseline cognitive function (MoCA score <26 vs ≥26), as in main trial analysis.
- number of antihypertensive medications prescribed at baseline (2 vs ≥3 medications)
- number of co-morbidities at baseline (≤4 vs >4 morbidities), as in main trial analysis.

29 VALIDATION

As a minimum the primary analysis will be validated by a Senior Trial Statistician (or delegate).

30 CHANGES TO THE PROTOCOL OR PREVIOUS VERSIONS OF SAP

31 References

1. **Statistics, Office for National.** *http://www.ons.gov.uk.* [Online] 2010.

2. *Epidemiology of multimorbidity and implications for health care, research, and medical education: a cross-sectional study.* **Barnett K, Mercer SW, Norbury M, Watt G, Wyke S, Guthrie B.** 9836, 2012, Lancet, Vol. 380, pp. 37-43.

3. *Prevalence of polypharmacy in a Scottish primary care population.* **Payne RA, Avery AJ, Duerden M, Saunders CL, Simpson CR, Abel GA.** 5, 2014, European journal of clinical pharmacology, Vol. 70, pp. 575-81.

4. *Potentially inappropriate prescribing among older people in the United Kingdom.* **Bradley MC, Motterlini N, Padmanabhan S, Cahir C, Williams T, Fahey T, Hughes CM.** 2014, BMC geriatrics, Vol. 14, p. 72.

5. *Polypharmacy and adverse drug reactions in Japanese elderly taking antihypertensives: a retrospective database study.* **Sato I, Akazawa M.** 2013, Drug, healthcare and patient safety, Vol. 5, pp. 143-50.

6. *Inappropriateness of medication prescriptions to elderly patients in the primary care setting: a systematic review.* **Opondo D, Eslami S, Visscher S, de Rooij SE, Verheij R, Korevaar JC, Abu-Hanna A.** 8, 2012, PloS one, Vol. 7, p. e43617.

7. *Burden of multimorbidity, socioeconomic status and use of health services across stages of life in urban areas: a cross-sectional study.* **Violan C, Foguet-Boreu Q, Roso-Llorach A, Rodriguez-Blanco T, Pons-Vigues M, Pujol-Ribera E, Munoz-Perez MA, Valderas JM.** 2014, BMC public health, Vol. 14, p. 530.

8. *Impact of age and sex on primary preventive treatment for cardiovascular disease in the West Midlands, UK: cross sectional study.* **Sheppard JP, Singh S, Fletcher K, McManus RJ, Mant J.** 2012, BMJ (Clinical research ed), Vol. 345, p. e4535.

9. *Treatment of hypertension in patients 80 years of age or older.* **Beckett NS, Peters R, Fletcher AE, Staessen JA, Liu L, Dumitrascu D, Stoyanovsky V, Antikainen RL, Nikitin Y, Anderson C, Belhani A, Forette F, Rajkumar C, Thijs L, Banya W, Bulpitt CJ.** 18, 2008, The New England journal of medicine, Vol. 358, pp. 1887-98.

10. *Treatment of hypertension in patients 80 years and older: the lower the better? A meta-analysis of randomized controlled trials.* **Bejan-Angoulvant T, Saadatian-Elahi M, Wright JM, Schron EB, Lindholm LH, Fagard R, Staessen JA, Gueyffier F.** 7, 2010, Journal of hypertension, Vol. 28, pp. 1366-72.

11. *Treatment With Multiple Blood Pressure Medications, Achieved Blood Pressure, and Mortality in Older Nursing Home Residents: The PARTAGE Study.* **Benetos A, Labat C, Rossignol P, Fay R, Rolland Y, Valbusa F, Salvi P, Zamboni M, Manckoundia P, Hanon O, Gautier S.** 2015, JAMA internal medicine.

12. *Antihypertensive medications and serious fall injuries in a nationally representative sample of older adults.* **Tinetti ME, Han L, Lee DS, McAvay GJ, Peduzzi P, Gross CP, Zhou B, Lin H.** 4, 2014, JAMA internal medicine, Vol. 174, pp. 588-95.

13. *Effects of benefits and harms on older persons' willingness to take medication for primary cardiovascular prevention.* **Fried TR, Tinetti ME, Towle V, O'Leary JR, Iannone L.** 10, 2011, Archives of internal medicine, Vol. 171, pp. 923-8.

14. *Lay perspectives on hypertension and drug adherence: systematic review of qualitative research.* **Marshall IJ, Wolfe CD, McKevitt C.** 2012, BMJ (Clinical research ed), Vol. 345, p. e3953.

15. *Deprescribing medication in very elderly patients with multimorbidity: the view of Dutch GPs. A qualitative study.* **Schuling J, Gebben H, Veehof LJ, Haaijer-Ruskamp FM.** 2012, BMC family practice, Vol. 13, p. 56.

16. *First do no harm: a real need to deprescribe in older patients.* **Scott IA, Anderson K, Freeman CR, Stowasser DA.** 7, 2014, The Medical journal of Australia, Vol. 201, pp. 390-2.

17. *Effect of Antihypertensive Medication Reduction vs Usual Care on Short-term Blood Pressure Control in Patients With Hypertension Aged 80 Years and Older The OPTIMISE Randomized Clinical Trial.* **Sheppard, James P., Burt, Jenni and al, Mark Lown et.** 20, s.l. : JAMA, 2020, Vol. 323.

32 Appendices

32.1 Appendix I. Flow chart of study activities up to 12 weeks follow-up

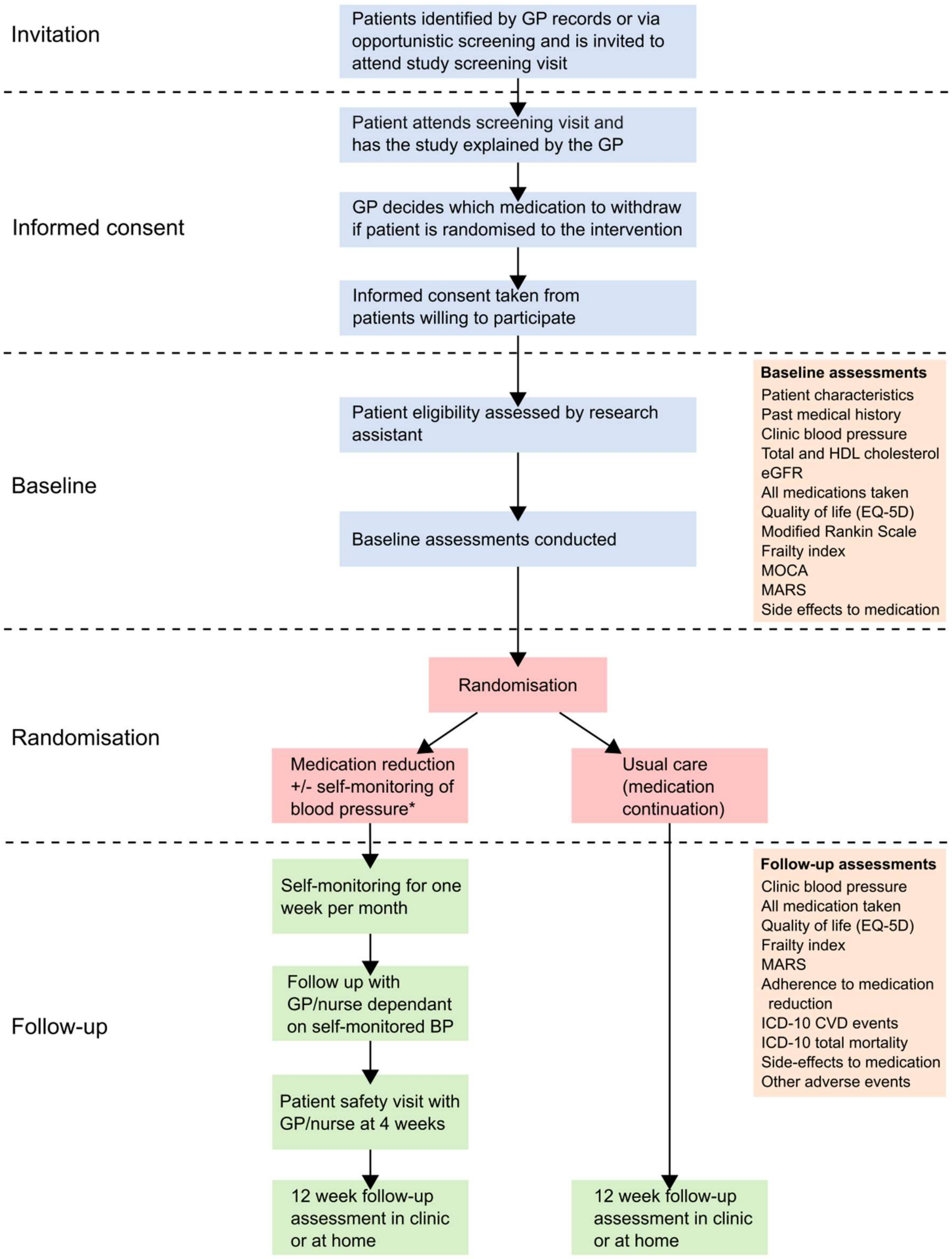

32.2 Appendix II. Outcome assessment schedule

	Screening	Baseline	1st time point post-randomisaton	2nd time point post-randomisation	3rd time point post-randomisation	….
Primary outcome						
xxx		x	x	x	x	
Secondary outcome						
xxx		x		x	x	
xxx		x	x			
…						
Loss of follow-up/withdrawal			x	x	x	
Adverse events			x	x	x	
Key covariates						
Demographics (age, gender etc)		x				
xxx						x

32.3 Appendix III. Flow diagram of trial participants

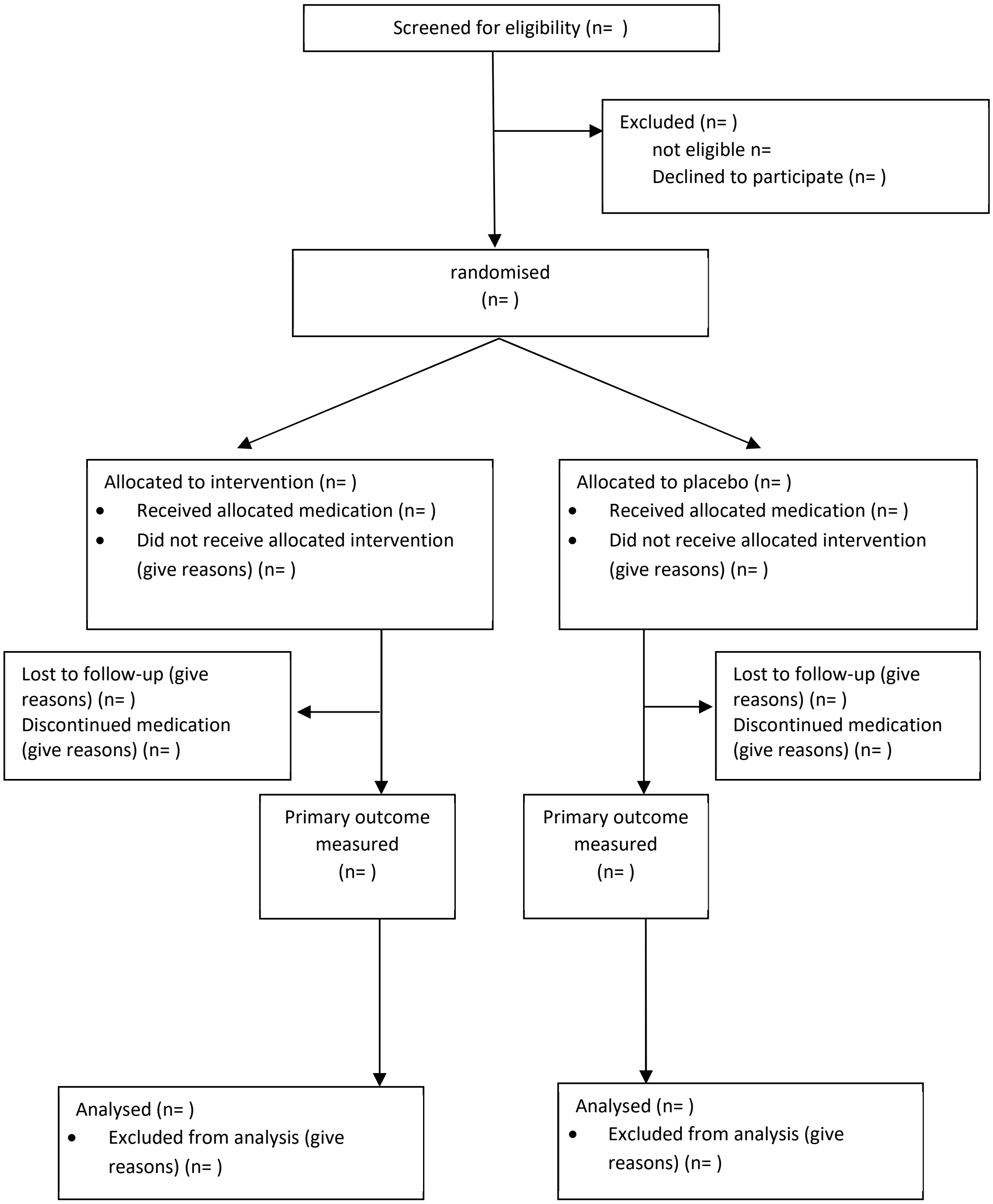

32.4 Appendix IV. Number of antihypertensive drugs in medications:

Please note: Some anti-hypertensive medications contain more than one anti-hypertensive drug combined in one tablet. Each anti-hypertensive drug should be counted individually (as indicated by the number in the brackets).

ACE inhibitors

		ACE inhibitors in combination medications	
Captopril (capoten)	(1)		
Cilazapril (vascace)	(1)		
Enalapril (Innovace)	(1)	Innozide = enalapril + hydrochlorothiazide	(2)
Fosinopril	(1)		
Lisinopril (carace, zestril)	(1)	Carace Plus / Zestoretic = lisinopril + hydrochlorthiazide	(2)
		Zestoretic = lisinopril + hydrochlorothiazide	(2)
Quinapril (accupril)	(1)	Accuretic = quinapril + hydrochlorothiazide	(2)
Perindopril (coversyl)	(1)	Conversyl Arginine Plus = perindopril + indapamide	(2)
Ramipril (tritace)	(1)	Triapin = ramipril + felodipine	(2)
Trandolapril	(1)		

Angiotensin II blockers

		ARB in combination medications	
Candesartan	(1)		
Irbesartan	(1)	CoAprovel = irbesartan + hydrochlorothiazide	(2)
Losartan	(1)	Cozaar-Comp = losartan + hydrochlorothiazide	(2)
Olmesartan	(1)	Olmetec plus = olmesartan + hydrochlorothiazide	(2)
Telmisartan	(1)	Micardis Plus = telmisartan + hydrochlorothiazide	(2)
Valsartan	(1)	Co-diovan = valsartan + hydrochlorothiazide	(2)
Eprosartan	(1)	Exforge = amlodipine + valsartan	(2)

Calcium Antagonists

		Calcium antagonists in combination medications	
Amlodipine (istin)	(1)	Sevikar = olmesartan medoxomil + amlodipine	(2)
		Sevikar HCT = olmesartan medoxomil + amlodipine + hydrochlorothiazide	(3)
		Exforge = amlodipine + valsartan	(2)
Diltizem (tildem, adizem, angtil, dilzem, tildiem, zemtard)	(1)		
Felodipine	(1)		
Lacidipine (motens)	(1)		
Nicardipine (cardene)	(1)		
Nifedipine (adalat, adipine, coracten)	(1)		
Verapamil (cordilox, securon)	(1)		
Lercanidipine	(1)		
Tildiem	(1)		
Diltiazem	(1)		
Adipine XL	(1)		
Verapamil	(1)		

Nifedipress MR	(1)
Securon	(1)
Zemtard	(1)

Thiazide & related Diuretics

Bendroflumethiazide (bendrofluazide)	(1)
Chlortalidone	(1)
Cyclopenthiazide (Navidrex)	(1)
Hydrochlorothiazide	(1)
Indapamide	(1)
Xipamide	(1)

In combination medications	
Co-triamterzide = bendrofluazide + triamterene	(2)
Co-tenidone = atenolol + chlortalidone	(2)

Beta blockers

Atenolol (tenormin, tenoret, adalat)	(1)
Bisoprolol (cardior, monocor)	(1)
Carvedilol (eucardic)	(1)
Metoprolol (betaloc, lopresor)	(1)
Nebivolol	(1)
Propanolol (inderal)	(1)
Sotalol	(1)
Propanolol	(1)
Nebivolol	(1)
Carvedilol	(1)

B-blockers in combination medications	
Co-tenidone = atenolol + chlortalidone	(2)

Potassium sparing diuretics

		Potassium sparing diuretics with other diuretics	
Amiloride	(1)	Co-amilofruse = amiloride + frusemide	(2)
		Burinex = amiloride + bumetanide	(2)
		Co-amilozide = amiloride + hydrochlorothiazide	(2)
Spironolactone (aldactone)	(1)	Co-flumactone = spironolactone +hydroflumethiazide	(2)
Triamterene	(1)	Co-triamterzide = triamterene + bendrofluazide (Dyazide)	(2)
		Dytide = triamterene + benzthiazide	(2)
		Frusene = triamterene + frusemide	(2)

Alpha 1 Blockers

Doxazosin (Cardura)	(1)
Prazosin	(1)
Terazosin	(1)
Indoramin	(1)
Tamsolosin/Tamsulosin	(1)

Centrally acting anti-hypertensives

Methyldopa	(1)
Moxonidine	(1)
Clonidine	(1)

Adrenergic neurone blocking drugs

Guanethidine	(1)
Debrisoquine	(1)

Vasodilator anti-hypertensives

Hydralazine	(1)

Direct renin inhibitors

Aliskiren	(1)

Loop diuretics

Bumetanide	(1)
Furosemide	(1)

Aldosterone antagonists

Eplerenone	(1)

32.5 Appendix V. List of ICD-10 codes to identify outcomes from NHS England data

Condition	ICD- 10 code	Description
Cardiovascular death	IX	Diseases of the circulatory system
Myocardial infarction	I21	Acute myocardial infarction
	I21.0	Acute transmural myocardial infarction of anterior wall
	I21.1	Acute transmural myocardial infarction of inferior wall
	I21.2	Acute transmural myocardial infarction of other sites
	I21.3	Acute transmural myocardial infarction of unspecified site
	I21.4	Acute subendocardial myocardial infarction
	I21.9	Acute myocardial infarction, unspecified
	I22	Subsequent myocardial infarction
	I22.0	Subsequent myocardial infarction of anterior wall
	I22.1	Subsequent myocardial infarction of inferior wall
	I22.8	Subsequent myocardial infarction of other sites
	I22.9	Subsequent myocardial infarction of unspecified site
	I24	Other acute ischaemic heart diseases
	I24.8	Other forms of acute ischaemic heart disease
	I24.9	Acute ischaemic heart disease, unspecified
Heart Failure	I50	Heart failure
	I50.0	Congestive heart failure
	I50.1	Left ventricular failure
	I50.9	Heart failure, unspecified
Stroke	I60	Subarachnoid haemorrhage
	I60.0	Subarachnoid haemorrhage from carotid siphon and bifurcation
	I60.1	Subarachnoid haemorrhage from middle cerebral artery
	I60.2	Subarachnoid haemorrhage from anterior communicating artery
	I60.3	Subarachnoid haemorrhage from posterior communicating artery
	I60.4	Subarachnoid haemorrhage from basilar artery
	I60.5	Subarachnoid haemorrhage from vertebral artery
	I60.6	Subarachnoid haemorrhage from other intracranial arteries
	I60.7	Subarachnoid haemorrhage from intracranial artery, unspecified
	I60.8	Other subarachnoid haemorrhage
	I60.9	Subarachnoid haemorrhage, unspecified
	I61	Intracerebral haemorrhage
	I61.0	Intracerebral haemorrhage in hemisphere, subcortical
	I61.1	Intracerebral haemorrhage in hemisphere, cortical
	I61.2	Intracerebral haemorrhage in hemisphere, unspecified
	I61.3	Intracerebral haemorrhage in brain stem

Condition	ICD- 10 code	Description
	I61.4	Intracerebral haemorrhage in cerebellum
	I61.5	Intracerebral haemorrhage, intraventricular
	I61.6	Intracerebral haemorrhage, multiple localized
	I61.8	Other intracerebral haemorrhage
	I61.9	Intracerebral haemorrhage, unspecified
	I62	Other nontraumatic intracranial haemorrhage
	I62.9	Intracranial haemorrhage (nontraumatic), unspecified
	I63	Cerebral infarction
	I63.0	Cerebral infarction due to thrombosis of precerebral arteries
	I63.1	Cerebral infarction due to embolism of precerebral arteries
	I63.2	Cerebral infarction due to unspecified occlusion or stenosis of precerebral arteries
	I63.3	Cerebral infarction due to thrombosis of cerebral arteries
	I63.4	Cerebral infarction due to embolism of cerebral arteries
	I63.5	Cerebral infarction due to unspecified occlusion or stenosis of cerebral arteries
	I63.6	Cerebral infarction due to cerebral venous thrombosis, nonpyogenic
	I63.8	Other cerebral infarction
	I63.9	Cerebral infarction, unspecified
	I64	Stroke, not specified as haemorrhage or infarction
Hypotension	I95	Hypotension
	I95.0	Idiopathic hypotension
	I95.1	Orthostatic hypotension
	I95.2	Hypotension due to drugs
	I95.8	Other hypotension
	I95.9	Hypotension, unspecified
Syncope	R55	Syncope and collapse
Fracture	S02	Fracture of skull and facial bones
	S02.0	Fracture of vault of skull
	S02.1	Fracture of base of skull
	S02.2	Fracture of nasal bones
	S02.3	Fracture of orbital floor
	S02.4	Fracture of malar and maxillary bones
	S02.5	Fracture of tooth
	S02.6	Fracture of mandible
	S02.7	Multiple fractures involving skull and facial bones
	S02.8	Fractures of other skull and facial bones
	S02.9	Fracture of skull and facial bones, part unspecified

Condition	ICD- 10 code	Description
	S12	Fracture of neck
	S12.0	Fracture of first cervical vertebra
	S12.1	Fracture of second cervical vertebra
	S12.2	Fracture of other specified cervical vertebra
	S12.7	Multiple fractures of cervical spine
	S12.8	Fracture of other parts of neck
	S12.9	Fracture of neck, part unspecified
	S22	Fracture of rib(s), sternum and thoracic spine
	S22.0	Fracture of thoracic vertebra
	S22.1	Multiple fractures of thoracic spine
	S22.2	Fracture of sternum
	S22.3	Fracture of rib
	S22.4	Multiple fractures of ribs
	S22.5	Flail chest
	S22.8	Fracture of other parts of bony thorax
	S22.9	Fracture of bony thorax, part unspecified
	S32	Fracture of lumbar spine and pelvis
	S32.0	Fracture of lumbar vertebra
	S32.1	Fracture of sacrum
	S32.2	Fracture of coccyx
	S32.3	Fracture of ilium
	S32.4	Fracture of acetabulum
	S32.5	Fracture of pubis
	S32.7	Multiple fractures of lumbar spine and pelvis
	S32.8	Fracture of other and unspecified parts of lumbar spine and pelvis
	S42	Fracture of shoulder and upper arm
	S42.0	Fracture of clavicle
	S42.1	Fracture of scapula
	S42.2	Fracture of upper end of humerus
	S42.3	Fracture of shaft of humerus
	S42.4	Fracture of lower end of humerus
	S42.7	Multiple fractures of clavicle, scapula and humerus
	S42.8	Fracture of other parts of shoulder and upper arm
	S42.9	Fracture of shoulder girdle, part unspecified
	S52	Fracture of forearm
	S52.0	Fracture of upper end of ulna
	S52.1	Fracture of upper end of radius
	S52.2	Fracture of shaft of ulna

Condition	ICD- 10 code	Description
	S52.3	Fracture of shaft of radius
	S52.4	Fracture of shafts of both ulna and radius
	S52.5	Fracture of lower end of radius
	S52.6	Fracture of lower end of both ulna and radius
	S52.7	Multiple fractures of forearm
	S52.8	Fracture of other parts of forearm
	S52.9	Fracture of forearm, part unspecified
	S62	Fracture at wrist and hand level
	S62.0	Fracture of navicular [scaphoid] bone of hand
	S62.1	Fracture of other carpal bone(s)
	S62.2	Fracture of first metacarpal bone
	S62.3	Fracture of other metacarpal bone
	S62.4	Multiple fractures of metacarpal bones
	S62.5	Fracture of thumb
	S62.6	Fracture of other finger
	S62.7	Multiple fractures of fingers
	S62.8	Fracture of other and unspecified parts of wrist and hand
	S72	Fracture of femur
	S72.0	Fracture of neck of femur
	S72.1	Pertrochanteric fracture
	S72.2	Subtrochanteric fracture
	S72.3	Fracture of shaft of femur
	S72.4	Fracture of lower end of femur
	S72.7	Multiple fractures of femur
	S72.8	Fractures of other parts of femur
	S72.9	Fracture of femur, part unspecified
	S82	Fracture of lower leg, including ankle
	S82.0	Fracture of patella
	S82.1	Fracture of upper end of tibia
	S82.2	Fracture of shaft of tibia
	S82.3	Fracture of lower end of tibia
	S82.4	Fracture of fibula alone
	S82.5	Fracture of medial malleolus
	S82.6	Fracture of lateral malleolus
	S82.7	Multiple fractures of lower leg
	S82.8	Fractures of other parts of lower leg
	S82.9	Fracture of lower leg, part unspecified
	S92	Fracture of foot, except ankle

Condition	ICD- 10 code	Description
	S92.0	Fracture of calcaneus
	S92.1	Fracture of talus
	S92.2	Fracture of other tarsal bone(s)
	S92.3	Fracture of metatarsal bone
	S92.4	Fracture of great toe
	S92.5	Fracture of other toe
	S92.7	Multiple fractures of foot
	S92.9	Fracture of foot, unspecified
	T02	Fractures involving multiple body regions
	T02.0	Fractures involving head with neck
	T02.1	Fractures involving thorax with lower back and pelvis
	T02.2	Fractures involving multiple regions of one upper limb
	T02.3	Fractures involving multiple regions of one lower limb
	T02.4	Fractures involving multiple regions of both upper limbs
	T02.5	Fractures involving multiple regions of both lower limbs
	T02.6	Fractures involving multiple regions of upper limb(s) with lower limb(s)
	T02.7	Fractures involving thorax with lower back and pelvis with limb(s)
	T02.8	Fractures involving other combinations of body regions
	T02.9	Multiple fractures, unspecified
	T08	Fracture of spine, level unspecified
	T10	Fracture of upper limb, level unspecified
	T12	Fracture of lower limb, level unspecified
	T14.2	Fracture of unspecified body region
Fall	W01	Fall on same level from slipping, tripping and stumbling
	W05	Fall involving wheelchair
	W06	Fall involving bed
	W07	Fall involving chair
	W08	Fall involving other furniture
	W10	Fall on and from stairs and steps
	W17	Other fall from one level to another
	W18	Other fall on same level
	W19	Unspecified fall
Dementia	F00	Dementia in Alzheimer disease
	F00.0	Dementia in Alzheimer disease with early onset
	F00.1	Dementia in Alzheimer disease with late onset
	F00.2	Dementia in Alzheimer disease, atypical or mixed type
	F00.9	Dementia in Alzheimer disease, unspecified
	F01	Vascular dementia

Condition	ICD- 10 code	Description
	F01.0	Vascular dementia of acute onset
	F01.1	Multi-infarct dementia
	F01.2	Subcortical vascular dementia
	F01.3	Mixed cortical and subcortical vascular dementia
	F01.8	Other vascular dementia
	F01.9	Vascular dementia, unspecified
	F02	Dementia in other diseases classified elsewhere
	F02.0	Dementia in Pick disease
	F02.1	Dementia in Creutzfeldt-Jakob disease
	F02.2	Dementia in Huntington disease
	F02.3	Dementia in Parkinson disease
	F02.4	Dementia in human immunodeficiency virus [HIV] disease
	F02.8	Dementia in other specified diseases classified elsewhere
	F03	Unspecified dementia
Acute kidney injury	S37.0	Injury of kidney
	N19	Unspecified kidney failure
	N17	Acute renal failure
	N17.0	Acute renal failure with tubular necrosis
	N17.1	Acute renal failure with acute cortical necrosis
	N17.2	Acute renal failure with medullary necrosis
	N17.8	Other acute renal failure
	N17.9	Acute renal failure, unspecified
	866	kidney injury*
	866	kidney injury-closed*
	866	kidney injury nos-closed
	586	renal failure nos
	584	acute renal failure*
	584.5	ac kidny fail, tubr necr
	584.6	ac kidny fail, cort necr
	584.7	ac kidny fail, medu necr
	584.8	acute kidney failure nec
	584.9	acute kidney failure nos
Electrolyte abnormalities	276	fluid/electrolyte dis*
	276	hyperosmolality
	276.1	hyposmolality
	276.2	acidosis
	276.3	alkalosis
	276.4	mixed acid-base bal dis

Condition	ICD- 10 code	Description
	276.5	hypovolemia#
	276.6	fluid overload#
	276.7	hyperpotassemia
	276.8	hypopotassemia
	276.9	electrolyt/fluid dis nec
	E87	Other disorders of fluid, electrolyte and acid-base balance
	E87.0	Hyperosmolality and hypernatraemia
	E87.1	Hypo-osmolality and hyponatraemia
	E87.2	Acidosis
	E87.3	Alkalosis
	E87.4	Mixed disorder of acid-base balance
	E87.5	Hyperkalaemia
	E87.6	Hypokalaemia
	E87.7	Fluid overload
	E87.8	Other disorders of electrolyte and fluid balance, not elsewhere classified

Table S1. Inclusion and exclusion criteria for the trial

**Inclusion criteria**
• Participant is willing and able to give informed consent for participation in the trial.
• Male or Female, aged 80 years or above.
• Clinic systolic blood pressure less than 150 mmHg (according to screening measurement at baseline – clinic blood pressure defined as the mean of the 2nd and 3rd readings taken at 1 minute intervals).
• Prescribed two or more antihypertensive medications to lower blood pressure for at least 12 months prior to trial entry. Antihypertensive medications defined as any ACE inhibitor, angiotensin II receptor blocker, calcium channel blocker, thiazide and thiazide-like diuretic, potassium-sparing diuretic, alpha-blocker, beta-blocker, vasodilator antihypertensives, centrally acting antihypertensives, direct renin inhibitors, adrenergic neurone blocking drugs or loop diuretics.
• Stable dose of antihypertensive medications for at least four weeks prior to trial entry.
• In the Investigator's opinion, could potentially benefit from medication reduction due to existing polypharmacy, co-morbidity, non-adherence or dislike of medicines and/or frailty
• In the Investigator's opinion, is able and willing to comply with all trial requirements.
**Exclusion criteria**
• A participant has heart failure due to LVSD and is on only ACE inhibitors/ARBs and/or beta-blockers and/or spironolactone (removing any of which would be contraindicated).
• A participant has heart failure but has not had an echocardiogram since its onset (might have undiagnosed LVSD and a compelling need for ACE inhibitors/ARB and Beta-blockers).
• Investigator deems that there is a compelling indication for antihypertensive medication continuation.
• Any other significant disease or disorder which, in the opinion of the Investigator, may either put the participants at risk because of participation in the trial, or may influence the result of the trial, or the participant's ability to participate in the trial (e.g. terminal illness, house bound and unable to attend baseline and follow up clinics).
• Suffered a myocardial infarction or stroke within the past 12 months.
• Blood pressure being managed outside of primary care.
• Unable to provide consent due to incapacity.
• A participant with secondary hypertension or previous accelerated or malignant hypertension.
• Participants who have participated in another research trial involving antihypertensive medication in the past 4 weeks.

Figure S1. Flow of participants through the study

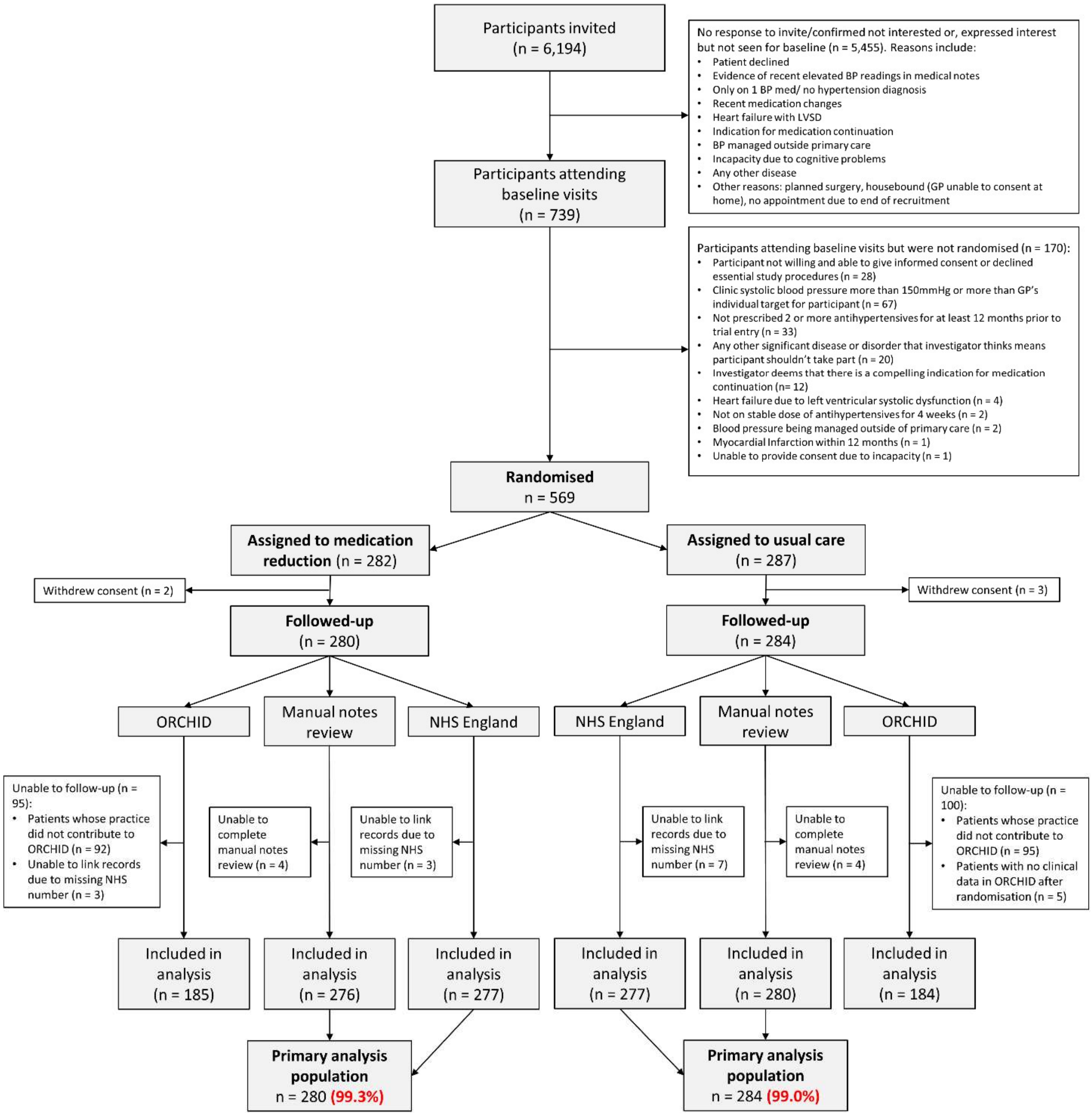

Table S2. Binary analyses of clinical outcomes at follow-up (intention to treat analyses) in those alive at 2 years 6 months post-randomisation

	Medication reduction group n=280*	Usual care Group n=284*	Adjusted risk ratio (95% CI)†	P-value
**Primary outcome**				
All-cause hospitalisation or mortality (n, %)	177/280 (63·2%)	177/284 (62·3%)	1·02 (0·90 to 1·15)	0·80
**Secondary outcomes**				
All-cause hospitalisation (n, %)	163/278 (58·6%)	156/281 (55·2%)	1·06 (0·92 to 1·22)	0·45
All-cause mortality (n, %)	31/280 (11·1%)	33/284 (11·6%)	0·94 (0·60 to 1·50)	0·81
Emergency hospitalisation (n, %)	105/274 (38·3%)	90/276 (32·6%)	1·17 (0·93 to 1·47)	0·18
Major cardiovascular events (n, %)	39/267 (14·6%)	37/268 (13·8%)	1·03 (0·68 to 1·57)	0·88
Myocardial infarction (n, %)	12/259 (4·6%)	12/256 (4·7%)	0·96 (0·44 to 2·10)	0·92
Stroke (n, %)	8/257 (3·1%)	10/261 (3·8%)	0·78 (0·31 to 1·96)	0·60
Dementia (n, %)	9/248 (3·6%)	5/247 (2·0%)	1·75 (0·59 to 5·17)	0·31
Hypotension (n, %)	17/259 (6·6%)	8/256 (3·1%)	2·04 (0·90 to 4·65)	0·09
Syncope (n, %)	1/254 (0·4%)	0/254 (0·0%)	-	
Falls (n, %)	0/254 (0·0%)	1/254 (0·4%)	-	
Fracture (n, %)	2/256 (0·8%)	1/254 (0·4%)	2·16 (0·20 to 23·56)	0·89
Electrolyte abnormalities (n, %)	25/262 (9·5%)	21/261 (8·0%)	1·16 (0·67 to 2·01)	0·95
Acute kidney injury (n, %)	21/260 (8·1%)	20/263 (7·6%)	1·02 (0·57 to 1·82)	0·94

* The denominator for each binary outcome do not include those who did not have the event of interest and died before 2 years 6 months post-randomisation (those who had the event but died sooner than 2 years 6 months post-randomisation were still included in the denominator and recorded as having had the event).

† Adjusted relative risks (aRR), derived from either generalised missed effect model, adjusting for baseline systolic blood pressure, and including practice as a random effect. aRR < 1 favours medical reduction. CI = confidence interval

Table S3. Post-hoc sensitivity analyses of outcomes occurring prior to the first UK lockdown for the Coronavirus Pandemic on 23rd March 2020 and the primary outcome according to participant sex

	Medication reduction group	Usual care Group	Adjusted hazard ratio (95% CI)*	P-value
**Outcomes occurring prior to 23rd March 2020**				
**All-cause hospitalisation or mortality**	**n=280**	**n=284**		
Intention to treat analysis (n, %)	150 (53·6%)	149 (52·5%)	1·02 (0·81 to 1·28)	0·90
Time at risk (years; incidence rate)	377·8 (39·7)	382·0 (39·0)		
**All-cause mortality**				
Intention to treat analysis (n, %)	23 (8·2%)	23 (8·1%)	1·00 (0·56 to 1·78)	0·99
Time at risk (years; incidence rate)	534·3 (4·3)	541·7 (4·3)		
**Outcomes according to participant sex**				
**All-cause hospitalisation or mortality (Female)**	**n=130**	**n=143**		
Intention to treat analysis (n, %)	93 (71·5%)	100 (69·9%)	1·03 (0·78 to 1·37)	
Time at risk (years; incidence rate)	275·3 (33·8)	314·2 (31·8)		
**All-cause hospitalisation or mortality (Male)**	**n=150**	**n=141**		
Intention to treat analysis (n, %)	109 (72·7)	118 (83·7)	0·83 (0·64 to 1·08)	
Time at risk (years; incidence rate)	317·7 (34·3)	280·3 (42·1)	Test of interaction (p-value):	0·78

* Cox proportional hazards model for the analysis of the primary outcome, adjusting baseline systolic blood pressure and intervention group as fixed effects, including GP practice as a random effect. Hazard ration (HR) <1 indicates favour to medication reduction group. CI = confidence interval

Figure S2. Rates of all-cause hospitalisation or death by calendar time

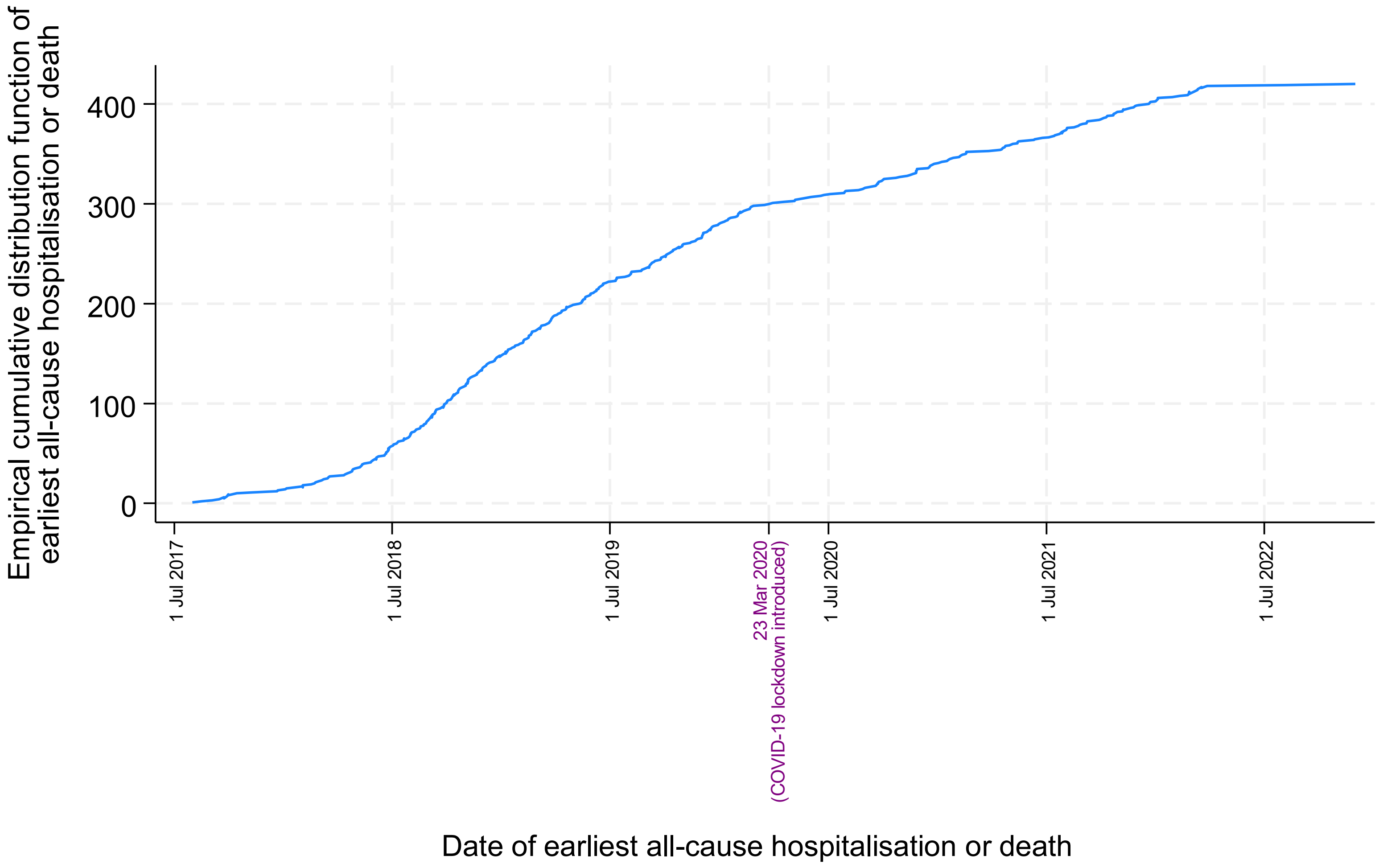

Table S4. Primary care consultations related to hypertension during follow-up

	Medication reduction group n=251*	Usual care Group n=250*	Adjusted relative risk/difference (95% CI)	P-value
**All Primary care consultations**				
Attended at least 1 consultation, n(%)	215 (86%)	212 (85%)	1·01 (0·94 – 1·09)†	0·76
Mean number of consultations attended‡ (SD) [range]	4·4 (3·12) [1 – 19]	3·6 (3·05) [1 – 22]	1·23 (1·08 – 1·40) §	0·0018
**General Practitioner consultations**				
Attended at least 1 consultation, n(%)	172 (69%)	133 (53%)	1·30 (1·13 – 1·50)†	0·0003
Mean number of consultations attended‡ (SD) [range]	2·7 (2·13) [1 –12]	2·7 (2·65) [1 – 17]	1·02 (0·86 – 1·22) §	0·82
**Practice nurse consultations**				
Attended at least 1 consultation, n(%)	133 (53%)	93 (37%)	1·39 (1·16 – 1·67)†	0·0005
Mean number of consultations attended‡ (SD) [range]	2·2 (1·62) [1 – 9]	2·0 (1·39) [1 – 7]	1·12 (0·93 – 1·36) §	0·24
**Pharmacist consultations**				
Attended at least 1 consultation, n(%)	12 (5%)	15 (6%)	0·88 (0·49 – 1·58)†	0·68
Mean number of consultations attended‡ (SD) [range]	1·9 (1·62) [1 – 6]	3·2 (2·86) [1 – 9]	0·70 (0·42 – 1·18) §	0·18
**Other consultations**				
Attended at least 1 consultation, n(%)	77 (31%)	91 (36%)	0·93 (0·82 – 1·07)†	0·32
Mean number of consultations attended‡ (SD) [range]	2·0 (1·28) [1 – 8]	1·9 (1·14) [1 – 6]	1·08 (0·87 – 1·34) ll	0·50

* Only participants who were still alive 2 years 6 months post-randomisation were included in these analyses.
† Adjusted relative risks (aRR), derived from mixed effect logistic regression model, adjusting for baseline systolic blood pressure, and including practice as a random effect. aRR < 1 favours medical reduction.
‡ Denominator is those attending at least one consultation.
§ Adjusted relative difference (aRD), derived from negative binomial regression model, adjusting for baseline systolic blood pressure as fixed effects and including practice as a random effect. aRD < 1 favours medication reduction.
ll Adjusted relative difference (aRD), derived from negative binomial regression model, adjusting for baseline systolic blood pressure as fixed effects. aRD < 1 favours medication reduction.

Table S5. Maintenance of medication prescription strategies during follow-up

Change in medication prescription	Medication reduction group n=213*	Usual care group n=207*	Adjusted risk ratio Intervention vs. Control (95% CI)†	P-value
Decreased antihypertensive prescription, n (%)	109 (51·2%)	51 (24·6%)	3·35 (2·17 to 5·19)	<0·001
Maintained antihypertensive prescription, n (%)	80 (37·6%)	128 (61·8%)	-	-
Increased antihypertensive prescription, n (%)	18 (8·5%)	20 (9·7%)	1·46 (0·73 to 2·94)	0·287
Missing, n (%)	6 (2·8%)	8 (3·9%)	-	-

* Analysis only includes those patients still alive at follow-up

† Risk ratio of a change in medication prescription at follow-up. Value above 1 indicates a change is more likely in the intervention group, compared with maintaining the same number of medications prescribed at baseline (reference category).

Figure S3. Antihypertensive medication prescription changes over time, in participants registered to practices contributing to the ORCHID who were alive at least 4 years of follow-up (n=278). Error bars indicate 95% confidence intervals.

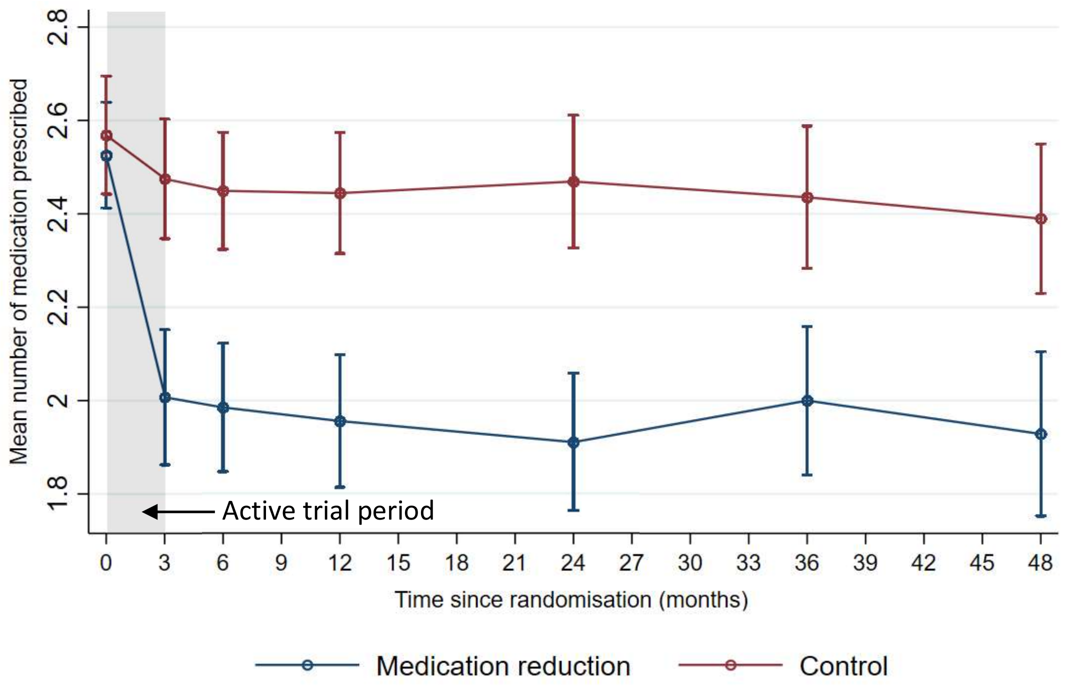

Table S6. Blood pressure prior to baseline, at baseline and during follow-up, in participants registered to practices contributing to the ORCHID (n=369).

Timepoint	Medication reduction (N=185)		Control (N=184)		Adjusted mean difference*	P-value
	n (% of total randomised)	Mean (SD)	n (% of total randomised)	Mean (SD)	Intervention vs. Control (95% CI)	
**Systolic blood pressure (mm Hg)**						
**preBaseline**	185 (100%)	133·4 (11·8)	183 (100%)	134·7 (13·2)	-	
**Baseline**	185 (100%)	128·9 (13·5)	184 (100%)	131·1 (11·7)	-	
**3-month**	177 (96%)	134·5 (16·2)	158 (86%)	132·3 (12·7)	3·32 (0·25 to 6·39)	0·03
**6-month**	130 (70%)	139·5 (15·7)	125 (68%)	136·1 (15·7)	4·31 (0·82 to 7·80)	0·02
**12-month**	145 (78%)	137·4 (16·8)	129 (70%)	136·2 (14·1)	1·78 (-1·60 to 5·16)	0·30
**24-month**	138 (75%)	138·8 (15·1)	131 (71%)	139·8 (17·1)	-0·04 (-3·45 to 3·37)	0·98
**36-month**	144 (78%)	139·8 (17·2)	123 (67%)	138·6 (15·3)	2·48 (-0·95 to 5·91)	0·16
**48-month**	112 (61%)	137·1 (14·4)	105 (57%)	138·7 (18·1)	0·04 (-3·75 to 3·82)	0·99
**Diastolic blood pressure (mm Hg)**						
**preBaseline**	185 (100%)	70·2 (9·1)	183 (100%)	71·3 (9·0)	-	
**Baseline**	185 (100%)	67·7 (9·2)	184 (100%)	69·6 (8·5)	-	
**3-month**	177 (96%)	70·7 (10·4)	158 (86%)	70·6 (9·1)	1·07 (-0·82 to 2·95)	0·63
**6-month**	130 (70%)	73·4 (8·5)	125 (68%)	71·5 (9·7)	2·78 (0·63 to 4·92)	0·01
**12-month**	145 (78%)	71·6 (11·1)	129 (70%)	71·9 (9·4)	0·56 (-1·52 to 2·63)	0·60
**24-month**	138 (75%)	72·5 (11·0)	131 (71%)	72·3 (10·4)	1·10 (-1·00 to 3·19)	0·30
**36-month**	144 (78%)	72·5 (10·4)	123 (67%)	72·8 (9·2)	0·55 (-1·56 to 2·65)	0·60
**48-month**	112 (61%)	72·1 (10·4)	105 (57%)	71·6 (10·6)	1·49 (-0·83 to 3·81)	0·21

* Linear mixed effects model including baseline systolic blood pressure and randomised group (fixed effects) and primary care site (random effect).
SD = Standard deviation; CI = confidence interval

Figure S4. Blood pressure changes over time, in participants registered to practices contributing to the ORCHID (n=369). Error bars indicate 95% confidence intervals.

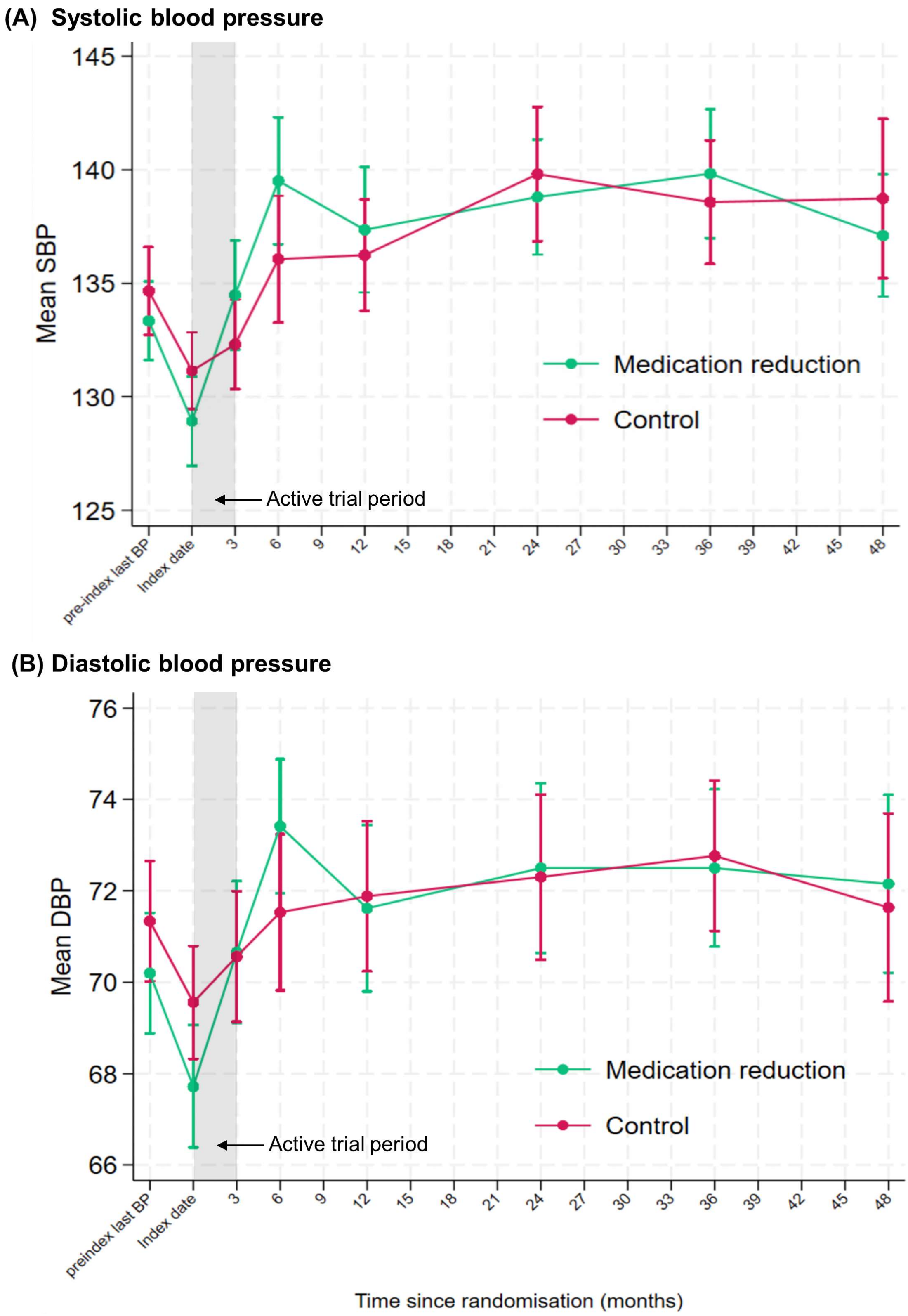

Figure S5. Blood pressure changes over time, in participants registered to practices contributing to the ORCHID who were alive at least 4 years of follow-up (n=278). Error bars indicate 95% confidence intervals.

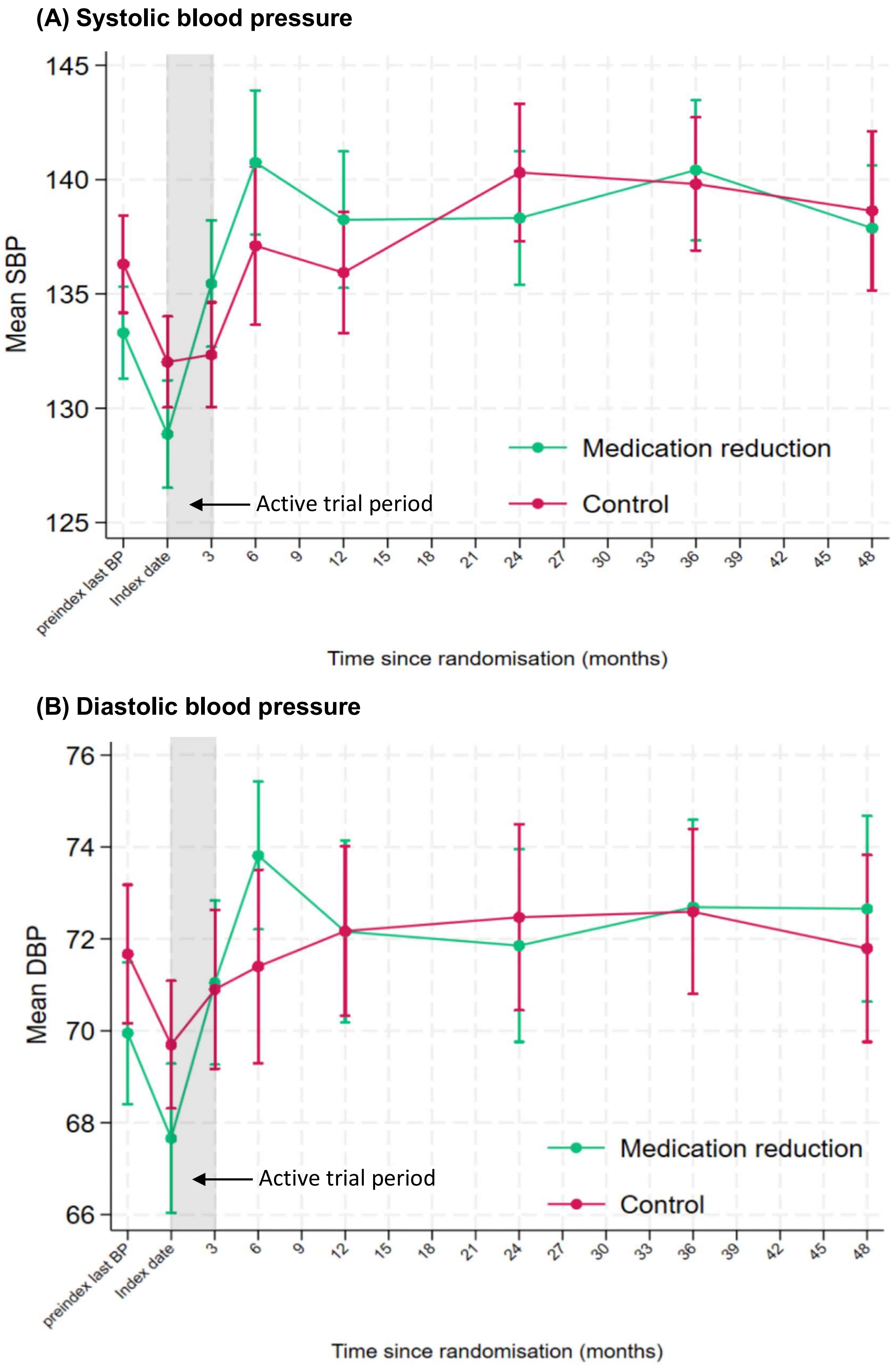

Figure S6. Hazard ratio of time to all-cause hospitalisation or death by prespecified subgroups.

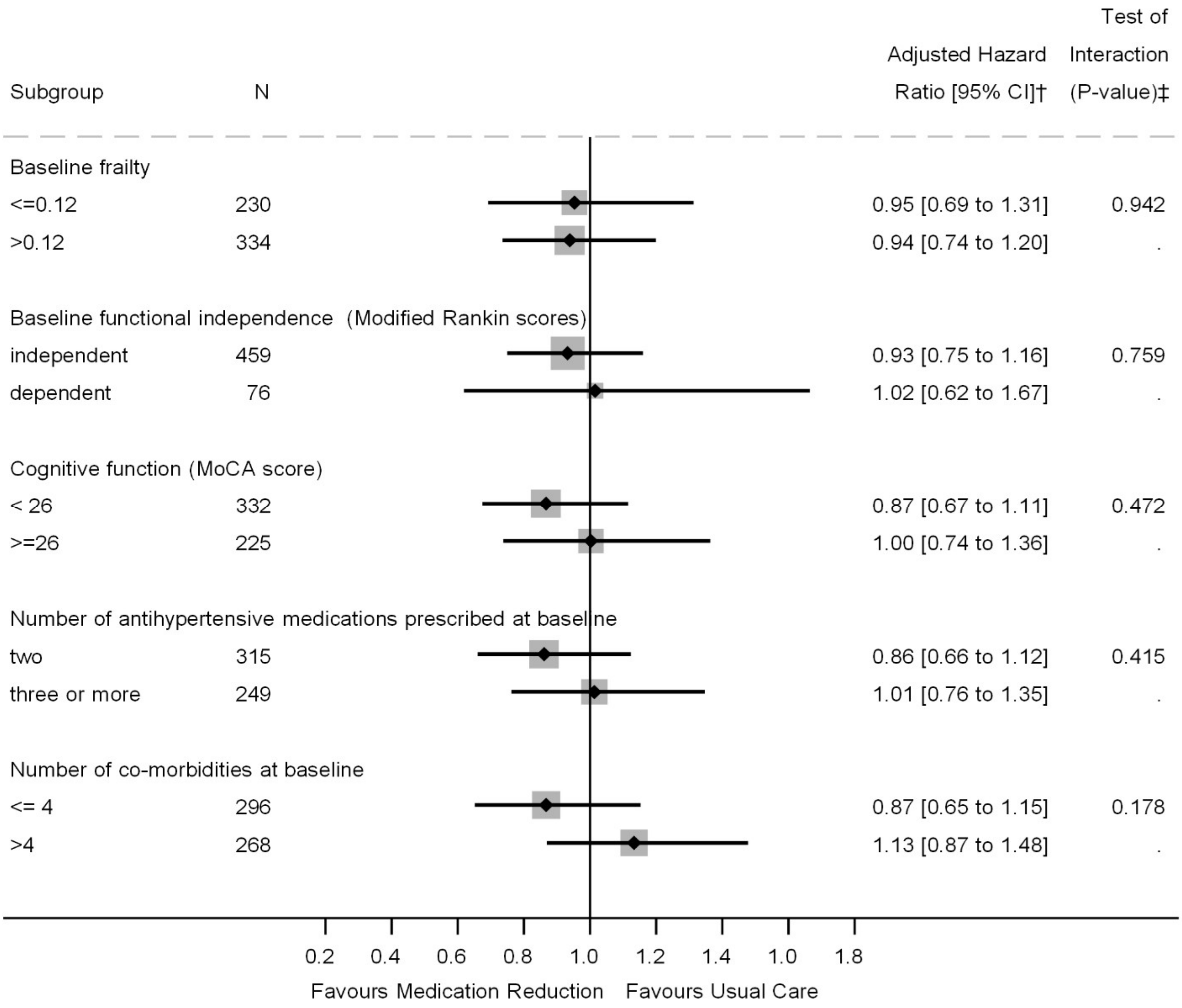

†Medication reduction versus usual care.
‡Level of significance = 0.05

Figure S7. Relative risk of systolic blood pressure control at 3-year follow-up by prespecified subgroups

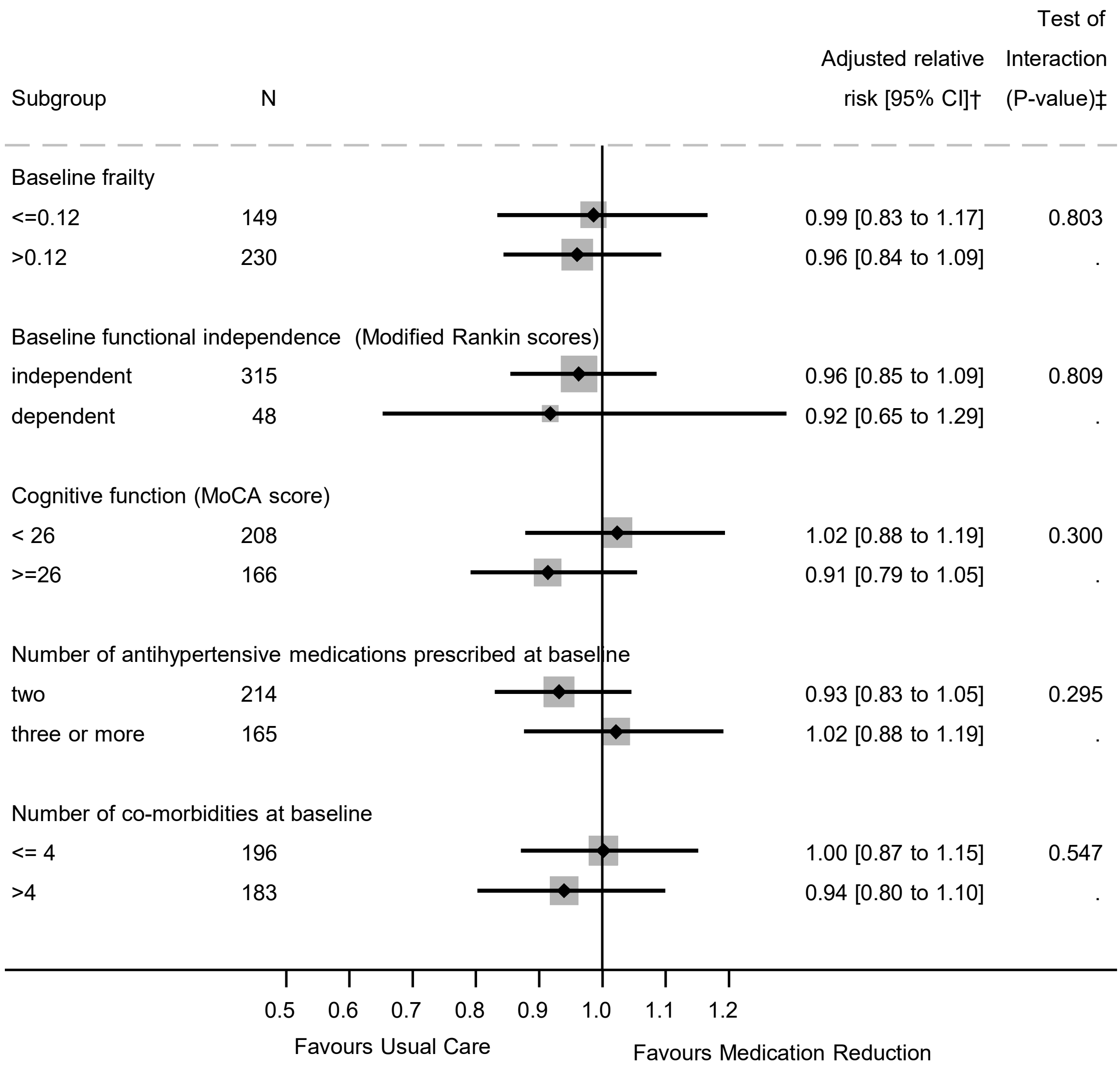

†Medication reduction versus usual care
‡Level of significance = 0.05

Figure S8. Mean difference in change in systolic blood pressure (mm Hg) at 3-year follow-up by prespecified subgroups

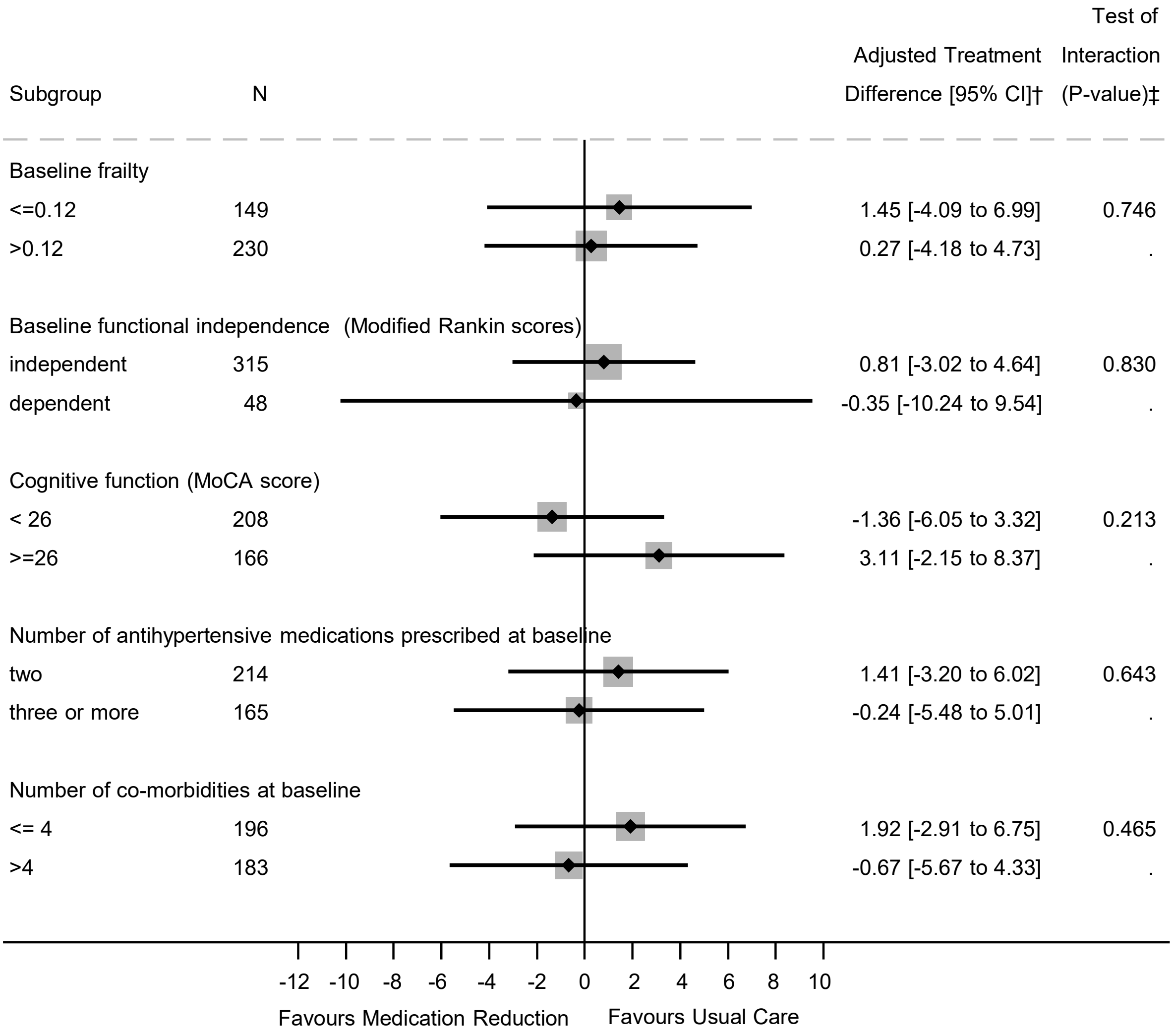

†Medication reduction versus usual care
‡Level of significance = 0.05
